# Physical therapist management of patients with suspected or confirmed osteoporosis – German translation and contextual adaptation of a clinical practice guideline from the Academy of Geriatric Physical Therapy (USA)

**DOI:** 10.3389/fresc.2026.1933821

**Published:** 2026-10-12

**Authors:** Tobias Braun, Maxime Hermanns, Vanessa Haug, Jessica Nau, Bettina Scheffler, Gudrun Diermayr, Gregory W. Hartley, Keith G. Avin, Susanne Saal

**Affiliations:** 1 Department of Health, HSD Hochschule Döpfer, Cologne, Germany; 2 Praxis für Physiotherapie, Isla Vitalis, Sankt Augustin, Germany; 3 Institute for Geriatric Research, University Hospital Ulm, AGAPLESION Bethesda Clinic Ulm, Ulm, Germany; 4 Department of Physiotherapy, Brandenburg University of Technology Cottbus-Senftenberg, Senftenberg, Germany; 5 School of Health, Education and Social Sciences, SRH University, Heidelberg, Germany; 6 Department of Physical Therapy, University of Miami, Miller School of Medicine, Coral Gables, FL, United States; 7 Department of Physical Therapy, School of Health & Human Sciences, Indiana University, Indianapolis, IN, United States; 8 Department of Health and Nursing, Ernst Abbe University of Applied Sciences Jena (EAH), Jena, Germany

**Keywords:** ADAPTE, exercise, guideline [MeSH], implementation, osteoporosis, physiotherapy, rehabilitation

## Abstract

**Introduction:**

Osteoporosis is one of the major causes of fractures in individuals over the age of 50 years worldwide and an increasing public health problem. Trustworthy evidence indicates that physical exercise can reduce the risk of fall-related fractures in people with osteoporosis. Patients with suspected or confirmed osteoporosis are frequently referred to physiotherapy to address fall risk, bone health, pain, range of motion, and physical functioning. In Germany, there was no specific guidance on physiotherapeutic management for patients with osteoporosis. Thus, we aimed to identify, translate and adapt the most appropriate international physiotherapy guideline for the management of patients with osteoporosis.

**Methods:**

The guideline adaptation process was informed by internationally accepted methodological frameworks and included six modules. We conducted a systematic review of the literature to identify relevant guideline(s) for adaptation (module 1), assessed guideline quality with AGREE-II (module 2), decided on one source guideline according to prespecified criteria (module 3), translated this guideline into German (module 4), reviewed and revised several guideline drafts by means of an expert committee, 3 physiotherapists and 2 older individuals with osteoporosis and adapted the guideline to the German healthcare context (module 5), and initiated implementation and dissemination of the final German guideline version (module 6).

**Results:**

In total, we screened 873 records from databases and via other methods, and finally selected the “APTA Geriatrics’ (Academy of Geriatric Physical Therapy, American Physical Therapy Association, USA) guideline published in 2022. Expert, end-user, and patient feedback (module 5) was used to make minor adaptations to the guideline (e.g., adding a glossary). We did not change any recommendation concerning physiotherapy management. A final German version of the guideline was approved by the whole research team and the developers of the source guideline and is now available open access (https://osf.io/yav94/files/s357b).

**Conclusion:**

The APTA Geriatrics osteoporosis guideline was translated and adapted for German-speaking countries through a structured, participatory process. The guideline can serve as the basis for evidence-based physiotherapy in German-speaking countries.

**Trial registration:**

The study protocol was published prospectively (https://doi.org/10.17605/OSF.IO/TYXGF).

## Background

Osteoporosis is defined as a “progressive systemic skeletal disease characterised by low bone mass and microarchitectural deterioration of bone tissue, with a consequent increase in bone fragility and susceptibility to fracture” (1). Osteoporosis is one of the significant fundamental causes of fractures in individuals over the age of 50 years worldwide and represents an increasing public health problem (1). In the EU, 22.1 million women and 6.6 million men (aged 50 + years) were estimated to be affected by osteoporosis in 2019, with 4.3 million new fragility fractures reported (e.g., hip, vertebral, and forearm fractures) (2). Because of the systemic nature of osteoporosis, the associated increase in fracture risk affects virtually all skeletal sites (3). However, hip fractures and vertebral fractures have historically been considered the prototypical osteoporotic fractures, with an estimated 2.7 million hip fractures occurring in 2010 worldwide (4). The lifetime risk of any osteoporotic fracture lies within the range of 40%–50% in women and 13%–22% for men (5).

In Germany, 4.5 million women (22.6%) and 1.2 million men (6.6%) are affected by osteoporosis, accounting for 6.1% of the total population (2). According to the simulation model reported by Bleibler et al. (6), the number of fractures attributable to osteoporosis will rise from 115,000 in 2010 to 274,000 in 2050, cumulating to approximately 8.1 million fractures during this period (78% women, 22% men). The discounted cumulated costs from 2010 to 2050 will amount to 88.5 billion Euros (6).

The main objective of treating osteoporosis is to reduce the likelihood of fragility fractures by strengthening the skeleton, decreasing fall frequency, or both (4). Lifestyle measures such as adequate nutrition, regular physical exercise, and avoidance of harmful habits are recommended for all individuals with suspected or confirmed osteoporosis (4, 7). Physical exercise involves any bodily movement produced by skeletal muscles that expends energy and is positively correlated with physical fitness as the intensity, duration, and frequency of movements increase. Physical exercise is a physical activity that is planned, structured, repetitive, and purposive in the sense that improvement or maintenance of one or more components of physical fitness is an objective (8). A significant body of evidence indicates that physical exercise can strengthen the muscular system and reduce the risk of falls and fall-related fractures in older adults, including those with osteoporosis (9–12).

Physical exercises for patients with osteoporosis can be instructed and/or supervised by physiotherapists (7). “Physiotherapy is a service provided by physiotherapists to individuals and populations to develop, maintain and restore maximum movement and functional ability throughout the lifespan. The service is provided in circumstances where movement and function are threatened by ageing, injury, pain, diseases, disorders, conditions and/or environmental factors and with the understanding that functional movement is central to what it means to be healthy” (13). Patients with suspected or confirmed osteoporosis are frequently referred to physiotherapy for physical exercises to address common risk factors of falls, such as balance impairment, low muscle strength/sarcopenia, and mobility impairment (14, 15). Other therapy goals for individuals with osteoporosis include pain relief, improved posture, enhanced range of motion, and improved physical functioning (14, 16). Physiotherapists are responsible for ensuring that patient management is based on the best available current evidence, including external evidence such as clinical practice guidelines (17).

Clinical practice guidelines (CPG, hereafter referred to as guidelines) are 'systematically developed statements to assist practitioners and patients in making decisions about appropriate healthcare for specific clinical circumstances' (18). Guidelines are intended to reduce the research-practice gap and should be based on the best available scientific evidence to improve the quality of patient care (19). Guidelines can focus on interdisciplinary patient management or provide recommendations specific to a particular health profession (referred to as “professional guidelines'). For the care of patients with osteoporosis, many international, interdisciplinary guidelines are available, including recommendations on prevention, diagnosis and interventions (20–22). However, these multidisciplinary guidelines do not provide specific and/or consistent recommendations for physiotherapists (20, 21). Moreover, Coronado-Zarco et al. (22) reviewed CPG to identify recommendations on nonpharmacological interventions for osteoporosis treatment. Only 16% of the recommendations in the six included guidelines were for exercise (17% for vitamin D and 16% for a combination of calcium and vitamin D).

At the national level, the most current and interdisciplinary 2023 German guideline on the prevention, diagnosis and therapy of osteoporosis (Dachverband Osteologie 2023), developed under the leadership of several medical organisations, includes only a few rather general statements on the physiotherapeutic management of patients with osteoporosis. Additionally, the 2009 German “Guideline Physiotherapy and Exercise Therapy in Osteoporosis' (23) has never been updated. Hence, there is currently no specific German-language physiotherapy guideline for the management of patients with osteoporosis (24). Thus, there is currently a lack of specific guidance on physiotherapy management for patients with osteoporosis in Germany. However, several recent and credible guidelines on physiotherapy, exercise and rehabilitation in osteoporosis have been published that may be suitable for adaptation to the German healthcare context (21).

The development of high-quality CPGs requires substantial time, expertise and resources (25). To enhance efficiency in guideline development and improve utilisation, the approach of guideline adaptation (i.e., adapting an existing, high-quality guideline to a new context) is deemed suitable for addressing the lack of a specific physiotherapy guideline for osteoporosis in Germany. Thus, we aimed to identify, translate and adapt the most appropriate (in terms of quality, currency, and transferability) international physiotherapy guideline for the management of patients with osteoporosis in the German healthcare context.

## Methods

The process of guideline adaptation was informed by the framework developed by the ADAPTE Collaboration (25), the methods paper for adapting international physiotherapy guidelines of the German Society for Physiotherapy Science (DGPTW; based on the ADAPTE framework) (26) and the guidelines for the process of cross-cultural adaptation of self-report measures by Beaton et al. (27).

The study was approved by the Ethical Review Board of the HSD Hochschule Döpfer GmbH, Germany (registration number: HSDEK_BA_2025_02). All participants provided written informed consent before participating in the study. The study was conducted in accordance with the ethical standards outlined in the World Medical Association Declaration of Helsinki guidelines.

The study protocol was published prospectively in the Open Science Framework (https://doi.org/10.17605/OSF.IO/TYXGF).

The study was conducted by completing six consecutive modules [modified from the ADAPTE approach (25)] and several steps within each module. Figure 1 illustrates the study procedures. However, due to limited project resources, we did not follow all steps as recommended by the ADAPTE collaboration (25). For example, we did not determine health questions (ADAPTE phase 2, step 7), and we did not reassess the supporting evidence for each recommendation (ADAPTE phase 2, step 15).

**Figure 1:**
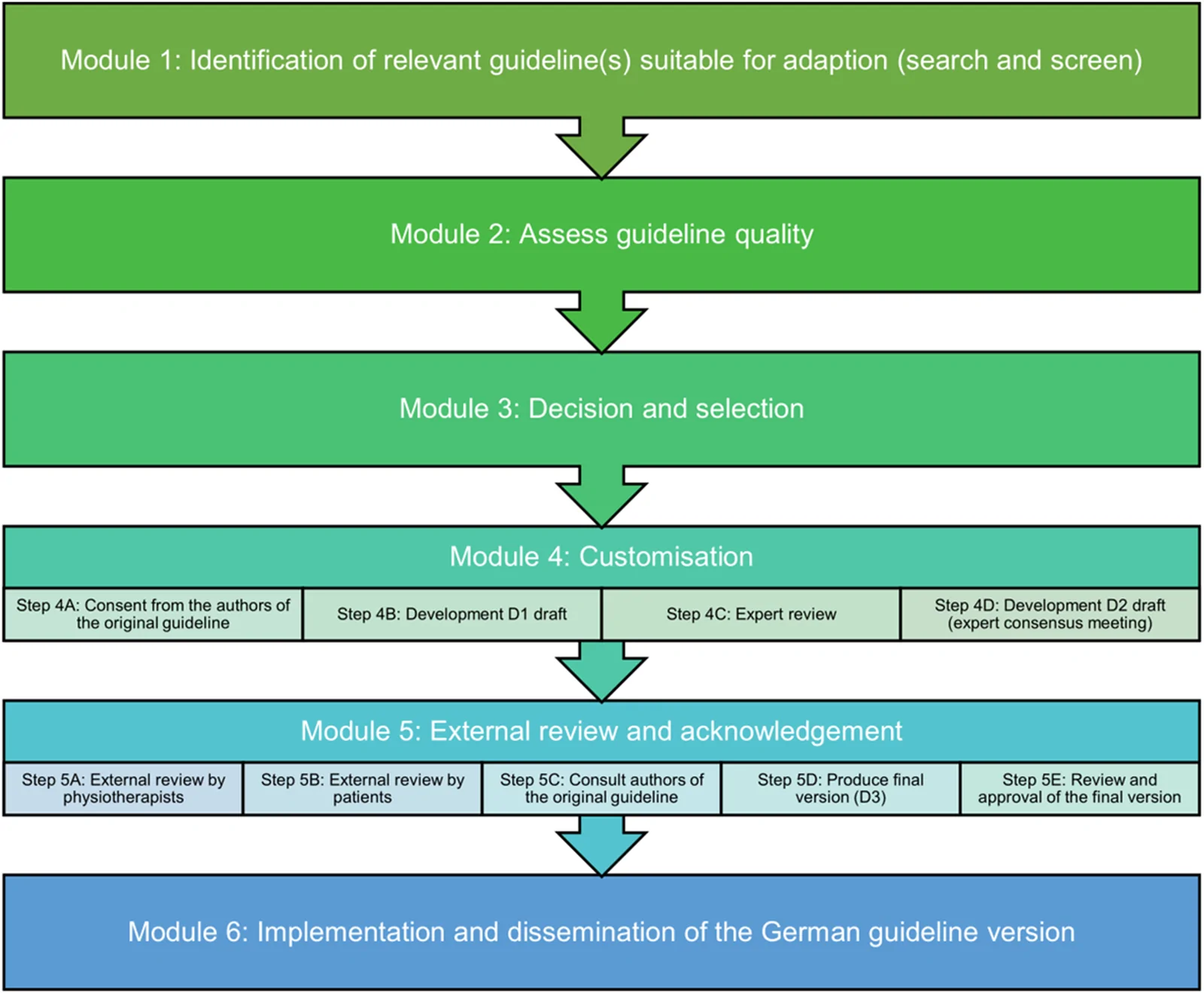
Study procedures (modified from the ADAPTE process and the recommendations from kirchner et al. (25, 26).

### Module 1: Identification of relevant guideline(s) suitable for adaptation (search and screen)

The aim of Module 1 was to identify the most suitable existing physiotherapy guideline(s) for managing patients with osteoporosis through structured, systematic and transparent methods. According to the ADOPTE recommendations, we completed the module 'Search and Screen' (25).

The literature search was informed by and is reported according to the PRISMA-2020 statement (28) and the recommendations by Kirchner et al. (26). We aimed to employ a sensitive search strategy by incorporating three scientific databases and nine international guideline databases, as well as relevant websites and registers.

#### Eligibility criteria

We included all documents which fulfilled the following inclusion criteria:.

1. Comprehensive professional guidelines designed explicitly and solely for physiotherapists/physiotherapy. To differentiate between guideline and non-guideline documents (29), we used the approach by Agostini et al. (21) and followed the definition provided by Lohr and Field: “Clinical practice guidelines are systematically developed statements to assist practitioners and patient decision about appropriate healthcare for specific clinical circumstances” (18). To classify a document as a guideline, we followed the approach applied by Agostini et al. (21): We did not distinguish between guidelines, consensus statements, or position papers, nor did we assess reports based on their use of evidence-based methods, as long as the primary goal of the report was to provide systematically developed recommendations.
2. Guidelines focusing solely on the physiotherapeutic management (e.g., assessment, prevention, treatment) of individuals with osteoporosis.
3. Guidelines published in German or English.
4. Current guidelines published within the last 5 years (01.01.2020–01.03.2025).
5. Guidelines with acceptable quality, indicated by an AGREE II rating (described below) of at least 50% in domain 3 (rigour of development) and the overall AGREE II rating, respectively (26).

#### Exclusion criteria

1. Single sections (focusing on rehabilitation/physiotherapy) of a comprehensive guideline, since we aimed to find and adapt a comprehensive physiotherapy guideline.
2. Multidisciplinary/interdisciplinary guidelines, since we aimed to adapt a physiotherapy guideline.
3. Guidelines on osteoporosis-related conditions or syndromes, such as frailty, fall risk, or hip fracture.

#### Information sources

We searched the following three medical databases:

- MEDLINE via PubMed
- PEDro (Physiotherapy Evidence Database)
- Web of Science

We searched the following nine sources/websites:

- Guidelines International Network (G-I-N) [https://g-i-n.net/international-guidelines-library]
- Trip Medical Database [https://www.tripdatabase.com]
- MAGICapp (A digital authoring and publication platform for the evidence ecosystem, by MAGIC Evidence Ecosystem Foundation) [https://app.magicapp.org]
- Institute for Quality and Efficiency in Health Care (Institut für Qualität und Wirtschaftlichkeit im Gesundheitswesen, IQWiG) [https://iqwig.de]
- American Physical Therapy Association (APTA) [https://www.apta.org/patient-care/evidence-based-practice-resources/cpgs]
- Academy of Orthopaedic Physical Therapy (AOPT) of the American Physical Therapy Association (APTA) [https://www.orthopt.org/content/practice/clinical-practice-guidelines]
- Royal Dutch Society for Physiotherapy (Koninklijk Nederlands Genootschap voor Fysiotherapie, KNGF) [https://www.kennisplatformfysiotherapie.nl/guidelines/]
- Chartered Society of Physiotherapy (CSP) [https://www.csp.org.uk/professional-clinical/professional-guidance/publications]
- World Physiotherapy, the global body for >120 physiotherapy member organisations [https://world.physio/resources/policies-guidelines]

Moreover, we did not search the websites of two other significant international physiotherapy associations (the Australian Physiotherapy Association and the Canadian Physiotherapy Association) because their guideline databases are only accessible to members.

We did not seek additional guidelines by searching study registers or contacting authors, experts, or other relevant individuals. However, we searched Google Scholar and manually screened the reference lists for all the included guidelines. In addition, we screened three current reviews of CPG for non-pharmacological and/or exercise recommendations for people with osteoporosis from the authors' archive for potentially eligible guidelines, namely the reviews by Agostini et al. 2024 (21), Arceo-Mendoza et al. 2021 (30), and Iolascon et al. 2021 (31).

#### Search strategy

The search strategy was designed by one author (TB) around the three concepts “osteoporosis”, “physiotherapy”, and “guidelines”. According to each database and website, keywords and/or controlled vocabulary thesaurus (e.g., Medical Subject Headings) were used (in combination) to identify relevant reports/guidelines. We limited the search to publications from 2020 to 2025 to identify the most current guideline(s). We did not apply any other limits, restrictions, or filters. The search was run on March 12, 2025; we did not update the search.

The search strategy for each information source/database is reported in the Supplementary material.

#### Selection process

Two reviewers (BS, TB) screened the titles and abstracts of each record independently against the inclusion and exclusion criteria by using the Catchii web application (32). Two reviewers screened the remaining reports independently at the full-text level (BS, TB). Conflicts at both levels were resolved by discussion. If two guidelines by the same organisation covering the same topics or with the same scope were found, we included the most recent one.

#### Data collection process

We extracted the following data from each included report/guideline: title, organisation, year of publication, first author, and country/region of origin using a standardised form. One reviewer (TB) performed data extraction, verified by a second reviewer (BS).

### Module 2: Quality assessment

According to the ADAPTE process and Kirchner et al. (25, 26), the general aim is to include methodologically high-quality guidelines for adaptation of guidelines. Thus, the methodological quality of relevant guidelines was assessed with the AGREE II instrument (33, 34). AGREE II is the most widely used and best-evaluated instrument internationally (35). Pairs of two reviewers (BS, TB) independently completed the AGREE II checklist for each relevant guideline. If a guideline was included and rated in the review by Agostini et al. 2024 (21), we used their AGREE II rating, but checked for accuracy.

The AGREE II appraisal checklist comprises 23 items, each rated on a 7-point scale (1 = strongly disagree to 7 = strongly agree). The items are divided into six domains: scope and purpose, stakeholder involvement, the rigour of development, clarity of presentation, applicability, and editorial independence. The final score for each guideline is the average of the results computed by each reviewer, as described in the AGREE II instrument directives (33, 34).

### Module 3: Decision and selection

Based on the ratings of the following four criteria, the research team decided which guideline(s) to select and prioritise for translation and adaptation (25, 26):

1. Guideline quality (AGREE II domain 3: “Rigour of development”) [scale: 0% to 100%].
2. Composition of the guideline group and the interests and conflicts of interest of the guideline authors (AGREE II domain 6: “Editorial independence” [0%–100%].
3. Guideline transferability to the conditions of physiotherapy in the German health system [mean of the ratings of two independent reviewers on a scale from 0%–100% (very low to very high transferability)].
4. Guideline currency (prioritising more current guidelines) [prioritising more current guidelines].

We finally identified two appropriate guidelines and adopted one. However, as described in the study protocol, we anticipated these two scenarios before the study started: (1) If the search process identifies a single guideline, we will ask for approval from the authors and/or organisations to translate and adapt this guideline. (2) If we identify two or more different guidelines and decide to adapt more than one guideline, the following procedures (modules 4, 5 and 6) will be repeated for each guideline.

### Module 4: Customisation

#### Step 4A: Consent from the authors of the original guideline

The principal investigator (TB) obtained approval from the authors of the original guideline. The request included approval to translate and adapt the original guideline into German and to publish a German version of the guideline. All study procedures were performed in consultation with and with the agreement of the authors of the original guideline. We informed the authors of the original guideline about all changes and adaptations to the guideline. According to the study protocol, the authors of the original guideline were acknowledged and/or listed as co-authors in all publications of the guideline's German version. The developers of the source guideline had no influence on the independent appraisal and adaptation processes.

#### Step 4B: Development of the first draft of the guideline (D1 draft version)

Members of the research team (MH, TB) selected relevant text/paragraphs from the original guideline for inclusion in the German version. A research team member (MH) prepared all relevant translation text.

The guideline was translated from English to German by two different artificial intelligence (AI) systems. We used “Perplexity AI”, a web search engine that utilises a large language model to process queries and synthesise responses based on web search results (https://www.perplexity.ai/; access date: March 11, 2025), and “DeepL Translator”, a neural machine translation service developed by DeepL SE (https://www.deepl.com/de/translator; API/Client library version; access date: March 13, 2025). This process resulted in two German-language versions of the guideline (AI1 and AI2). Two research team members (MH, TB) developed a synthesis (draft version D1) of these two AI-generated translations. In case of disagreement, two or more possible options (translations/wordings) were reported in a D1 draft report, which was used in the next step.

#### Step 4C: expert review

We built an expert committee consisting of the following stakeholders/persons:

- 2 clinical experts in the field of geriatric physiotherapy/physical rehabilitation with ≥ 2 years of clinical expertise in the management of patients with osteoporosis (JN, VH)
- 2 scientific/methodical experts in the field of clinical practice guidelines (development, adaptation, implementation) (SS, GD)

Every expert declared their conflict of interest before participation.

Each expert received the full D1 draft report, including (a) the original English version of the guideline, (b) the two AI-generated translations and (c) the synthesis (D1 draft). Each expert independently reviewed this D1 draft report by checking and commenting on the following criteria:

Criterion 1: Linguistic correctness/accuracy of the translation.
Criterion 2: Appropriateness of wording concerning physiotherapeutic/medical terminology.
Criterion 3: Appropriateness of the recommendations concerning the German healthcare system and the legal and organisational framework of physiotherapy care in Germany.
Criterion 4: Agreement of the APTA Geriatrics guideline recommendations with the recommendations in the most current 2023 version of the CPG for the “Prevention, diagnosis and therapy of osteoporosis in postmenopausal women and men over the age of 50′ published by the AWMF (7). This CPG is a multi-professional guideline focusing on the medical management of patients with osteoporosis and includes some rather general statements on physiotherapeutic management.

Each expert provided a written report, including feedback and comments on the D1 draft concerning the four criteria and other aspects relevant to the expert's judgment.

#### Step 4D: development of the second draft of the guideline (D2) by using an expert consensus meeting

One author (MH) collected and structured the feedback reports of the four experts. We then convened a virtual consensus meeting with all experts to develop the guideline's second draft (D2). We used the experts' comments on the D1 draft to produce the D2 draft. We aimed to reach a consensus on the D2 draft through discussion.

### Module 5: External review and acknowledgement

Steps 5A, 5B and 5C were performed simultaneously.

#### Step 5A: External review by target users (physiotherapists)

The D2 draft of the guideline was provided to a convenience sample of three clinical physiotherapists who had worked with patients with osteoporosis for at least one year. We asked the physiotherapists to evaluate the German guideline draft D2 for comprehensibility, readability and feasibility. Each physiotherapist provided a written report. Additionally, we conducted semi-structured interviews to gather in-depth feedback on the guideline. The interviews were conducted by one author (MH), took place either face-to-face or virtually, were recorded via audio or video, and were scheduled for 30 to 45 min. The responses were categorized by one researcher (MH), but not checked by another researcher.

Physiotherapists were recruited through personal contacts of the research team and the DGPTW (German Society for Physiotherapy Science, “Deutsche Gesellschaft für Physiotherapiewissenschaft”).

#### Step 5B: External review by patients with osteoporosis

We invited two patients with osteoporosis to review the “recommendations section” of the German guideline draft D2. We recruited patients from the research team's institutions/clinics. The inclusion criteria were: (a) age ≥ 50 years, (b) suspected or confirmed osteoporosis, (c) experience with physiotherapy concerning the osteoporosis diagnosis, and (d) ability to give written informed consent (e.g., free of cognitive impairment, no German language barriers). A priori, we decided to include at least one female individual, considering the higher prevalence of osteoporosis in women.

We provided the D2 draft of the guideline to the two patients, who were asked to provide feedback from their perspective. We conducted semi-structured interviews (scheduled for 30 to 60 min; interviewer: MH) to gather in-depth feedback.

#### Step 5C: Consult with the developers of the source guideline

We sent the list of adaptations arising from Module 4 to the authors of the original guideline. The authors were asked to comment on the adaptations and approve the D2 draft.

#### Step 5D: Produce final guidance document/guideline (D3 draft)

We reviewed all comments and feedback from the prior steps (5A to 5C). Two team members (MH, TB) produced a third draft (D3) based on feedback from the physiotherapists, patients, and the authors of the original guideline. This D3 draft was reviewed by the four experts (JN, VH, SS, GD) and revised accordingly by two team members (MH, TB).

#### Step 5E: Review and approval of the final version

The final version of the guideline was reviewed by the whole research team (MH, TB, JN, VH, SS, GD, BS).

### Module 6: Implementation and dissemination of the German guideline version

We developed initial approaches to facilitate the dissemination and implementation of the German guideline version, including a dissemination plan to reach clinicians and an educational workshop.

### Data analysis

We used only descriptive analysis of the data. All analyses were performed using SPSS version 23 and MS Excel.

The interview data (physiotherapists and patients) were analyzed using a descriptive approach. The interviews were recorded, but not transcribed verbatim. Relevant statements were identified and organized into categories based on recurring topics and patterns. The analysis focused on summarizing participants' responses without applying interpretative or theoretical frameworks. Categories were developed inductively from the data. The aim of this approach was to provide a structured and transparent overview of the reported experiences and viewpoints to improve comprehensibility, readability and feasibility of the German guideline version.

## Results

All procedures were performed in accordance with the study protocol, without any deviations.

### Module 1: Identification of relevant guideline(s)

The literature search process is described in the flow chart (Figure 2). Detailed results for each database/source are reported in the Supplementary material. The references of the excluded records at full-text level (with reasons for exclusion) are reported in the Supplementary material.

**Figure 2:**
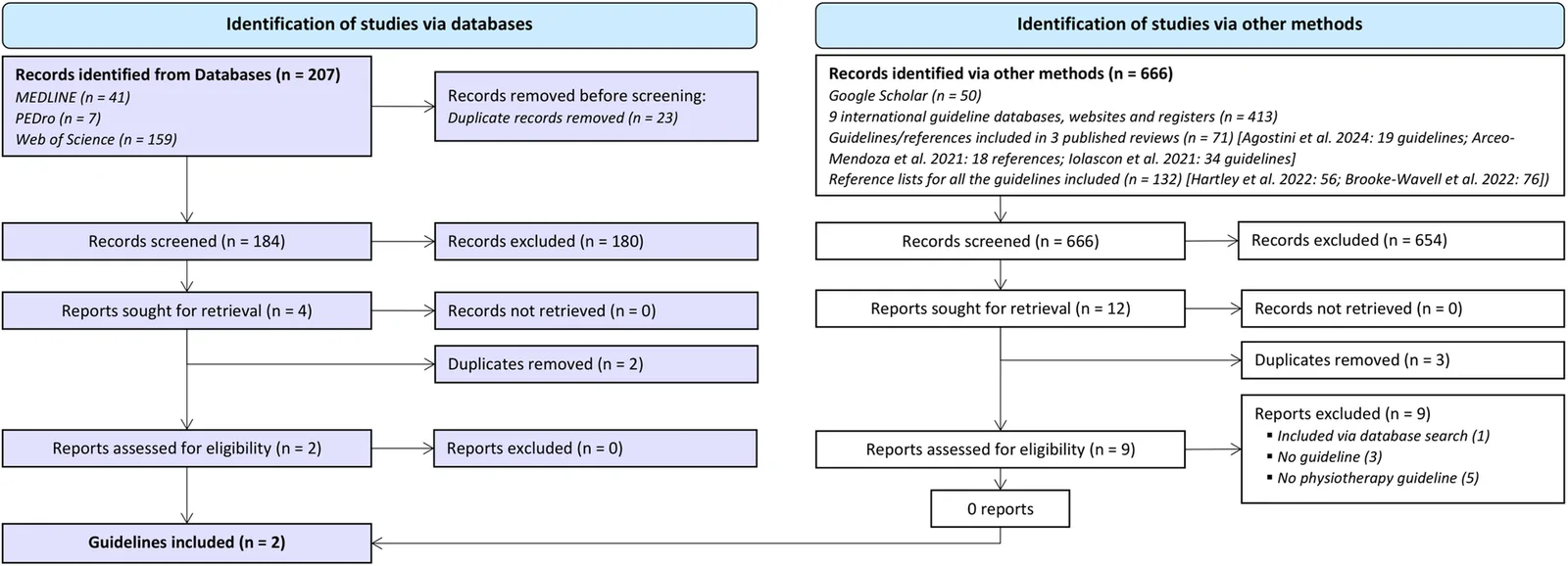
Flow chart

We screened 207 records identified from 3 databases and 666 records identified via other methods, respectively. Finally, two guidelines were identified as suitable for adaptation: (1) Guideline from the “APTA Geriatrics” (American Physical Therapy Association Academy of Geriatric Physical Therapy, USA), authored by Hartley et al. 2022 and titled “Physical Therapist Management of Patients With Suspected or Confirmed Osteoporosis: A Clinical Practice Guideline From the Academy of Geriatric Physical Therapy” (36). (2) Guideline from the UK authored by Brooke-Wavell et al. 2022 ('Strong, steady and straight: UK consensus statement on physical activity and exercise for osteoporosis”) (37).

### Module 2: Quality assessment

The AGREE II ratings for both guidelines (USA and UK) are reported in the Supplementary material. The AGREE II total score was 50% for the guideline by Hartley et al. (36) and 58% for the guideline by Brooke-Wavell et al. 2022 (37).

### Module 3: Decision and selection

Results of the criteria ratings are reported in the Supplementary material. According to our predefined criteria, we decided to adapt the APTA Geriatrics guideline from the US, published by Hartley et al. 2022 (36) due to the better AGREE II ratings in the domains 3 and 6 (guideline quality/rigour of development and editorial independence, respectively). Notably, transferability was rated as moderate for both guidelines, primarily due to challenges arising from the structure of the German healthcare system, the limited autonomy of German physiotherapists, their level of education and qualifications, and the prescription and billing options for individual measures.

### Module 4: Customisation

#### Step 4A: Consent from the authors of the original guideline

The principal investigator (TB) obtained approval from the authors of the original APTA Geriatrics guideline (GW Hartley and KG Avin).

#### Step 4B: Development of the first draft of the guideline (D1 version)

We selected the relevant text/paragraphs from the original English APTA Geriatrics Guideline for inclusion in the German version. The “basic document” was the guideline report/article by Hartley et al. 2022 published in the Journal of Geriatric Physical Therapy (36).

The guideline was translated from English into German by two AI systems (Perplexity AI and DeepL Translator). We created a table that included the original English guideline text, the two AI translations, and the synthesis (draft 1, developed by two research team members). We highlighted any text/translations (e.g., words, wordings and sentences) rated as “critical/unclear”.

During the development of the D1 draft, we decided to create a glossary for the German version of the guideline, which included key terms and their corresponding definitions used throughout the guideline.

#### Step 4C: Expert review

All experts declared no conflicts of interest regarding the adaptation of the APTA Geriatrics guideline. Each member of the expert committee received the D1 draft report, including the original English version of the guideline, (b) the two AI-generated translations, (c) the synthesis (D1 draft) and (d) a draft of the glossary. Each expert provided feedback on these documents in the form of a written report.

#### Step 4D: development of the second draft of the guideline (D2) by using an expert consensus meeting

The experts' feedback was used to develop a further draft of the guideline (D2 draft; MH and TB). The virtual expert consensus meeting (video call via Microsoft Teams) was held on April 14, 2025. It included the two clinical experts (JN, VH), the two scientific/methodical experts (SS, GD), and two other members of the research team (MH, TB). We reviewed the D2 draft and resolved any disagreements and unclear wording through discussion. All changes were documented in a written report.

We reached consensus on the D2 draft through discussion. The most significant changes to the original guideline were:

1. We included a section “Notes on the German version of the guideline” (English translation in the Supplementary material) because we considered such a section to be useful for readers of the German guideline to better understand the context and the applicability of the guideline.
2. We included a glossary, including the following terms, because we considered such a glossary to be helpful for readers of the German guideline to understand the guideline recommendations better:
  - low-trauma fracture (*Niedrig-Trauma-Fraktur*)
  - physical activity (*Bewegung*)
  - physical inactivity (*Bewegungsmangel*)
  - exercise/exercise training (*Training*)
  - weight-bearing exercise *(gewichtstragendes Training)*
  - static weight-bearing exercise *(statisches, gewichtstragendes Training)*
  - dynamic weight-bearing exercise with low force *(dynamisches, gewichtstragendes Training mit geringer Krafteinwirkung)*
  - dynamic weight-bearing exercise with high force *(dynamisches, gewichtstragendes Training mit hoher Krafteinwirkung)*
  - non-weight-bearing exercise low force *(nicht gewichtstragendes Training mit geringer Krafteinwirkung)*
  - non-weight-bearing exercise high force *(nicht gewichtstragendes Training mit hoher Krafteinwirkung)*
  - exercise types (*Trainingsformen*)
  - high-impact training *(“high-impact” Training)*
  - odd-impact training *(„odd-impact‘ Training)*
3. We used different colours for each “exercise type” (e.g., ’static weight-bearing exercise' was written in blue) to better differentiate the exercise types and to make it easier for readers of the German guideline to understand the guideline recommendations.
4. The APTA Geriatrics guideline recommends that clinicians managing patients with osteoporosis consult the APTA Geriatrics clinical guidance statement directed at fall risk reduction, since guidance on fall risk assessment is not addressed in the guideline. We added a context-specific note on the availability of guidelines for fall risk assessment and management: “For care provision in German-speaking regions, recommendations on physical training for fall prevention among older people living at home are available, issued by the Federal Initiative for Fall Prevention.”
5. (38, 39) Chapter “Implementation”: The original English version of the guideline contains information on the implementation of the guideline in the USA. Some of this information may not apply to the German context. Therefore, this chapter of the guideline was not translated. We included a note on this decision in the German version of the guideline.
6. The original guideline is intended to be used in the USA. We aimed to translate and adapt the guideline for use in the German healthcare system. However, we think that physiotherapists and healthcare professionals in other German-speaking countries can also use the guideline. Thus, we used the wording “Germany, Austria and Switzerland” in some instances.
7. We added “Information on the German adapted version of the guideline” at the end of the guideline document, including a statement on the authors’ contributions, acknowledgements, a conflict-of-interest statement, an open access statement, a copyright statement and contact information of the corresponding author.

In addition, the following decisions were made regarding the language and content of the guideline:

1. Person-first language was used throughout.
2. A gender-conscious language was chosen to express equal treatment of all genders and identities.
3. Some English terms that are commonly also used in German (e.g., high-impact training) were not translated into German and written in italics.
4. Other aspects addressed during the expert conference were of an orthographic and grammatical nature or concerned punctuation.

After the consensus meeting, the principal investigator (TB) created a usable German version of the guideline with a user-friendly layout (PDF document) that was, however, based on the design and layout of the original English guideline article (36).

### Module 5: External review and acknowledgement

Steps 5A, 5B and 5C were performed simultaneously.

#### Step 5A: External review by target users (physiotherapists)

Three clinical physiotherapists reviewed the D2 draft of the guideline to assess its comprehensibility, readability, and feasibility. Characteristics of the physiotherapists are reported in the Supplementary material. Briefly, the physiotherapists (two females and one male) were between 26 and 34 years old, treated 2 to 50 patients with osteoporosis per month (on average), and were not aware of any clinical practice guidelines for osteoporosis.

The interviews were conducted in April and May 2025 and lasted between 14 and 47 min. The interviews revealed several recommendations and ideas that were used to modify the D2 and create the D3 draft (a complete list of comments and suggestions is included in the Supplementary material). This includes:

1. One participant recommended including a definition of “impact training” in the glossary. We added such a definition.
2. Two participants recommended using different, more contrasting colours for the various exercise types and to avoid green and red colours. We changed the colours used for the exercise types to increase accessibility for visually impaired readers.
3. One participant mentioned that the “we” term (as used in the English version for the Original APTA-guideline-team) is misleading. We improved the document's comprehensibility by avoiding the use of the term “we”. Instead, we used neutral wordings or the term “guideline team” (German: “APTA-Leitlinien-Team”) as used by Hartley et al. in several other instances.

#### Step 5B: External review by patients with osteoporosis

The interviews with the two postmenopausal female patients with osteoporosis (80 and 75 years) were conducted in April 2025. Osteoporosis had been diagnosed 15 and 1 years ago, respectively. The interviews lasted 31 and 16 min, respectively. Details of the patients' characteristics and their experience with exercise training are provided in the Supplementary material.

Both patients considered the guideline recommendations for postmenopausal women feasible. However, the patients considered some of the exercise recommendations as not feasible for their individual application due to their current health restrictions (e.g., progressive-resistance strength training, jogging; detailed results in the Supplementary material). We consider these comments to be individual, person-specific limitations to the guideline's practicality. Because patients generally rated the guidelines as practical, and physiotherapists can make individual adjustments for specific individuals, no changes were made to the German guideline document based on the patients’ feedback.

#### Step 5C: Consult with developers of the source guideline

A draft of the German guideline version (D2) was sent to the developers of the source guideline (GH, KA), along with a list and justification of significant changes to the original guideline (as described above). The developers approved all changes to this version of the guideline.

#### Step 5D: Produce final guidance document/guideline (D3 draft)

We reviewed all comments and feedback from the prior steps and produced a third draft (D3) based on input from physiotherapists, patients, and the authors of the original guideline. This D3 draft was reviewed by the four experts and revised accordingly by two team members (MH, TB). We made the following minor changes to the D3 draft to produce the final version of the guideline:

1. Some wording has been slightly adjusted to improve clarity, but without significantly changing the content of the statement. E.g., the wording “Grade B, recommendation based on moderate evidence” was changed to “Grade B, moderate quality of evidence” (German: “Empfehlungsgrad B, Empfehlung basiert auf moderater Qualität der Evidenz” changed to “Empfehlungsgrad B, moderate Qualität der Evidenz”).
2. We added a statement to the “Information on the German adapted version of the guideline” to provide information on the methodological procedures of the translation and adaptation of the German guideline version.
3. We corrected some typos.

#### Step 5E: Review and approval of the final version

The final version of the guideline was reviewed and approved by the whole research team (MH, TB, JN, VH, SS, GD, BS) and the developers of the source guideline (GH, KA). Only a few minor, non-substantive changes were suggested and adopted. A table containing the original English text and the respective final German translation is provided in the Supplementary material.

### Module 6: Implementation and dissemination of the German guideline version

We developed and initiated the following methods to facilitate the dissemination of the German guideline version: (1) Open access publication of the German guideline in the Open Science Framework repository (https://osf.io/yav94/files/s357b). (2) Announcement of the guideline within national physiotherapy associations. (3) Writing an accompanying article for a clinically oriented physiotherapy journal (40).

To implement the guideline in clinical institutions, we developed an educational course for physiotherapists. The course has already been conducted in two German physiotherapy clinics. The course evaluation has shown promising results, with high participant acceptance (40).

## Discussion

This paper describes the systematic identification, translation and contextual adaptation of the APTA Geriatrics clinical practice guideline for the physiotherapy management of patients with osteoporosis into German. The aim of this process was to provide physiotherapists in German-speaking countries with an evidence-based, linguistically accurate, and contextually appropriate guideline to support clinical decision-making and improve the quality of care for individuals with osteoporosis.

We followed established frameworks for guideline translation and adaptation to ensure methodological rigour and transparency (25–27). After several rounds of review and feedback, the final German version of the guideline includes a comprehensive glossary, and the “exercise types” are presented in different colours. These modifications should help readers better understand and apply the guideline recommendations.

The user feedback highlighted both the perceived usefulness of the guideline and areas needing additional clarification, such as the glossary. This suggests that future translation efforts should prioritize not only linguistic accuracy but also terminological transparency and usability for end users. In addition, these findings underline the importance of involving target users early and systematically in guideline translation projects, as others have reported (41).

At the time of study design and start, there was no current specific physiotherapy guideline for the management of patients with osteoporosis in Germany and German-speaking countries (21). Physiotherapists could find recommendations in the interdisciplinary 2023 German guideline on the prevention, diagnosis and therapy of osteoporosis (Dachverband Osteologie 2023), which includes only general statements on the physiotherapeutic management of patients with osteoporosis. However, in the meantime, the German-language DVO guideline on “physical exercise for fracture prevention” was published (42–45). This guideline addresses various objectives in the fields of physical training and fracture prevention among middle-aged and older adults. The guideline is intended for healthcare professionals working in the field of osteoporosis and fracture prevention, such as physiotherapists, exercise therapists, sports scientists and exercise instructors (42). Although this guideline includes up-to-date evidence- and consensus-based practice recommendations for effective, safe, and transferable training programs for fracture prevention, the guideline is not designed as a physiotherapy guideline (43). The DVO guideline and the APTA Geriatrics guideline agree in central recommendations on physical exercise, including that physical exercise is recommended as a key intervention for the prevention of fractures and the treatment of osteoporosis, that resistance training should be an essential component of the training program and that fall prevention through balance and functional training is an essential part of fracture prevention management. A comparison of the two guidelines reveals no fundamental contradiction, though there are differences in emphasis and specificity. The DVO guideline focuses more on *what is effective*, whereas the APTA Geriatrics guideline places greater emphasis on *identifying the target population and the safety conditions* under which such loading is appropriate. For instance, the more recent DVO guideline (42) clearly recommends high-intensity strength training (where individually feasible) as well as impact-based loading (e.g., jumping, hopping, rapid stair climbing) (42), whereas the APTA Geriatrics guideline is more cautious in this regard, placing greater weight on individual risk stratification and tailoring the approach to the patient's specific fracture risk (36). Physiotherapists in German-speaking countries can now use two osteoporosis guidelines for therapeutic care: the adapted physiotherapy-specific APTA Geriatrics guideline, with broader physiotherapy-specific recommendations on the management of patients with suspected or confirmed osteoporosis (Hartley et al. 2022), and the DVO guideline, which focuses on physical exercise for fracture prevention (42).

### Limitations

The first limitation of this study is that the literature search was conducted in March 2025 and has not been updated. Thus, we might have missed a recently published guideline. However, we performed a broad and sensitive literature search to identify appropriate source guidelines (e.g., 3 databases and 9 international guideline databases) and we are not aware of any recently published physiotherapy-specific guideline for osteoporosis.

The second limitation is that we included only guidelines focused on the physiotherapeutic management of individuals with osteoporosis, in line with this project's aim. However, the clear distinction between professional and interprofessional guidelines might not always be so clear or strict. Thus, we might have missed some (interprofessional) guidelines that provide comprehensive and explicit recommendations on the physiotherapy management of osteoporosis as part of a “broader” guideline.

The third limitation is that although the expert panel and user group were diverse, they may not fully represent all physiotherapy settings or regions where the guideline will be used. The German draft of the guideline was reviewed by three physiotherapists only (the target users). A larger group of users would have increased the likelihood of detecting inconsistencies and potential issues concerning comprehensibility, readability and feasibility. Moreover, the physiotherapists' feedback was based exclusively on the “theoretical” review of the document, since the participants did not apply the guideline in practice. However, we assume that the relatively diverse sample of participants (in terms of age, sex, and education) identified the major problems with the comprehensibility and readability of the German guideline.

The fourth limitation is that we did not translate and adopt the guideline chapter “implementation,” which covers the guideline's implementation in the USA and largely does not apply to the German context. Due to limited resources, we did not formulate suggestions on guideline implementation into the German healthcare context. However, implementation and dissemination of the German guideline version have been initiated as we already announced the guideline via national physiotherapy associations, published an article in a national physiotherapy journal and developed an educational course for physiotherapists (40). Future research should explore barriers and facilitators to implementation and assess the real-world effectiveness of the adapted guideline in improving care for individuals with osteoporosis.

The fifth limitation is that we did not follow all steps as recommended by the ADAPTE collaboration (25). For example, we did not determine health questions because we aimed to adapt an existing, comprehensive physiotherapy guideline. Moreover, we did not assess the currency, validity, and context-specific applicability of the recommendations presented in the original APTA Geriatrics guideline. For the process of guideline adaptation, Kirchner et al. (Kirchner et al. 2020) and Kristiansen et al. (Kristiansen et al. 2014) recommend that for each recommendation, one assesses whether it can stand as is or requires modification, exclusion, or development of new recommendations. In the event of a planned exclusion or modification of a recommendation, the available evidence must be re-evaluated, potentially in consultation with the authors of the original guideline (46). This re-evaluation of the evidence base should be guided by the established standards of the international GRADE group (47). Since this research project was performed without any funding, we were forced to forgo these elaborate and time-consuming processes.

A sixth limitation concerns the source guideline's inherent limitations. The APTA Geriatrics guideline itself is an adapted guideline, based on a systematic review of existing clinical practice guidelines and following the ADAPTE framework (36). Thus, the recommendations contained in this guideline were derived from the 2021 Scottish Intercollegiate Guideline Network (SIGN) document on the “management of osteoporosis and the prevention of fragility fractures” (48). The SIGN guideline used evidence published between 2003 and 2018 and thus did not include the latest evidence. The APTA Geriatrics guideline faces limitations due to its reliance on the quality of source guidelines and the potential for reduced generalizability of U.S. healthcare models to international contexts. Further limitations include the temporal lag in integrating the latest scientific evidence, the necessary reliance on expert consensus over high-quality RCT evidence [given the lack of evidence on physiotherapy management of individuals with osteoporosis (36, 49)], and the clinical heterogeneity resulting from bridging suspected and confirmed osteoporosis in patient stratification. The APTA Geriatrics guideline does not address strategies to assess the risk of falls in individuals with osteoporosis, which is, however, highly important for physical therapists in clinical care (50). We added a context-specific note on the availability of German guidelines for fall risk assessment and management to the German guideline version.

A seventh limitation is that we performed a forward translation with two AI systems and a consensus-based synthesis, but without independent human forward translation and no formal back-translation.

Despite these limitations, several indicators suggest that the current German guideline version closely aligns with the original English document (“content validity”) and that the risk of translation errors is low. For example, we followed the ADAPTE framework (25) and the methods paper for adapting international physiotherapy guidelines of the German Society for Physiotherapy Science (26) to a very large extent. In addition, two AI systems (based on large language models) were used for the translations. There is initial evidence that large language models can produce high-quality translations of scientific documents (51). Furthermore, the guideline drafts were thoroughly reviewed by an experienced, sufficiently large expert committee, three physiotherapists, and two older individuals with osteoporosis.

### Implications

This work has several implications. First, it demonstrates the feasibility of systematically translating and adapting an APTA Geriatrics guideline for use in another language and health care system. To our knowledge, this is also the first project that used the methods paper for adapting international physiotherapy guidelines into German (26). This guidance was useful and feasible, although we could not follow all recommended steps. The structured process we employed may serve as a model for future translation and adaptation of other physiotherapy guidelines into German.

Second, this study underscores that a mere linguistic translation (e.g., by modern translation systems such as large language models) is insufficient; careful cultural, professional, and system-level adaptation is necessary to ensure that recommendations are both understandable and implementable.

The third implication concerns the clinical physiotherapeutic management of people with osteoporosis. Given the impressive and increasing prevalence rates of osteoporosis in Germany (more than 4.5 million women and 1.2 million men affected) (2), the lack of current national evidence-based physiotherapy guidelines for osteoporosis was striking. Now, the German version of the APTA Geriatrics guideline provides physiotherapists with high-quality, evidence-based recommendations on exercise interventions that impact bone mineral density in individuals with suspected or confirmed osteoporosis. The DVO guideline provides additional recommendations on physical exercise for fracture prevention (44).

The last module of our study addresses the implementation and dissemination of the German guideline version. Open-access publication of the guideline document on the OSF (https://osf.io/yav94/files/s357b), publication of an accompanying article in a clinically oriented physiotherapy journal (Hahn und Braun 2026), and development of an educational course for physiotherapists (Hahn und Braun 2026) are the first completed steps to facilitate guideline implementation and dissemination. Further announcements via social media, national physiotherapy associations, professional journals, and specialist conferences will further contribute to the awareness of the guideline.

Further research should address the guideline's applicability across all German-speaking countries (including Austria and Switzerland), content validity, implementation success, and potential improvement in patient care.

## Conclusion

In conclusion, the German translation and adaptation of the APTA Geriatrics guideline on the management of patients with suspected or confirmed osteoporosis were conducted through a structured, participatory process that respected the original evidence base while integrating national contextual factors. This adapted guideline provides a foundation for evidence-based physiotherapy management of osteoporosis in Germany.

## Data Availability

The original contributions presented in the study are included in the article/Supplementary Material, further inquiries can be directed to the corresponding author/s.
