# Supplementary material for: Physical therapist management of patients with suspected or confirmed osteoporosis – German translation and contextual adaptation of a clinical practice guideline from the Academy of Geriatric Physical Therapy (USA)
Source: Front Rehabil Sci. 2026 Oct 12;7:1933821. doi: 10.3389/fresc.2026.1933821 (PMC13698835; doi:10.3389/fresc.2026.1933821)
Supplement: Supplementary file 1 [file Datasheet1.pdf]

Supplementary material for the article by Braun et al. “Physical therapist management of patients with suspected or confirmed osteoporosis – German adaption of a clinical practice guideline from the Academy of Geriatric Physical Therapy (USA)”

Content

Supplementary material 1: Search strategy for each information source/database ... 2

Supplementary material 2: Detailed results of the literature search, according to each database/source ... 4

Supplementary material 3: Additional sources – records sought for retrieval ... 5

Supplementary material 4: AGREE II ratings of the guidelines ... 7

Supplementary material 5: Assessment of guideline adaptability ... 8

Supplementary material 6: Notes on the German version of the guideline added to the guideline ... 9

Supplementary material 7: Characteristics of the physiotherapists (n = 3; target users) ... 11

Supplementary material 8: Physiotherapists’ comments and recommendations on the D2 draft ... 12

Supplementary material 9: Characteristics of the patients with osteoporosis (n = 2) ... 15

Supplementary material 10: Patients’ evaluation of guideline-feasibility (D2 draft) ... 16

Supplementary material 11: Original guideline text and final German translations ... 17

Supplementary material 1: Search strategy for each information source/database

Full search strategies for each database and information source		
**Information source/database**	**Search strategy (syntax)**	**Limits/internal filter(s)**
MEDLINE via PubMed	((modalities, physical therapy[MeSH Terms]) OR (physiotherapy) OR (physical therapy)) AND<br>((osteoporosis[MeSH Terms]) OR (osteoporosis)) AND<br>((clinical practice guideline[MeSH Terms]) OR (guideline))	Publication date: 2020 - present
PEDro (Physiotherapy Evidence Database)	osteoporosis	Method: practice guideline<br>Published Since: 2020
Web of Science	((ALL=(osteoporosis)) AND ALL=(physiotherapy OR (physical therapy))) AND ALL=(guidelines)	Publication date: 2020-01-01 to 2025-03-01
Guidelines International Network (G-I-N)	osteoporosis	Publication Year: 2020 - 2025
Trip Medical Database	osteoporosis	Year range: 2020 – 2025<br>Filter “Guidelines”
MAGICapp	osteoporosis	

Institute for Quality and Efficiency in Health Care (IQWiG)	Osteoporose	Ressort/Bereich: “Versorgung und Leitlinien“ (healthcare and guidelines“)
American Physical Therapy Association (APTA)	osteoporosis	
Academy of Orthopaedic Physical Therapy (AOPT) of the American Physical Therapy Association (APTA)	Not applicable: manual screening of the list of published guidelines published between 2020 and 2025	
Koninklijk Nederlands Genootschap voor Fysiotherapie (KNGF)	Not applicable: manual screening of the list of published guidelines	
Chartered Society of Physiotherapy (CSP)	osteoporosis	
World Physiotherapy	osteoporosis	Policy Topic: Guideline
GoogleScholar	physiotherapy osteoporosis guideline	Publication date: 2020 – present<br>First 50 records

Supplementary material 2: Detailed results of the literature search, according to each database/source

**MEDLINE via Pubmed (March 12, 2025)**		
#1	(modalities, physical therapy[MeSH Terms]) OR (physiotherapy) OR (physical therapy)	**490,998**
#2	(osteoporosis[MeSH Terms]) OR (osteoporosis)	**113,888**
#3	(clinical practice guideline[MeSH Terms]) OR (guideline)	**303,212**
#4	#1 AND #2 AND 3	**157**
#5	Internal filter: 2020 - present	**41**

**PEDro (March 12, 2025)**		
#1	Osteoporosis (Method: practice guideline; Published Since: 2020)	**7**

**Web of science (March 12, 2025)**		
#1	ALL=(osteoporosis) AND ALL=(physiotherapy OR (physical therapy)) AND ALL=(guideline)	**328**
#2	Internal filter: Publication date: 2020-01-01 to 2025-03-01	**159**

Supplementary material 3: Additional sources – records sought for retrieval

Additional Source	Records screened (n)	Records sought for retrieval in full text (n)	Records sought for retrieval in full text (reference)	Reason for exclusion
Google Scholar	50	5	Hartley et al. 2021: Physical Therapist Management of Patients With Suspected or Confirmed Osteoporosis: A Clinical Practice Guideline From the Academy of Geriatric Physical Therapy, doi: 10.1519/JPT.0000000000000357	Included via database search
			Brooke-Wavell et al. 2022: Strong, steady and straight: UK consensus statement on physical activity and exercise for osteoporosis: doi: 10.1136/bjsports-2021-104634	No professional physiotherapy guideline
			Beck et al. 2024: Exercise prescription to support the management of osteoporosis: an expert statement for exercise physiologists, doi: 10.31189/2165-7629-13-s2.497	Not a guideline (Abstract)
			Giangregorio et al. 2021: Exercise and other physical therapy interventions in the management of osteoporosis, doi: 10.1016/B978-0-12-813073-5.00069-1	Not a guideline (Book chapter)
			Nava 2020: Osteoporosis: prevention and personalized physiotherapy, doi: 10.4081/br.2020.36	Not a guideline (Narrative review)
Guidelines International Network (G-I-N)	3	1	Hartley et al. 2021: Physical Therapist Management of Patients With Suspected or Confirmed Osteoporosis: A Clinical Practice Guideline From the Academy of Geriatric Physical Therapy, doi: 10.1519/JPT.0000000000000357	Included via database search (DUBLICATE)
Trip Medical Database	365	0	---	---
MAGICapp	0	0	---	---
Institute for Quality and Efficiency in Health Care (IQWiG)	8	0	---	
American Physical Therapy Association (APTA)	5	1	Hartley et al. 2021: Physical Therapist Management of Patients With Suspected or Confirmed Osteoporosis: A Clinical Practice Guideline From the Academy of Geriatric Physical Therapy, doi: 10.1519/JPT.0000000000000357	Included via database search (DUBLICATE)
Academy of Orthopaedic Physical Therapy (AOPT) of	12	0	---	---

Additional Source	Records screened (n)	Records sought for retrieval in full text (n)	Records sought for retrieval in full text (reference)	Reason for exclusion
the American Physical Therapy Association (APTA)				
Koninklijk Nederlands Genootschap voor Fysiotherapie (KNGF)	17	0	---	---
Chartered Society of Physiotherapy (CSP)	0	0	---	---
World Physiotherapy	3	0	---	---
Review: Agostini et al. 2024	19	5	Bae et al. 2021: Position statement: exercise guidelines for osteoporosis management and fall prevention in osteoporosis patients. doi: 10.11005/jbm.2023.30.2.149.	No professional physiotherapy guideline
			Hartley et al. 2021: Physical Therapist Management of Patients With Suspected or Confirmed Osteoporosis: A Clinical Practice Guideline From the Academy of Geriatric Physical Therapy, doi: 10.1519/JPT.0000000000000357	Included via database search (DUBLICATE)
			LeBoff et al. 2022: The clinician's guide to prevention and treatment of osteoporosis. doi: 10.1007/s00198-021-05900-y.	No professional physiotherapy guideline
			Tai et al. 2023: Clinical practice guidelines for the prevention and treatment of osteoporosis in Taiwan: 2022 update. doi: 10.1016/j.jfma.2023.01.007.	No professional physiotherapy guideline
			Hoke et al. 2020: Impact of exercise on bone mineral density, fall prevention, and vertebral fragility fractures in postmenopausal osteoporotic women. doi: 10.1016/j.jocn.2020.04.040.	No professional physiotherapy guideline
Review: Arceo-Mendoza et al. 2021	18	0	---	---
Review: Iolascon et al. 2021	34	0	---	---
Included Guideline: Hartley et al. 2022	56	0	---	---
Brooke-Wavell et al. 2022	76	0	---	---
	666	12		

Supplementary material 4: AGREE II ratings of the guidelines

Guideline	Domain 1 (%)	Domain 2 (%)	Domain 3 (%)	Domain 4 (%)	Domain 5 (%)	Domain 6 (%)	Overall assessment (%)
Hartley et al. 2022	70%	50%	63%	80%	32%	81%	50%
Brooke-Wavell et al. 2022	81%	89%	34%	92%	56%	75%	58%
AGREE II ratings for the guideline by Hartley et al. 2022 taken from Agostini et al. 2024.							

Supplementary material 5: Assessment of guideline adaptability

**Criteria**	**Guideline by Hartley et al. 2022**	**Guideline by Brooke-Wavell et al. 2022**
1. Guideline quality (AGREE II domain 3: “Rigour of development”; 0 – 100 %)	63%	34%
2. Composition of the guideline group and the interests and conflicts of interest of the guideline authors (AGREE II domain 6: “Editorial independence”; 0 – 100 %)	81%	75%
3. Guideline transferability (0 – 100 %)	65%	75%
4. Guideline currency	Literature search: Jan 2018<br>Published: Mar 2022	Literature search: Sep 2017<br>Published: May 2022
AGREE II ratings for the guideline by Hartley et al. 2022 taken from Agostini et al. 2024		

Supplementary material 6: Notes on the German version of the guideline added to the guideline

**German version**	**English translation**
Hinweise zur deutschen Version der Leitlinie	Notes on the German version of the guideline
Diese Leitlinie ist eine übersetzte und adaptierte Version der US-Amerikanischen Leitlinie der „APTA Geriatrics“ (American Physical Therapy Association Academy of Geriatric Physical Therapy), also der „Sektion Geriatrie“ des US-Amerikanischen Berufsverbands für Physiotherapie.	This guideline is a translated and adapted version of the US guideline of the “APTA Geriatrics” (American Physical Therapy Association Academy of Geriatric Physical Therapy), the “Geriatrics Section” of the US American Professional Association for Physical Therapy.
Die Leitlinie wurde von Hartley et al. im Jahr 2022 in der Zeitschrift „Journal of Geriatric Physical Therapy“ publiziert (https://www.doi.org/10.1519/JPT.0000000000000346).	The guideline was published by Hartley et al. in 2022 in the journal “Journal of Geriatric Physical Therapy” (https://www.doi.org/10.1519/JPT.0000000000000346).
Der methodische Prozess der Entwicklung der deutschen Version der Leitlinie ist im Studienprotokoll beschrieben, das im Open Science Framework registriert wurde (https://doi.org/10.17605/OSF.IO/TYXGF).	The methodological process of developing the German version of the guideline is described in the study protocol, which was registered in the Open Science Framework (https://doi.org/10.17605/OSF.IO/TYXGF).
Die Original-Leitlinie wurde für Physiotherapeut:innen entwickelt, die Patient:innen mit vermuteter oder bestätigter Osteoporose in den USA bzw. im Kontext des amerikanischen Gesundheitssystems versorgen. Physiotherapeut:innen, die diese Leitlinie nutzen, können die Leitlinien-Empfehlungen auf die physiotherapeutische Versorgung in Deutschland, Österreich und der Schweiz übertragen, auch wenn in der Leitlinie entsprechende Formulierungen bezogen auf die USA enthalten sind.	The original guideline was developed for physical therapists who care for patients with suspected or confirmed osteoporosis in the United States or within the context of the American healthcare system. Physical therapists who use this guideline can apply the guideline recommendations to physical therapy care in Germany, Austria, and Switzerland, even if the guideline contains corresponding wording related to the United States.

Grundsätzlich dient diese Leitlinie als Entscheidungshilfe. Die Eigenschaften, Vorstellungen und Werte der individuellen Patient:innen, die individuelle Erfahrung der jeweiligen Physiotherapeut:innen (interne Evidenz) sowie die nationalen und regionalen Besonderheiten der jeweiligen Gesundheitssysteme (Deutschland, Österreich, Schweiz) sollten stets berücksichtigt werden.	This guideline is intended as a decision-making aid. The characteristics, beliefs, and values of the individual patient, the individual experience of the respective physiotherapists (internal evidence), and the national and regional characteristics of the respective healthcare systems (Germany, Austria, Switzerland) should always be taken into account.

Supplementary material 7: Characteristics of the physiotherapists (n = 3; target users)

Participant	Physiotherapist A	Physiotherapist B	Physiotherapist C
**Sex**	Male	Female	Female
**Age**	33 years	26 years	34 years
**Working experience**	9 years	6 years	7 years
**Setting**	Outpatient	Outpatient	Outpatient and inpatient
**Education**	Master	Vocational school	Vocational school
**Clinical care**	6 h/week	41 h/week	35 h/week
**How many patients with osteoporosis do you treat on average per month?**	2-3 patients	20 patients	50 patients
**Self-rated level of experience with osteoporosis: scale 0-10?**	5	6	7-8
**Do you use guidelines in your daily work?**	Partially	No	No
**Do you know any (physiotherapeutic) guidelines on osteoporosis, including the DVO guidelines?**	No	No	No

Supplementary material 8: Physiotherapists' comments and recommendations on the D2 draft

Comment	Comment	Decision	Reasoning/justification
1	Text difficult to read due to some statistical data.	No changes.	Some statistical data and wording are common for a guideline.
2	Training dosages only in table not in the text.	No changes.	In analogy to the original English guideline, no changes were made.
3	Switching between SIGN and APTA recommendations.	No changes.	In analogy to the original English guideline, no changes were made.
4	Further additions to the glossary (e.g. "long-term", "impact training").	"long-term" (*langfristig*): not included in the Glossary.<br>"impact training" (Impact-Training): included in the glossary.	"long-term": The guideline explains "long-term" in several instances (6 to 48 months).<br>"impact training": We considered this a useful term to be included in the glossary.
5	Page 4: Division by body region: not in the rest of the document.	No changes.	In analogy to the original English guideline, no changes were made.
6	Use clearer colour differences to differentiate the exercise types.<br><br>Use clearer colour differences of the training forms in green and purple tones.	In the D2 draft, the colours blue, orange, red, green, purpl, and turquoise were used. We agreed with the experts, that especially the colours green and red are not good since most color-blind people have a red-green color vision deficiency. However, we checked the W3C Web Content Accessibility Guidelines (https://www.w3.org) and decided to change the colors to:<br>1. Dark blue (#003366)<br>2. Dark purple (#4B0082)<br>3. Dark brown (#5C4033)<br>4. Petrol/turquoise (#007B7A)	The W3C Web Content Accessibility Guidelines recommend: "Color is not used as the only visual means of conveying information, indicating an action, prompting a response, or distinguishing a visual element."

		5. Dark pink/fuchsia(#D12C8E)<br>6. Dark blue-green (#005757)	
7	Page 21, line 422: Content discrepancy with table regarding the evidence.	No changes.	We checked this statement but could not identify any discrepancy with any information given in the tables.
8	Examples of exercise types partly not suitable (page 18, line 315).	No changes.	We checked this recommendation ("SIGN recommendation: Combinations of closed kinetic chain (low force) (eg, progressive strengthening, balance training, endurance training), paired with open kinetic chain (high force) (eg, flexibility/stretching, progressive strengthening, endurance training) should be considered to optimize bone health.") and considered these exercise types as suitable.
9	Effects on the spine or femoral neck are sometimes ambiguously formulated.	No changes.	We checked the whole guideline document, but could not find any ambiguously formulated phrases.
10	"Dynamic-weight-bearing" is misleadingly worded, functional instead?	No changes.	In analogy to the original English guideline, no changes were made.
11	Is it necessary to mention the methodical processes (p.10ff.)?	No changes.	In line with national and international standards, we considered it necessary to mention the methodical processes of the guidelines.
12	Addressing in "we form" is misleading.	We reworded some passages to avoid the "we form".	We followed this recommendation to increase the comprehensibility of the document by using neutral wordings or the term used by Hartley et al. in several other instances ("the guideline

			team”; German: “APTA-Leitlinien-Team”).
13	The term “exercise physiologists” (*Sportphysiologe*) is misleading.	No changes.	An exercise physiologist is not a very common profession in Germany. However, these experts work in the German healthcare system and, in analogy to the original English guideline, no changes were made.
14	Mention comparative studies earlier in the document.	No changes.	In analogy to the original English guideline, no changes were made.

Supplementary material 9: Characteristics of the patients with osteoporosis (n = 2)

**Participant**	**Patient A**	**Patient B**
**Sex**	Female	Female
**Age**	80 years	75 years
**Osteoporosis diagnosis since**	15 years	1 year
**Medication**	Ibandronate injection every 3 months, vitamin D and calcium complex	None
**Medical history**	▪ distal radius fracture due to a fall (1 year ago)<br>▪ L2, L4, and L5 fractures without trauma (4 years ago)<br>▪ bronchial carcinoma (cortisone, immobility; 2 years ago)	▪ no falls or injuries<br>▪ lung disease (cortisone, immobility) within the last two years
**Physiotherapy experience**	Yes	Yes
**Self-rated level of osteoporosis-knowledge: scale 0-10?**	7-8	2-3
**Osteoporosis support group attended?**	No	No
**Received specific training recommendations related to osteoporosis?**	Strengthening exercises of the whole body with dumbbells or therabands as part of an osteoporosis sports group, but not in the physiotherapy treatment	No
**Regular exercise (e.g., balance, bone density, muscle strength?)**	1x/week rehabilitation exercise (strengthening and balance exercises), strengthening exercises with dumbbells, and spinal self-mobilisation exercises	3x/week full-body strength training in the gym, 1x/week water aerobics

Supplementary material 10: Patients’ evaluation of guideline-feasibility (D2 draft)

Question	Patient A	Patient B
**Which exercises do not seem to be easy to implement?**	Recommendations for postmenopausal women appear feasible.	Recommendations for postmenopausal women appear feasible.
**"Standing weightlifting, walking/jogging or skipping, climbing stairs with weighted vests, jumping, running, dancing, vibration platforms."**<br>**Are you aware of any exercises of this type that could be beneficial for osteoporosis?**<br>**Could you do it?**	Unknown.<br>Implementation not possible due to health restrictions.	Unknown.<br>Implementation not possible due to health restrictions, except for weight lifting while standing.
**“One-leg standing 1 minute per leg, 3 times daily, for 24 weeks.”**<br>**Are you aware of this exercise type that could be beneficial for osteoporosis?**<br>**Could you do it?**	Unknown.<br>The exercise can be performed with modifications (such as holding onto something nearby or adjusting the duration).	Unknown.<br>Currently, a 30-second one-leg stand is possible.
**Any other comments?**	None.	None.

Supplementary material 11: Original guideline text and final German translations

Original English Version	German Version
Physical Therapist Management of Patients With Suspected or Confirmed Osteoporosis: A Clinical Practice Guideline From the Academy of Geriatric Physical Therapy	Physiotherapeutische Behandlung von Menschen mit vermuteter oder bestätigter Osteoporose: Eine klinische Praxisleitlinie der „Academy of Geriatric Physical Therapy“
**ABSTRACT**<br>A clinical practice guideline on physical therapist management of patients with suspected or confirmed osteoporosis was developed by a volunteer guideline development group (GDG) that was appointed by the Academy of Geriatric Physical Therapy (APTA Geriatrics). The GDG consisted of an exercise physiologist and 6 physical therapists with clinical and methodological expertise.<br>The guideline was based on a systematic review of existing clinical practice guidelines, followed by application of the ADAPTE methodological process described by Guidelines International Network for adapting guidelines for cultural and professional utility.<br>The recommendations contained in this guideline are derived from the 2021 Scottish Intercollegiate Guideline Network (SIGN) document: Management of Osteoporosis and the Prevention of Fragility Fractures. These guidelines are intended to assist physical therapists practicing in the United States, and implementation in the context of the US health care system is discussed.	**ZUSAMMENFASSUNG**<br>Eine freiwillige, von der „Academy of Geriatric Physical Therapy“ (APTA Geriatrics) eingesetzte Leitlinienentwicklungsgruppe, hat eine klinische Praxisleitlinie zur physiotherapeutischen Behandlung von Patient:innen mit vermuteter oder bestätigter Osteoporose erarbeitet. Die Leitlinienentwicklungsgruppe bestand aus einem Sportphysiologen und 6 Physiotherapeut:innen mit klinischer und methodologischer Expertise.<br>Die Leitlinie basiert auf einem systematischen Review bestehender klinischer Praxisleitlinien, gefolgt von der Anwendung des ADAPTE-Methodenprozesses, der vom „Guidelines International Network“ zur Anpassung von Leitlinien für kulturelle und professionsspezifische Zwecke beschrieben wurde.<br>Die in dieser Leitlinie enthaltenen Empfehlungen sind aus der Leitlinie „Management of Osteoporosis and the Prevention of Fragility Fractures“ des „Scottish Intercollegiate Guideline Network“ (SIGN) aus dem Jahr 2021 abgeleitet. Diese Empfehlungen sollen Physiotherapeut:innen in Deutschland, Österreich und der Schweiz unterstützen.
Key Words: bone health, clinical practice guideline, osteoporosis, physical therapy	Schlüsselwörter: Knochengesundheit, klinische Praxisleitlinie, Osteoporose, Physiotherapie
**INTRODUCTION**	**EINFÜHRUNG**
Aim of the Document<br>The Academy of Geriatric Physical Therapy has an ongoing effort to create evidence-based practice guidelines for the physical therapy management of older adults. The purpose of this document was to provide evidence-based recommendations on exercise interventions that impact bone mineral density (BMD) in individuals with osteoporosis.	Ziel des Dokuments<br>Die „Academy of Geriatric Physical Therapy“ arbeitet kontinuierlich an der Entwicklung von evidenzbasierten Praxisleitlinien für die physiotherapeutische Behandlung älterer Menschen. Der Zweck dieses Dokuments war es, evidenzbasierte Empfehlungen zu Bewegungsinterventionen zu geben, die sich auf die Knochendichte bei Personen mit Osteoporose auswirken.
Statement of Intent<br>These recommendations are not intended to be construed or to serve as a standard of medical care. Standards of care are determined on the basis of all clinical data available for an individual patient and are subject to change as scientific knowledge, technology advances, and patterns of care evolve. These parameters of practice should be considered as	Absichtserklärung<br>Diese Empfehlungen sind nicht dazu gedacht, als Standard für die medizinische Versorgung ausgelegt zu werden oder als solcher zu dienen. Behandlungsstandards werden auf der Grundlage aller für eine einzelne Person (Patient:in) verfügbaren klinischen Daten festgelegt und können sich im Zuge wissenschaftlicher Erkenntnisse, des technischen Fortschritts und der

Original English Version	German Version
guidelines only. Adherence to them will not ensure a successful outcome in every patient, nor should they be construed as including all proper methods of care or excluding other acceptable methods of care aimed at the same results. The ultimate judgment regarding a particular clinical procedure or treatment plan must be made in light of the clinical data presented by the patient; the diagnostic and treatment options available; and the patient's values, expectations, and preferences. However, we suggest that significant departures from accepted recommendations should be documented in the patient's medical records at the time the relevant clinical decision is made.	Entwicklung von Behandlungsmustern ändern. Diese klinische Praxisleitlinie (im Weiteren „Leitlinie") sollte lediglich als Empfehlung dienen. Ihre Einhaltung garantiert weder bei jeder Patient:in ein erfolgreiches Ergebnis, noch sollte sie so ausgelegt werden, dass sie alle geeigneten Behandlungsmethoden einschließt oder andere akzeptable Behandlungsmethoden ausschließt, die auf die gleichen Ergebnisse abzielen. Die endgültige Entscheidung über ein bestimmtes klinisches Verfahren oder einen Behandlungsplan muss unter Berücksichtigung der von der Patient:in vorgelegten klinischen Daten, der verfügbaren Diagnose- und Behandlungsoptionen sowie der Werte, Erwartungen und Präferenzen der Patient:in getroffen werden. Die Autor:innen der Leitlinie schlagen jedoch vor, dass wesentliche Abweichungen von anerkannten Empfehlungen zum Zeitpunkt der entsprechenden klinischen Entscheidung in den Patientenakten dokumentiert werden sollten.
Background<br>Osteoporosis is a systemic, metabolic bone disease characterized by low bone mass, impaired bone quality, and increased susceptibility of low-trauma fracture. The World Health Organization operationally defines osteoporosis via a dual-energy x-ray absorptiometry BMD T score at the spine or hip of −2.5 or below that of sex- and race-matched young adults. This definition has been refined to include those with an elevated fracture risk based on the World Health Organization Fracture Risk Algorithm as another critical criterion. In addition, a clinical diagnosis of osteoporosis can be made when a low-trauma hip fracture occurs in the presence of normal BMD (eg, during a fall from a standing height) or when a low-trauma vertebral, proximal humeral, or pelvic fracture (and in some cases, a distal forearm fracture) occurs in an individual with osteopenia.	HINTERGRUND<br>Osteoporose ist eine systemische, metabolische Skeletterkrankung, die durch geringe Knochenmasse, beeinträchtigte Knochenqualität und erhöhte Anfälligkeit für Niedrig-Trauma-Fraktur gekennzeichnet ist. Die Weltgesundheitsorganisation definiert Osteoporose über einen Knochendichte-T-Score (ein Maß für die Knochendichte) an der Wirbelsäule oder Hüfte von -2,5 oder weniger im Vergleich zu jungen Erwachsenen gleichen Geschlechts oder ethnischen Hintergrunds (operationale Definition der WHO) [1–3]. Diese Definition wurde überarbeitet, um auch Personen mit erhöhtem Frakturrisiko auf der Grundlage des Frakturrisiko-Algorithmus der Weltgesundheitsorganisation als weiteres wichtiges Kriterium einzubeziehen [4]. Darüber hinaus kann eine klinische Diagnose von Osteoporose gestellt werden, wenn eine Niedrig-Trauma-Fraktur der Hüfte auftritt, obwohl die Knochendichte normal ist (z. B. bei einem Sturz aus dem Stand) oder wenn eine Niedrig-Trauma-Fraktur der Wirbelsäule, des proximalen Humerus oder des Beckens (und in einigen Fällen eine Fraktur des distalen Unterarmes) bei einer Person mit Osteopenie auftritt [4].
The prevalence of osteoporosis based on data from Centers for Disease Control and Prevention National Health and Nutrition Examination Survey estimates that more than 10 million adults older than 50 years had osteoporosis in 2010, with a higher prevalence in women (∼30%) than in men (∼16%). The risk for osteoporosis increases with advancing age, with women having a higher risk of fracture than men. The lifetime risk of osteoporotic fracture for women and men is approximately 50% and 20%, respectively.	Basierend auf Daten des „National Health and Nutrition Examination Survey" des „Centers for Disease Control and Prevention" litten in den USA im Jahr 2010 schätzungsweise mehr als 10 Millionen Erwachsene über 50 Jahren an Osteoporose, wobei die Prävalenz bei Frauen höher war (∼30 %) als bei Männern (∼16 %) [5, 6]. Das Risiko für Osteoporose steigt mit zunehmendem Alter, wobei Frauen ein höheres Frakturrisiko als Männer haben [5, 7]. Das Lebenszeitrisiko für osteoporotische Frakturen beträgt für Frauen etwa 50 % und für Männer etwa 20 % [8–10].

Original English Version	German Version
Fragility fractures that occur from low-trauma forces, such as those experienced during a fall from standing height, result in increased morbidity, mortality, hospitalizations and clinical burden, and reduced quality of life (QOL). Worldwide, osteoporosis causes more than 8.9 million fractures annually, with an osteoporotic fracture occurring every 3 seconds. More than 2 million osteoporosis-related fractures were reported in the United States in 2005 with hip, spine, and distal radius fractures making up two-thirds of osteoporosis-associated fractures. Patients who suffer from a hip or vertebral fracture have decreased life expectancy compared with population-based controls, are more likely to be women, and carry a higher mortality rate in those with hip fractures and those who suffered subsequent fractures.	Fragilitätsfrakturen, die durch ein geringes Trauma verursacht werden, wie z. B. bei einem Sturz aus dem Stand, führen zu erhöhter Morbidität, Mortalität, Krankenhausaufenthalten und klinischer Belastung sowie zu einer verminderten Lebensqualität. Weltweit verursacht Osteoporose jährlich mehr als 8,9 Millionen Frakturen, wobei alle 3 Sekunden eine osteoporotische Fraktur auftritt [11]. Im Jahr 2005 wurden in den Vereinigten Staaten mehr als 2 Millionen osteoporosebedingte Frakturen gemeldet, wobei zwei Drittel der osteoporosebedingten Frakturen Hüft-, Wirbelsäulen- und distale Radiusfrakturen umfassten [12]. Patient:innen, die eine Hüft- oder Wirbelsäulenfraktur erleiden, haben eine verringerte Lebenserwartung im Vergleich zu einer bevölkerungsbasierten Kontrollgruppe und sind mit größerer Wahrscheinlichkeit weiblich. Außerdem haben Patient:innen mit Hüftfraktur und solche mit Folgefrakturen eine höhere Sterblichkeitsrate [13–15].
Physical inactivity is a modifiable risk factor that contributes to the development of low bone mass, osteoporosis, and increased fall risk. As the incidence of osteoporosis and fractures in individuals older than 65 years continues to increase, the development of appropriate physical activity guidelines for bone health requires clear clinical standards implemented across different clinical settings. Guidelines are necessary to promote optimal care across a wide range of clinical settings, such as acute and post–acute care, outpatient, and community-based wellness programs.	Bewegungsmangel ist ein modifizierbarer Risikofaktor, der zur Entwicklung von geringer Knochenmasse, Osteoporose und erhöhtem Sturzrisiko beiträgt. Da die Inzidenz von Osteoporose und Frakturen bei Menschen über 65 Jahren weiter zunimmt, erfordert die Entwicklung geeigneter Bewegungsempfehlungen für die Knochengesundheit klare klinische Standards, die in verschiedenen klinischen Settings umgesetzt werden [16]. Leitlinien sind notwendig, um eine optimale Versorgung in einem breiten Spektrum von klinischen Bereichen zu fördern, z. B. in der akuten und postakuten Versorgung, im ambulanten Bereich und in gemeindebasierten Gesundheitsprogrammen.
Since BMD is an important predictor of fracture risk, we based the recommendations in this document on changes in BMD. When prescribing physical activity interventions to promote bone health, it is important to understand the characteristics of physical activity and exercise that are sufficient to effect BMD without causing adverse outcomes such as fractures.	Da die Knochendichte ein wichtiger Prädiktor für das Frakturrisiko ist, basieren die Empfehlungen in diesem Dokument auf Veränderungen der Knochendichte. Bei der Verordnung von Maßnahmen der Bewegungstherapie zur Förderung der Knochengesundheit ist es wichtig, die Ausführung und Dosierung von Bewegung und Training zu verstehen, die ausreichen, um die Knochendichte zu beeinflussen, ohne nachteilige Folgen wie Frakturen hervorzurufen [17–20].

Original English Version	German Version
Scope, Purpose, and Target Users<br>This document presents the evidence-based exercise recommendations adapted from the Scottish Intercollegiate Guideline Network (SIGN): *Management of Osteoporosis and the Prevention of Fragility Fractures.* Although the SIGN clinical practice guideline (CPG) addressed multiple aspects of the management of osteoporosis in premenopausal and postmenopausal women, and men, this adapted document includes only the sections dealing with exercise interventions. Furthermore, it should be noted that while the SIGN CPG did not address osteoporosis in transgender individuals, a recent review concluded that treatment of osteoporosis in transgender persons follows the same guidelines as cisgender persons.<br>Optimizing bone health may also reduce fracture risk. However, this adapted document did not directly assess any recommendations or outcomes related to fracture or fall rate/risk. The recommendations included in these adapted guidelines are intended to facilitate decision making by physical therapists treating individuals with known or suspected osteoporosis. In addition, these recommendations may inform consumers of physical therapy as well as other health care providers about recommended exercise interventions for adults with or at risk for osteoporosis.	Umfang, Zweck und Zielgruppen<br>Dieses Dokument enthält die evidenzbasierten Trainingsempfehlungen, die aus dem „Scottish Intercollegiate Guideline Network" (SIGN) übernommen wurden: „Management of Osteoporosis and the Prevention of Fragility Fractures" [21]. Obwohl die SIGN-Leitlinie mehrere Aspekte des Managements von Osteoporose bei Frauen vor und nach der Menopause sowie Männern beinhaltet, enthält dieses adaptierte Dokument nur die Abschnitte, die sich mit Trainingsinterventionen befassen. Darüber hinaus ist anzumerken, dass die SIGN-Leitlinie zwar nicht auf die Osteoporose bei transgeschlechtlichen Personen eingeht, ein kürzlich durchgeführtes Review jedoch zu dem Schluss kommt, dass die Behandlung der Osteoporose bei transgeschlechtlichen Personen den gleichen Richtlinien folgt wie bei cisgeschlechtlichen Personen [22].<br>Die Optimierung der Knochengesundheit kann auch das Frakturrisiko verringern. In diesem adaptierten Dokument wurden jedoch keine direkten Empfehlungen oder Ergebnisse in Bezug auf Frakturen oder das Sturzrisiko berücksichtigt. Die in dieser adaptierten Leitlinie enthaltenen Empfehlungen sollen Physiotherapeut:innen, die Personen mit bekannter oder vermuteter Osteoporose behandeln, die Entscheidungsfindung erleichtern. Darüber hinaus können diese Empfehlungen Patient:innen und andere Gesundheitsdienstleister über die empfohlenen Trainingsinterventionen für Erwachsene mit Osteoporose oder Osteoporoserisiko informieren.
**METHODS**	**METHODEN**
Guideline Development Group<br>The Academy of Geriatric Physical Therapy (APTA Geriatrics), a component of the American Physical Therapy Association (APTA), appointed a multidisciplinary team of researchers and clinicians with expertise in guideline development or the management of osteoporosis to develop an evidence-based document focused on the management of osteoporosis through physical therapy–directed exercise interventions in adults.	Leitlinienentwicklungsgruppe der APTA Geriatrics<br>Die „Academy of Geriatric Physical Therapy" (APTA Geriatrics), ein Teil der „American Physical Therapy Association" (APTA), hat ein multidisziplinäres Team aus Forschenden und Kliniker:innen mit Fachkenntnissen in der Entwicklung von Leitlinien oder der Behandlung von Osteoporose mit der Ausarbeitung eines evidenzbasierten Dokuments beauftragt, das sich auf die Behandlung von Osteoporose durch physiotherapeutisch angeleitete Trainingsinterventionen bei Erwachsenen konzentriert.

Original English Version	German Version
CPG Search<br>To identify existing guidelines on improving or mitigating the loss of BMD through exercise, we conducted a comprehensive search for osteoporosis-related CPGs in the following repositories: National Guideline Clearinghouse, Trip Database, Scottish Intercollegiate Guidelines Network, National Institute for Health and Clinical Excellence, National Health Service, Physiotherapy Evidence Database, Occupational Therapy Systematic Evaluation of Evidence, and Guidelines International Network.<br>The following broad search terms were included: osteoporosis, osteopenia, low bone density, physical activity, exercise, physical therapy, intervention, and treatment. Inclusion criteria included publication within 5 years of search (since January 1, 2013), English language, female-male inclusive, and relevant to exercise in the physical therapy management of patients with osteoporosis.	Leitlinien-Suche<br>Das APTA-Leitlinien-Team führte eine umfassende Suche nach osteoporosebezogenen Leitlinien in den folgenden Quellen durch, um bestehende Leitlinien zum Entgegenwirken des Verlusts an Knochendichte durch Bewegung zu finden: National Guideline Clearinghouse, Trip Database, Scottish Intercollegiate Guidelines Network, National Institute for Health and Clinical Excellence, National Health Service, Physiotherapy Evidence Database, Occupational Therapy Systematic Evaluation of Evidence, und Guidelines International Network. Die folgenden Suchbegriffe wurden verwendet: „osteoporosis, osteopenia, low bone density, physical activity, exercise, physical therapy, intervention, treatment". Die Einschlusskriterien umfassten Veröffentlichungen innerhalb der letzten 5 Jahre (seit dem 1. Januar 2013), englische Sprache, geschlechtsübergreifend und sich dem Training im Rahmen der physiotherapeutischen Behandlung von Patient:innen mit Osteoporose widmend.
Our initial search identified the 55 unique abstracts to review for inclusion criteria. We excluded 20 references that were not CPGs, 7 that were not related to osteoporosis, 5 that were outdated, 4 that addressed only diagnosis or pharmacological management, and 2 that included only 1 sex. We reviewed the full text of the remaining 17 to determine whether they included recommendations on physical activity or exercise. Six of the remaining CPGs addressed exercise or physical activity in the management of osteoporosis.	Bei der ersten Suche wurden 55 Abstracts identifiziert, die auf Einschlusskriterien überprüft wurden. Es wurden 20 Referenzen ausgeschlossen, bei denen es sich nicht um Leitlinien handelte, 7, die sich nicht auf Osteoporose bezogen, 5, die veraltet waren, 4, die sich nur mit der Diagnose oder der pharmakologischen Behandlung befassten, und 2, die nur ein Geschlecht umfassten. Das APTA-Leitlinien-Team überprüfte den Volltext der verbleibenden 17 Leitlinien, um festzustellen, ob sie Empfehlungen zu Bewegung und Training enthielten. Sechs der verbleibenden Leitlinien befassten sich mit Bewegung oder Training in der Behandlung von Osteoporose [21, 23–27].
Because our initial search identified 6 CPGs addressing the use of exercise in managing patients with osteoporosis, the team explored the possibility of adapting an existing CPG to create recommendations for physical therapists for exercise prescription to mitigate BMD loss in patients with osteoporosis. To guide this process, we used the methodology developed by the ADAPTE Collaboration, an international collaboration of researchers, guideline developers, and guideline implementers.	Die anfängliche Suche ergab 6 Leitlinien, die sich mit dem Einsatz von Training in der Behandlung von Patient:innen mit Osteoporose befassen. Daher untersuchte das APTA-Leitlinien-Team die Möglichkeit, eine bestehende Leitlinie anzupassen, um Empfehlungen für Physiotherapeut:innen zur Verordnung von Training zu erstellen, um den Knochendichte-Verlust bei Patient:innen mit Osteoporose zu verringern. Als Leitfaden für diesen Prozess diente die Methodik der „ADAPTE Collaboration", einer internationalen Zusammenarbeit von Forschenden, Leitlinienentwickler:innen und Leitlinienumsetzenden.
ADAPTE Collaboration developed and validated a generic adaptation process that was endorsed by the Guideline International Network (G-I-N). The ADAPTE method as described in the Guideline Adaptation: A Resource Toolkit was used to determine whether existing CPGs could be adapted to meet our stated aims.[29] The ADAPTE method provided a systematic approach to adapting CPGs originally developed for use in one context for use by	Die „ADAPTE Collaboration" entwickelte und validierte einen generischen Anpassungsprozess, der vom „Guideline International Network" (G-I-N) unterstützt wurde [28]. Um die festgelegten Ziele zu erreichen, wurde die ADAPTE-Methode verwendet, so wie sie im „Guideline Adaptation: A Resource Toolkit" beschrieben ist [29]. So wurde überprüft, ob bestehende Leitlinien angepasst werden konnten. Die ADAPTE-Methode bietet einen systematischen Ansatz zur

Original English Version	German Version
a different group in a different culture (see the Figure). The ADAPTE method begins by evaluating existing CPGs based upon their quality, rigor, and relevance to the practice of physical therapy.	Anpassung von Leitlinien an einen anderen kulturellen Kontext (siehe Abbildung 1) [29]. Die ADAPTE-Methode beginnt mit der Bewertung bestehender Leitlinien basierend auf ihrer Qualität, wissenschaftlichen Strenge und Relevanz für die physiotherapeutische Praxis.
**The ADAPTE Process**<br>To evaluate existing CPGs on their quality and rigor, we used the Appraisal of Guidelines for Research & Evaluation II (AGREE II) instrument. The AGREE II instrument consists of 23 items scored on a 7-point response scale comprising 6 quality-related domains: scope and purpose, stakeholder involvement, rigor of development, clarity of presentation, applicability, and editorial independence.<br>Before scoring the CPGs, all reviewers completed AGREE II online training. Training was followed by all reviewers scoring 3 CPGs to examine consistency in using the AGREE II instrument. Once consistency was achieved, pairs of reviewers used the AGREE II instrument to independently score the quality and rigor of their assigned CPGs. When incongruent scoring occurred, the study coordinator performed a third appraisal as a mediator with a follow-up discussion if needed.	**Der ADAPTE-Prozess**<br>Um die Qualität und wissenschaftliche Strenge bestehender Leitlinien zu bewerten, wurde das Instrument „Appraisal of Guidelines for Research & Evaluation II" (AGREE II) verwendet [30]. Das AGREE II-Instrument besteht aus 23 Items, die auf einer 7-Punkte-Skala bewertet werden und sich auf 6 Bereiche der Leitlinien-Qualität beziehen: Umfang und Zweck, Beteiligung von Interessengruppen, wissenschaftliche Strenge der Entwicklung, Klarheit der Präsentation, Anwendbarkeit und redaktionelle Unabhängigkeit. Vor der Bewertung der Leitlinien absolvierten alle Gutachtenden eine AGREE II-Online-Schulung [31]. Im Anschluss an die Schulung bewerteten alle Gutachtenden 3 Leitlinien, um die Konsistenz bei der Anwendung des AGREE II-Instruments zu prüfen. Sobald eine Übereinstimmung erreicht war, benutzten Gutachterpaare das AGREE II-Instrument, um unabhängig voneinander die Qualität und wissenschaftliche Strenge der ihnen zugewiesenen Leitlinien zu bewerten. Bei inkongruenten Bewertungen nahm der Studienkoordinator eine dritte Bewertung als Mediator vor und führte bei Bedarf eine Nachbesprechung durch.
Table 1 presents the AGREE II CPG Rigor and Quality Scores for the 6 scored CPGs. Consistent with previous APTA Geriatrics documents, we included only CPGs with AGREE II scores of 50% for further consideration for adaptation, reducing the total to 3.	Tabelle 1 zeigt die AGREE II-Bewertungen zur Qualität und wissenschaftlichen Strenge der 6 bewerteten Leitlinien. In Anlehnung an frühere Dokumente der „APTA Geriatrics" wurden nur Leitlinien mit AGREE II-Bewertungen von mindestens 50 % für eine weitere Berücksichtigung bei der Anpassung eingeschlossen, was die Gesamtzahl auf 3 reduzierte [32].
The team evaluated these 3 CPGs for relevance to the use of exercise interventions by physical therapists in managing patients with osteoporosis. We reviewed: (1) *SIGN publication no. 142: Management of osteoporosis and the prevention of fragility fractures*; (2) *Too Fit to Fracture: exercise recommendations for individuals with osteoporosis or osteoporotic vertebral fractur*e; and (3) *National Osteoporosis Guideline Group 2017: Clinical guideline for the prevention and treatment of osteoporosis.*	Das APTA-Leitlinien-Team bewertete diese 3 Leitlinien auf ihre Relevanz für den Einsatz von Trainingsinterventionen durch Physiotherapeut:innen bei der Behandlung von Patient:innen mit Osteoporose. Das APTA-Leitlinien-Team begutachtete: (1) "SIGN publication no. 142: Management of osteoporosis and the prevention of fragility fractures" [21]; (2) "Too Fit to Fracture: exercise recommendations for individuals with osteoporosis or osteoporotic vertebral fracture" [27]; und (3) "National Osteoporosis Guideline Group 2017: Clinical guideline for the prevention and treatment of osteoporosis" [26].
The SIGN CPG had both the highest overall AGREE II score and the most comprehensive scope of exercise recommendations. The team agreed that the other 2 CPGs did not provide additional information beyond that addressed by the SIGN CPG. We therefore decided to adapt the SIGN CPG for use by physical therapists managing patients with osteoporosis in the United States.	Die SIGN-Leitlinie hatte sowohl die höchste Gesamtbewertung nach AGREE II als auch die umfassendsten Trainingsempfehlungen. Das APTA-Leitlinien-Team stimmte darin überein, dass die anderen beiden Leitlinien keine zusätzlichen Informationen über die von der SIGN-Leitlinie behandelten Aspekte lieferten. Das APTA-Leitlinien-Team entschied sich daher, die SIGN-

Original English Version	German Version
	Leitlinie für die Anwendung durch Physiotherapeut:innen bei der Behandlung von Patient:innen mit Osteoporose in den USA anzupassen.
The SIGN CPG provided recommendations for the comprehensive management of osteoporosis and prevention of factures. Because our purpose was limited to recommendations to guide physical therapists in prescribing physical activity or exercise interventions to maintain or improve bone density, we addressed only sections of the SIGN CPG dealing with physical activity and exercise interventions. The SIGN CPG addressed exercise interventions for postmenopausal women, premenopausal women, and men.	Die SIGN-Leitlinie enthält Empfehlungen für das umfassende Management von Osteoporose und die Prävention von Frakturen. Da das APTA-Leitlinien-Team sich auf Empfehlungen beschränkte, die Physiotherapeut:innen bei der Verordnung von Bewegung oder Training zur Erhaltung oder Verbesserung der Knochendichte unterstützen sollen, befassten sie sich nur mit den Abschnitten der SIGN-Leitlinie, die sich mit Bewegung und Trainingsinterventionen befassen. Die SIGN-Leitlinie befasst sich mit Trainingsinterventionen für Frauen vor und nach der Menopause sowie für Männer.
Exercise recommendations in the SIGN document were based on the findings from 8 systematic reviews. The SIGN CPG was published in 2015 and updated in 2020 and 2021. To evaluate the currency of the SIGN exercise recommendations, we contacted the Scottish Intercollegiate Guidelines Network in April 2020. They indicated that while they were updating the SIGN CPG (*Management of osteoporosis and the prevention of fragility fractures*), the exercise recommendations had not changed and were being republished verbatim in the 2021 update (A. Stein, email communication, April 9, 2020).	Die Trainingsempfehlungen im SIGN-Dokument basieren auf den Ergebnissen von 8 systematischen Reviews. Die SIGN-Leitlinie wurde 2015 veröffentlicht und in den Jahren 2020 und 2021 aktualisiert. Um die Aktualität der SIGN-Trainingsempfehlungen zu bewerten, wurde im April 2020 das „Scottish Intercollegiate Guidelines Network“ kontaktiert. Es teilte mit, dass die SIGN-Leitlinie („Management of osteoporosis and the prevention of fragility fractures“) zwar aktualisiert wurde, die Trainingsempfehlungen sich jedoch nicht geändert hatten und in der Aktualisierung 2021 wortwörtlich übernommen wurden (A. Stein, E-Mail-Kommunikation, 9. April 2020).
SIGN recommendations were heavily based on a Cochran Library Systematic Review, which was revised in 2002 and 2011. Emerging research did not trigger changes to SIGN recommendations in 2021. The study team reviewed the SIGN exercise recommendations to determine the extent to which we needed to modify them to make them more relevant to physical therapist practice. We also reviewed the terminology used in the recommendations to assess consistency with the terminology used in the source studies and with terminology commonly used in the United States.	Die SIGN-Empfehlungen basierten überwiegend auf einem systematischen Review der Cochrane Library, das 2002 und 2011 überarbeitet wurde [33]. Neue Forschungsergebnisse führten nicht zu Änderungen der SIGN-Empfehlungen im Jahr 2021. Das APTA-Leitlinien-Team überprüfte die SIGN-Trainingsempfehlungen, um festzustellen, inwieweit diese modifiziert werden mussten, um sie für die physiotherapeutische Praxis relevanter zu machen. Das APTA-Leitlinien-Team überprüfte auch die in den Empfehlungen verwendete Terminologie, um die Übereinstimmung mit der in den Quellenstudien verwendeten Terminologie und mit der in den Vereinigten Staaten üblichen Terminologie zu beurteilen.
**Guideline Adaptation**	**Adaptation der Leitlinie**
The SIGN CPG used the definitions of exercise types described in the *Cochrane Library Systematic Review*: “Exercise for Preventing and Treating Osteoporosis in Postmenopausal Women.” The Cochrane Review described exercises as static or dynamic, weight-bearing or non–weight-bearing position, and involving high or low force (see Table 2).	Die SIGN-Leitlinie verwendet die im Cochrane Review „Exercise for Preventing and Treating Osteoporosis in Postmenopausal Women“ beschriebenen Definitionen von Trainingsformen [33]. Der Cochrane Review beschrieb Training als statisch oder dynamisch, in gewichtstragenden oder nicht gewichtstragenden Positionen und mit hoher oder geringer Krafteinwirkung (siehe Tabelle 2).
To provide physical therapists with the type of exercise information they would need to guide practice, we extracted detailed information on exercise mode, frequency and duration,	Um Physiotherapeut:innen die Informationen zur Verfügung zu stellen, die sie für ihre Praxis benötigen, hat das APTA-Leitlinien-Team aus den systematischen Reviews, auf die sich die

Original English Version	German Version
adverse events, and participant characteristics from the systematic reviews on which SIGN based their recommendations. Physical activity encompasses all bodily movements (ie, walking, cycling, active recreation) that are performed at any level of skill and for enjoyment. Exercise falls under the umbrella of physical activity and is planned, structured, and repetitive.	SIGN-Empfehlungen stützen, detaillierte Informationen über Trainingsart, Frequenz und Dauer, unerwünschte Ereignisse und Merkmale der Studienteilnehmenden extrahiert. Bewegung umfasst alle körperlichen Bewegungen (z. B. Gehen, Radfahren, aktive Freizeitgestaltung), die auf einem beliebigen Fertigkeitsniveau und zum Vergnügen ausgeführt werden. Training fällt unter den Begriff der Bewegung und ist geplant, strukturiert und wiederholend.
These components are the basis of exercise prescription that targets objective improvement or maintenance in physical health and/or fitness. Since this document is focused on improving bone health, we are contextualizing all recommendations under the term "exercise." We described the strength of the adapted recommendations based on criteria from the *APTA Clinical Practice Guideline Process Manual, Revised* (see Table 3).	Diese Komponenten bilden die Grundlage für Trainingsempfehlungen, die auf eine objektive Verbesserung oder Erhaltung der körperlichen Gesundheit und/oder Fitness abzielen [34]. Da der Schwerpunkt dieses Dokuments auf der Verbesserung der Knochengesundheit liegt, kontextualisierte das APTA-Leitlinien-Team alle Empfehlungen unter dem Begriff „Training". Das APTA-Leitlinien-Team hat die Stärke der angepassten Empfehlungen anhand von Kriterien aus dem „APTA Clinical Practice Guideline Process Manual, Revised" beschrieben (siehe Tabelle 3) [35].
**RESULTS**	**ERGEBNISSE**
Sign Exercise Recommendations for Postmenopausal Women	SIGN-Trainingsempfehlungen für Frauen nach der Menopause
Static weight-bearing exercise<br>*SIGN recommendations*: Static weight-bearing (SWB) exercise, for example, single-leg standing should be considered to slow the decline of hip BMD.<br>*SIGN summary*: A meta-analysis reported a statistically significant reduction in BMD decline from 1 small study of single-leg standing (mean difference in hip BMD between exercise and control groups: 2.42%; 95% CI, 0.73-4.10). No risk of fracture/falls or QOL outcomes were reported.	Statisches gewichtstragendes Training<br>SIGN-Empfehlungen: Statisches gewichtstragendes Training, z. B. Einbeinstand, sollte in Betracht gezogen werden, um den Rückgang der Hüft-Knochendichte zu verlangsamen.<br>SIGN-Zusammenfassung: Eine Meta-Analyse berichtet über eine statistisch signifikante Verringerung des Knochendichte-Rückgangs in einer kleinen Studie zum Einbeinstand (mittlerer Unterschied in der Hüft-Knochendichte zwischen Trainings- und Kontrollgruppen: 2,42 %; 95 % CI, 0,73-4,10). Es wurden keine Ergebnisse zum Frakturrisiko/Sturzrisiko oder zur Lebensqualität berichtet [33].

Original English Version	German Version
Dynamic weight-bearing exercise (low force)<br>*SIGN recommendations*: Walking, tai chi, and progressive-resistance strength training (such as closed kinetic chain weight training) should be considered to slow the decline of lumbar spine BMD.<br>*SIGN summary*: Dynamic weight-bearing exercise with low force (DWBLF) performed standing (ie, walking, tai chi) showed no effect on fracture outcomes.<br>Dynamic weight-bearing exercise with low force mitigated the decline in spine BMD as demonstrated in a meta-analysis of 7 studies (mean difference in spine BMD between exercise and control groups: 0.84%; 95% CI, 0.26-1.48); however, no effect was observed for hip BMD. Bone mineral density data at the femoral neck were inconsistent in showing a positive effect from walking. However, another systematic review demonstrated reduction in BMD decline associated with walking in 2 studies. No data are available on the effect of DWBLF on the risks of falls or QOL.	Dynamisches gewichtstragendes Training (geringe Krafteinwirkung)<br>SIGN-Empfehlungen: Gehen, Tai-Chi und progressives Krafttraining (z. B. Krafttraining in geschlossener kinetischer Kette) sollten in Betracht gezogen werden, um den Rückgang der Knochendichte der Lendenwirbelsäule zu verlangsamen.<br>SIGN-Zusammenfassung: Dynamisches gewichtstragendes Training mit geringer Krafteinwirkung, das im Stehen durchgeführt wird (d. h. Gehen, Tai-Chi), zeigte keine Wirkung auf die Fraktur-Outcomes [33]. Dynamisches gewichtstragendes Training mit geringer Krafteinwirkung reduzierten den Rückgang der Knochendichte in der Wirbelsäule, wie in einer Meta-Analyse von 7 Studien gezeigt wurde (mittlerer Unterschied in der Knochendichte der Wirbelsäule zwischen Trainings- und Kontrollgruppen: 0,84 %; 95 % CI, 0,26-1,48); für die Knochendichte der Hüfte wurde jedoch kein Effekt beobachtet [33]. Die Daten zur Knochendichte am Oberschenkelhals waren inkonsistent in Bezug auf einen positiven Effekt durch Gehen [36]. In einem anderen systematischen Review wurde jedoch in 2 Studien eine Verringerung des Rückgangs der Knochendichte durch Gehen nachgewiesen [37]. Es liegen keine Daten über die Auswirkungen von dynamischem, gewichtstragenden Training mit geringer Krafteinwirkung auf das Sturzrisiko oder die Lebensqualität vor.
Dynamic weight-bearing exercise (high force)<br>*SIGN recommendation*: No evidence-based recommendation provided. There was no statistically significant effect of this type of exercise on bone density at the spine or the hip.<br>*SIGN summary*: Dynamic weight-bearing exercise with high force (DWBHF) performed in a standing position includes these forms of exercise: jogging, jumping, running, dancing, and use of vibration platforms.<br>There was no effect on change in BMD of the spine reported in a meta-analysis of 4 studies involving DWBHF. Furthermore, high-impact only and odd-impact only protocols were ineffective in increasing BMD at any site. (Odd-impact was defined as aerobic or step classes, bounding exercises, agility exercises, and games in which movements included directional elements that the body is not normally accustomed to.) No data were available on the effects of high-force dynamic weight-bearing exercise on the risks of fracture, falls, or QOL.	Dynamisches gewichtstragendes Training (hohe Krafteinwirkung)<br>SIGN-Empfehlung: Keine evidenzbasierte Empfehlung möglich. Es wurde kein statistisch signifikanter Effekt dieser Trainingsform auf die Knochendichte an der Wirbelsäule oder der Hüfte festgestellt.<br>SIGN-Zusammenfassung: Dynamisches gewichtstragendes Training mit hoher Krafteinwirkung, das im Stehen durchgeführt wird, umfasst diese Formen der Bewegung: Joggen, Springen, Laufen, Tanzen und die Verwendung von Vibrationsplattformen. Es wurde keine Auswirkung auf die Veränderung der Knochendichte der Wirbelsäule in einer Meta-Analyse von 4 Studien mit dynamischem, gewichtstragendem Training mit hoher Krafteinwirkung berichtet [33]. Darüber hinaus waren Protokolle mit ausschließlich „high-impact“ Training und Protokolle mit „odd-impact“ Training ineffektiv bei der Erhöhung der Knochendichte an jeglicher Stelle [38] („odd-impact“ Training wurde definiert als Aerobic- oder Step-Kurse, Sprungübungen, Geschicklichkeitsübungen und Spiele, die Bewegungen mit Richtungswechsel enthielten, an die der Körper normalerweise nicht gewöhnt ist). Es lagen keine Daten über die Auswirkungen von dynamischem, gewichtstragendem Training mit hoher Krafteinwirkung auf die Risiken von Frakturen, Stürzen oder die Lebensqualität vor.

**Original English Version**	**German Version**
Non–weight-bearing exercise (low force)<br>*SIGN recommendation*: No evidence-based recommendation provided.<br>*SIGN summary*: Non–weight-bearing exercise with low force (NWBLF) includes high-repetition, low-load strength training with open kinetic chain weight exercises in a supported or seated position. No significant differences were observed for any BMD outcomes with low-force non–weight-bearing exercise, for example, seated low-load, high-repetition strength training. No data were available on the effects of non–weight-bearing exercise on the risks of fracture, falls, or QOL.	Nicht gewichtstragendes Training (geringe Krafteinwirkung)<br>SIGN-Empfehlung: Keine evidenzbasierte Empfehlung möglich.<br>SIGN-Zusammenfassung: Nicht gewichtstragendes Training mit geringer Krafteinwirkung umfasst Krafttraining mit hoher Wiederholungszahl und mit geringem Widerstand in der offenen kinetischen Kette in einer gestützten oder sitzenden Position. Es wurden keine signifikanten Unterschiede in Bezug auf Knochendichte-Outcomes bei nicht gewichtstragendem Training mit geringer Krafteinwirkung beobachtet, z. B. Krafttraining im Sitzen mit geringem Widerstand und hoher Wiederholungszahl [33]. Es lagen keine Daten zu den Auswirkungen von nicht gewichtstragendem Training auf das Frakturrisiko, das Sturzrisiko oder die Lebensqualität vor.
Non–weight-bearing exercise (high force)<br>*SIGN recommendation*: Progressive resistance strength training exercise (such as open kinetic chain weight training) should be considered to slow the decline of femoral neck BMD.<br>*SIGN summary*: Non–weight-bearing exercise with high force (NWBHF) includes progressive resistance strength training with open kinetic chain weight exercises in a supported or seated position.<br>The NWBHF exercise slowed the BMD decline at the spine (mean difference in spine BMD between exercise and control groups: 0.86%; 95% CI, 0.58-1.13, 8 studies) and neck of femur (mean difference in femoral neck BMD between exercise and control groups: 1.03%; 95% CI, 0.24-1.82, 8 studies). An increase in spine BMD of 0.006 g/cm^2 (95% CI, 0.002-0.011; *P* = .006, 14 studies) was demonstrated following NWBHF in a different meta-analysis. No data were available on the effects of non–weight-bearing high force exercise on the risks of fracture, falls, or QOL.	Nicht gewichtstragendes Training (hohe Krafteinwirkung)<br>SIGN-Empfehlung: Progressives Krafttraining (wie z. B. Training in der offenen kinetischen Kette) sollte in Betracht gezogen werden, um den Rückgang der Knochendichte im Schenkelhals zu verlangsamen.<br>SIGN-Zusammenfassung: Nicht gewichtstragendes Training mit hoher Krafteinwirkung umfasst progressives Krafttraining in der offenen kinetischen Kette in einer gestützten oder sitzenden Position. Ein nicht gewichtstragendes Training mit hoher Krafteinwirkung verlangsamte den Rückgang der Knochendichte in der Wirbelsäule (mittlerer Unterschied in der Wirbelsäulen-Knochendichte zwischen Trainings- und Kontrollgruppen: 0,86 %; 95 % CI, 0,58-1,13, 8 Studien) und am Oberschenkelhals (mittlerer Unterschied in der Oberschenkelhals-Knochendichte zwischen Trainings- und Kontrollgruppen: 1,03 %; 95 % CI, 0,24-1,82, 8 Studien) [33]. In einer anderen Meta-Analyse wurde ein Anstieg der Wirbelsäulen-Knochendichte um 0,006 g/cm2 (95% CI, 0,002-0,011; P = 0.006, 14 Studien) nach nicht gewichtstragendem Training mit hoher Krafteinwirkung nachgewiesen [39]. Es lagen keine Daten zu den Auswirkungen von nicht gewichtstragendem Training mit hoher Krafteinwirkung auf das Risiko von Frakturen, Stürzen oder Lebensqualität vor.

Original English Version	German Version
Combination of exercise types<br>*SIGN recommendation*: Combinations of closed kinetic chain (low force) (eg, progressive strengthening, balance training, endurance training), paired with open kinetic chain (high force) (eg, flexibility/stretching, progressive strengthening, endurance training) should be considered to optimize bone health.	Kombination von Trainingsformen<br>SIGN-Empfehlung: Zur Optimierung der Knochengesundheit sollten Übungen in geschlossener kinetischer Kette (geringe Krafteinwirkung) (z. B. progressives Krafttraining, Gleichgewichtstraining, Ausdauertraining) mit Übungen in offener kinetischer Kette (hohe Krafteinwirkung) (z. B. Flexibilität/Dehnung, progressives Krafttraining, Ausdauertraining) kombiniert werden.
SIGN summary: Risk of fractures in groups performing combinations of any 2 of the exercise types (SWB, DWBLF, DWBHF, NWBLF, and NWBHF) was significantly lower than that in controls (odds ratio: 0.33; 95% CI, 0.13-0.85, 2 studies).<br>A reduction in BMD decline at the spine was reported (mean difference in spine BMD between exercise and control groups immediately following intervention: 3.22%; 95% CI, 1.80-4.64, 4 studies). Combination of exercise types (COMB) exercise slowed total hip BMD decline when compared with controls (mean difference in total hip BMD between exercise and control groups: −1.07%; 95% CI, −1.58 to −0.56, 4 studies).<br>Impact protocols that included jogging mixed with walking and stair climbing, and protocols that incorporated impact exercise with high-magnitude force (resistance exercises) were effective at reducing bone density loss at the lumbar spine and femoral neck. Combined aerobics and high-intensity resistance exercises had a positive effect on BMD decline. Intervention with combined exercise programs had better effects on physical function, pain, and vitality domains than controls ($P < .05$). No data were available on the effects of these interventions on the risks of falls.	SIGN-Zusammenfassung: Das Frakturrisiko in den Gruppen, die Kombinationen von zwei der Trainingsformen (statisches gewichtstragendes Training; dynamisches gewichtstragendes Training mit geringer Krafteinwirkung; dynamisches gewichtstragendes Training mit hoher Krafteinwirkung; nicht gewichtstragendes Training mit geringer Krafteinwirkung; nicht gewichtstragendes Training mit hoher Krafteinwirkung) durchführten, war signifikant niedriger als in den Kontrollgruppen (Odds Ratio: 0,33; 95% CI, 0,13-0,85, 2 Studien) [33].<br>Es wurde über eine Verringerung des Knochendichte-Rückgangs an der Wirbelsäule berichtet (mittlerer Unterschied in der Wirbelsäulen-Knochendichte zwischen Trainings- und Kontrollgruppen unmittelbar nach der Intervention: 3,22 %; 95 % CI, 1,80-4,64, 4 Studien). Die Kombination verschiedener Trainingsformen verlangsamte den Rückgang der Gesamt-Knochendichte in der Hüfte im Vergleich zu den Kontrollgruppen (mittlerer Unterschied in der Gesamt-Knochendichte in der Hüfte zwischen Trainings- und Kontrollgruppen: -1,07 %; 95 % CI, -1,58 bis -0,56, 4 Studien) [33].<br>Trainingsprotokolle mit Stoßbelastungen, die Joggen in Kombination mit Gehen und Treppensteigen sowie Trainingsprotokolle, die Stoßbelastungsübungen mit hoher Intensität (Widerstandsübungen) beinhalteten, erwiesen sich als wirksam bei der Reduzierung des Knochendichteverlusts in der Lendenwirbelsäule und im Oberschenkelhals [38]. Kombiniertes Aerobic- und hochintensives Krafttraining hatte einen positiven Effekt auf die Verlangsamung des Knochendichteverlusts [37]. Interventionen mit kombinierten Trainingsprogrammen zeigten bessere Effekte auf die körperlichen Funktionen, Schmerzen und Vitalität als

Original English Version	German Version
	Kontrollinterventionen (P < 0,05) [40]. Es lagen keine Daten über die Auswirkungen dieser Interventionen auf das Sturzrisiko vor.
A systematic review and meta-analysis of moderate- to high-quality randomized clinical trials (RCTs), which looked at the effect of falls prevention exercise programs on fracture rates, reported a significant reduction in the rate of falls resulting in fracture, with a pooled estimated rate ratio of 0.39 (0.23-0.66, 6 studies, I^2 = 0%). While mixed populations were included, 77% of participants were postmenopausal women, and no subgroup analysis for men was performed.<br>The studies that decreased falls resulting in fractures included balance training and most were multicomponent, including other exercise types such as strengthening, flexibility, and endurance exercise. Another meta-analysis reported an overall fracture reduction in the exercise group compared with controls (relative risk: 0.49; 95% CI, 0.31-0.76). The findings of this study are limited by methodological flaws of individual studies, and potential for publication bias was noted by the authors.	Ein systematisches Review mit Meta-Analyse von randomisierten klinischen Studien (RCTs) mittlerer bis hoher Qualität, die die Auswirkungen von Trainingsprogrammen zur Sturzprävention auf die Frakturraten untersuchten, ergab eine signifikante Verringerung der Rate von Stürzen, die zu Frakturen führten, mit einem gepoolten geschätzten Ratenverhältnis (Rate Ratio) von 0,39 (0,23-0,66, 6 Studien, I2 = 0%). Obwohl gemischte Populationen einbezogen wurden, waren 77 % der Teilnehmenden Frauen nach der Menopause. Es wurde keine Subgruppenanalyse für Männer durchgeführt. Die Studien, die zu einer Verringerung der Stürze und der daraus resultierenden Frakturen führten, beinhalteten ein Gleichgewichtstraining und bestanden aus mehreren Komponenten wie Kraft-, Flexibilitäts- und Ausdauertraining [41]. In einer anderen Meta-Analyse wurde berichtet, dass die Zahl der Frakturen in der Trainingsgruppe im Vergleich zur Kontrollgruppe insgesamt zurückging (relatives Risiko: 0,49; 95 % CI, 0,31-0,76) [42]. Die Ergebnisse dieser Studie sind durch methodische Mängel der einzelnen Studien eingeschränkt, und die Autor:innen wiesen auf eine möglichen Publikationsbias hin.
**ADAPTED EXERCISE RECOMMENDATIONS FOR POSTMENOPAUSAL WOMEN**	**ADAPTIERTE TRAININGSEMPFEHLUNGEN FÜR FRAUEN NACH DER MENOPAUSE**
Adapted Exercise Recommendations for Postmenopausal Women to Slow Decline of BMD of the Hip and the Femoral Neck<br>Physical therapists should design and advise postmenopausal women to participate in long-duration (ie, minimum of 6-48 months) exercise programs consisting of static weight-bearing (SWB—SIGN terminology) exercises such as single-leg standing to slow the decline of BMD at the hip. Physical therapists should design and advise postmenopausal women to participate in long-duration, adequately dosed progressive resistance strength training exercises such as weight training either alone or in combination with impact exercise training such as jogging, walking, or aerobics to slow the decline of BMD of the femoral neck. (Grade B, recommendation based on moderate evidence)	Adaptierte Trainingsempfehlungen für Frauen nach der Menopause zur Verlangsamung des Knochendichteverlusts in Hüfte und Oberschenkelhals<br>Physiotherapeut:innen sollten für Frauen nach der Menopause langfristige Trainingsprogramme (d. h. mindestens 6-48 Monate) mit statischen, gewichtstragenden Übungen (statisches gewichtstragendes Training nach SIGN-Terminologie), wie Einbeinstand, zusammenstellen und anleiten, um den Rückgang der Knochendichte in der Hüfte zu verlangsamen.<br>Physiotherapeut:innen sollten für Frauen nach der Menopause langfristige, angemessen dosierte Krafttrainingsprogramme zusammenstellen und anleiten, entweder allein oder in Kombination mit Ausdauertraining, wie z. B. Joggen, Walken oder Aerobic, um den Rückgang der Knochendichte am Oberschenkelhals zu verlangsamen (Grad B, Empfehlung auf der Grundlage moderater Evidenz).

Original English Version	German Version
Adapted Exercise Recommendations for Postmenopausal Women to Slow Decline of BMD of the Lumbar Spine<br>Physical therapists should consider designing and advising postmenopausal women to participate in long-duration exercise programs consisting of walking, tai chi, progressive resistance strength training (such as weight training), and different combinations of exercise types to slow the decline of lumbar spine BMD. (SIGN terminology DWBLF, NWBHF, COMB). (Grade B, recommendation based on moderate evidence)	Adaptierte Trainingsempfehlungen für Frauen nach der Menopause zur Verlangsamung des Knochendichteverlusts in der Lendenwirbelsäule<br>Physiotherapeut:innen sollten in Erwägung ziehen, für Frauen nach der Menopause Langzeit-Trainingsprogramme zusammenzustellen und anzuleiten, die aus Gehen, Tai-Chi, progressivem Krafttraining (wie Training mit Gewichten) und verschiedenen Kombinationen aus Trainingsformen bestehen, um den Knochendichteverlust in der Lendenwirbelsäule zu verlangsamen (SIGN Terminologie: dynamisches gewichtstragendes Training mit geringer Krafteinwirkung, nicht gewichtstragendes Training mit hoher Krafteinwirkung, Kombination verschiedener Trainingsformen) (Grad B, Empfehlung basierend auf moderater Evidenz).
Postmenopausal Women: Adapted Summary Statement<br>Based upon evidence from the SIGN CPG systematic reviews, there is a small positive effect on BMD in postmenopausal women that is exercise mode and anatomical site specific. Static weight-bearing exercises such as single-leg stance had a modest impact on the decline in hip BMD. This finding was based solely on 1 RCT in Asian women without a clear diagnosis of osteoporosis; therefore, generalizability to other populations is unclear.<br>However, based upon positive findings and the low risk of adverse events, we agree that single-leg weight-bearing activities should be recommended. The specific dose is unclear, but 1 minute per leg, 3 times per week for 24 weeks had a beneficial effect.	Frauen nach der Menopause: Adaptierte Zusammenfassung<br>Auf der Grundlage der Erkenntnisse aus den systematischen Reviews der SIGN-Leitlinie gibt es einen geringen positiven Effekt auf die Knochendichte bei Frauen nach der Menopause, der von der Trainingsform und der anatomischen Region abhängt. Statisches gewichtstragendes Training (wie der Einbeinstand) hatte einen geringen Einfluss auf den Rückgang der Knochendichte in der Hüfte [33]. Dieses Ergebnis basierte lediglich auf einer RCT bei Frauen aus Asien ohne eindeutige Osteoporose-Diagnose; daher ist die Generalisierbarkeit auf andere Bevölkerungsgruppen unklar.<br>Aufgrund der positiven Ergebnisse und des geringen Risikos unerwünschter Ereignisse ist das APTA-Leitlinien-Team jedoch der Meinung, dass einbeiniges gewichtstragendes Training empfohlen werden sollte. Die spezifische Dosis ist unklar, aber 1 Minute pro Bein, dreimal pro Woche über 24 Wochen, hatte einen positiven Effekt.
The DWBLF exercises slowed BMD loss at the lumbar spine but no effect on BMD at the hip. Participants in DWBLF studies ranged from healthy postmenopausal women to women with diagnosed osteopenia, ranging in age from 46 to 92 years. Many of the studies included balance and flexibility exercises in addition to the DWBLF exercises. The reports of injuries and high attrition are of some concern, but injuries did occur in both control and experimental groups. The high attrition rates reported suggest that motivational strategies should be an important aspect of this type of exercise program. The exercise programs in these studies were generally high-intensity and long-duration. The overall dose ranges were 50 to 60 minutes, 3 times per week for 40 to 54 weeks at 60% to 70% of heart rate reserve.	Das dynamische, gewichtstragende Training mit geringer Krafteinwirkung verlangsamte den Knochendichteverlust an der Lendenwirbelsäule, hatte aber keinen Einfluss auf die Knochendichte an der Hüfte [33, 36, 37, 41, 42]. Die Teilnehmer an den Studien zu dynamischem, gewichtstragendem Training mit geringer Krafteinwirkung reichten von gesunden Frauen nach der Menopause bis hin zu Frauen mit diagnostizierter Osteopenie und waren zwischen 46 und 92 Jahre alt. Viele der Studien umfassten neben dem dynamischen, gewichtstragenden Training mit geringer Krafteinwirkung auch Gleichgewichts- und Flexibilitätsübungen. Die Berichte über Verletzungen und hohe Abbruchraten geben Anlass zur Sorge, doch kam es sowohl in den Kontrollgruppen als auch in den Interventionsgruppen zu Verletzungen. Die hohen Abbruchraten deuten darauf hin, dass Motivationsstrategien ein wichtiger Aspekt dieser Art von Trainingsprogramm sein sollten. Die Trainingsprogramme in diesen Studien waren im Allgemeinen hochintensiv und von langer Dauer. Die Gesamtdosis

Original English Version	German Version
	betrug 50 bis 60 Minuten, 3-mal pro Woche für 40 bis 54 Wochen bei 60 % bis 70 % der Herzfrequenzreserve.
The NWBHF exercise had a positive effect on BMD at both the spine and the femoral neck. Participants in these studies ranged from healthy postmenopausal women to women with diagnosed osteoporosis or osteopenia, ranging in age from 41 to 75 years. These studies reported a low incidence of injuries. Because older individuals may be at increased risk for falls, NWBHF exercises such as seated, high-intensity progressive resistive strength training may be inherently safer than DWBHF exercises in this population.<br>The exercise programs in these studies were generally high-intensity and long-duration. The NWBHF exercises doses ranged from 1 to 3 sets, 7 to 14 repetitions, at approximately 70% to 85% 1 repetition maximum (RM), 2 to 3 times per week, for 24 to 104 weeks. Estimation of intensity based on 1RM is beyond the scope of this CPG but has previously been addressed in other publications.	Das nicht gewichtstragende Training mit hoher Krafteinwirkung hatte einen positiven Effekt auf die Knochendichte sowohl an der Wirbelsäule als auch am Oberschenkelhals [33, 39]. Die Teilnehmenden an diesen Studien umfassen sowohl gesunde Frauen nach der Menopause als auch Frauen mit diagnostizierter Osteoporose oder Osteopenie im Alter von 41 bis 75 Jahren. Diese Studien berichteten über eine geringe Inzidenz von Verletzungen. Da ältere Menschen möglicherweise einem erhöhten Sturzrisiko ausgesetzt sind, ist in dieser Population ein nicht gewichtstragendes Training mit hoher Krafteinwirkung wie sitzendes, hochintensives progressives Krafttraining möglicherweise grundsätzlich sicherer als ein dynamisches gewichtstragendes Training mit hoher Krafteinwirkung. Die Trainingsprogramme in diesen Studien waren im Allgemeinen hochintensiv und von langer Dauer. Die Dosierung des nicht gewichtstragenden Trainings mit hoher Krafteinwirkung reichte von 1 bis 3 Sätzen, 7 bis 14 Wiederholungen, bei etwa 70 % bis 85 % 1-Wiederholungsmaximums, 2- bis 3-mal pro Woche, über 24 bis 104 Wochen. Die Schätzung der Intensität auf der Grundlage des 1-Wiederholungsmaximums war kein Fokus dieser Leitlinie, wurde aber bereits in anderen Veröffentlichungen behandelt [43].
The COMB exercise moderately improved BMD at the spine and slightly improved BMD at the trochanter/femoral neck. In each of the 4 COMB studies, references were made to aerobic exercise or low-impact aerobics, but it is not clear how these activities were performed. Therefore, based on the data provided, we recommend utilizing DWBLF paired with NWBHF. Participants in these studies included postmenopausal women with osteoporosis with ages ranging from 57 to 87 years.	Eine Kombination verschiedener Trainingsformen verbesserte die Knochendichte an der Wirbelsäule mäßig und die Knochendichte am Trochanter/Oberschenkelhals leicht [33, 37, 38, 40–42]. In jeder der 4 Studien zur Kombination verschiedener Trainingsformen wurde auf aerobes Training oder Low-Impact-Aerobics verwiesen, aber es ist nicht klar, wie diese Aktivitäten durchgeführt wurden. Daher wird auf der Grundlage der vorgelegten Daten die Anwendung von dynamischem, gewichtstragenden Training mit geringer Krafteinwirkung in Kombination mit nicht gewichtstragendem Training mit hoher Krafteinwirkung empfohlen. Die Teilnehmerinnen an diesen Studien waren Frauen nach der Menopause mit Osteoporose im Alter von 57 bis 87 Jahren.

Original English Version	German Version
There was insufficient evidence to support the use of NWBLF or DWBHF exercises. The NWBLF exercises may be underdosed on the basis of recommendations from the APTA in the American Board of Internal Medicine Foundation's "Choosing Wisely" campaign. However, it is important to note that participants in the studies examining this type of exercise were healthy postmenopausal women.<br>The effect of NWBLF exercises on osteoporotic women is unknown. The DWBHF exercises did not have a positive effect on BMD at the spine or the hip. There is a possibility that the high-frequency, high-magnitude activities, such as plyometrics, jumping, and running, included in DWBHF programs could result in stress reactions or structural failure due to excessive overload in women with osteoporosis.<br>Conversely, the inclusion of vibration as a DWBHF activity is inconsistent with practical application in the United States. This intervention is typically done in a static standing position, with frequencies ranging from 12 to 50 Hz, and with no additional external force added. Thus, overall, DWBHF exercises did not show a positive effect in the SIGN document. Based on the description of DWBHF, it is important to note that minimal injuries were reported across the 4 studies indicating that this type of exercise could be safe, but the conclusions were drawn from a relatively small number of studies utilizing this form of exercise with postmenopausal women (see Table 4).	Es gab keine ausreichende Evidenz, um nicht gewichtstragendes Training mit geringer Krafteinwirkung oder dynamisches gewichtstragendes Training mit hoher Krafteinwirkung zu empfehlen [33, 38]. Das nicht gewichtstragende Training mit geringer Krafteinwirkung könnte auf der Grundlage der Empfehlungen der APTA in der Kampagne „Choosing Wisely" der „American Board of Internal Medicine Foundation" unterdosiert sein [44]. Es ist jedoch zu beachten, dass es sich bei den Teilnehmerinnen der Studien, die diese Art von Training untersuchten, um gesunde Frauen nach der Menopause handelte. Der Effekt des nicht gewichtstragenden Trainings mit hoher Krafteinwirkung auf Frauen mit Osteoporose ist unklar. Das dynamische, gewichtstragende Training mit hoher Krafteinwirkung hatte keinen positiven Effekt auf die Knochendichte an der Wirbelsäule oder der Hüfte. Es besteht die Möglichkeit, dass die in den dynamischen, gewichtstragenden Trainingsprogrammen mit hoher Krafteinwirkung enthaltenen hochfrequenten und hochintensiven Aktivitäten wie Plyometrie, Springen und Laufen bei Frauen mit Osteoporose zu Stressreaktionen oder Verlust der strukturellen Integrität aufgrund von Überlastung führen könnten [45]. Die Einordnung von Vibrationen als Aktivitäten eines dynamischen, ewichtstragenden Trainings mit hoher Krafteinwirkung steht dagegen im Widerspruch zur praktischen Anwendung in den USA. Diese Intervention wird typischerweise in einer statischen Stehposition mit Frequenzen von 12 bis 50 Hz und ohne zusätzliche äußere Krafteinwirkung durchgeführt [46]. Daher zeigte ein dynamisches gewichtstragendes Training mit hoher Krafteinwirkung im Allgemeinen keine positive Wirkung im SIGN-Dokument. Basierend auf der Beschreibung von dynamischem, gewichtstragendem Training mit hoher Krafteinwirkung ist es wichtig zu beachten, dass in den 4 Studien nur minimale Verletzungen berichtet wurden, was darauf hindeutet, dass diese Art von Training sicher sein könnte. Allerdings wurden die Schlussfolgerungen aus einer relativ kleinen Anzahl von Studien gezogen, die diese Form des Trainings bei Frauen nach der Menopause einsetzten (siehe Tabelle 4).
**SIGN EXERCISE RECOMMENDATIONS FOR PREMENOPAUSAL WOMEN**	**SIGN TRAININGSEMPFEHLUNGEN FÜR FRAUEN VOR DER MENOPAUSE**
Static Weight-Bearing Exercise<br>*SIGN recommendation*: No evidence-based recommendation provided.<br>*SIGN summary*: No systematic reviews have provided evidence on SWB forms of exercise intervention in premenopausal women.	Statisches gewichtstragendes Training<br>SIGN-Empfehlung: Keine evidenzbasierte Empfehlung möglich.<br>SIGN-Zusammenfassung: Es gibt keine systematischen Reviews, die Evidenz für Trainingsinterventionen mit Formen des statischen, gewichtstragenden Trainings bei Frauen vor der Menopause bieten.

Original English Version	German Version
Dynamic Weight-Bearing Exercise (Low Force)<br>*SIGN recommendation*: No evidence-based recommendation provided.<br>*SIGN summary*: No systematic reviews have provided evidence on DWBLF forms of exercise intervention in premenopausal women. One meta-analysis of 10 RCTs on the effect of walking on BMD in postmenopausal and perimenopausal women reported only a single small trial, which included perimenopausal women aged 40 to 60 years (n = 50). This study showed no effect of walking on BMD in this cohort.	Dynamisches gewichtstragendes Training (geringe Krafteinwirkung)<br>SIGN-Empfehlung: Keine evidenzbasierte Empfehlung möglich.<br>SIGN-Zusammenfassung: Es gibt keine systematischen Reviews, die Evidenz für Trainingsinterventionen mit Formen des dynamischen, gewichtstragenden Trainings bei Frauen vor der Menopause liefern. Eine Meta-Analyse von 10 RCTs über die Wirkung von Gehen auf die Knochendichte bei Frauen vor und nach der Menopause berichtete nur über eine einzige kleine Studie, die Frauen vor und nach der Menopause im Alter von 40 bis 60 Jahren einschloss (n = 50). Diese Studie zeigte keinen Effekt des Gehens auf die Knochendichte in dieser Kohorte [47].
Dynamic Weight-Bearing Exercise (High Force)<br>*SIGN recommendation*: Progressive-resistance strength training (such as closed kinetic chain weight training) alone, or in combination with impact exercise (such as stair climbing or jogging), should be considered to slow the decline of lumbar spine BMD.<br>*SIGN summary*: High-impact only programs were effective in reducing BMD decline only at the femoral neck (weighted mean difference, WMD: 0.024 g/cm^2; 95% CI, 0.002-0.027; $P < .00001$).	Dynamisches gewichtstragendes Training (hohe Krafteinwirkung)<br>SIGN-Empfehlung: Progressives Krafttraining (z. B. Krafttraining in geschlossener kinetischer Kette) allein oder in Kombination mit Impact-Training (z. B. Treppensteigen oder Joggen) sollte in Betracht gezogen werden, um den Rückgang der Knochendichte der Lendenwirbelsäule zu verlangsamen.<br>SIGN-Zusammenfassung: Programme, die ausschließlich "high-impact" Training beinhalteten, waren bei der Verringerung des Knochendichte-Rückgangs nur am Oberschenkelhals wirksam (gewichteter mittlerer Unterschied: 0,024 g/cm2; 95% CI, 0,002-0,027; P < 0,00001) [48].
Non–Weight-Bearing Exercise (Low Force)<br>*SIGN recommendation*: No evidence-based recommendation provided.<br>*SIGN summary*: No systematic reviews have provided evidence on NWBLF forms of exercise intervention in premenopausal women.	Nicht gewichtstragendes Training (geringe Krafteinwirkung)<br>SIGN-Empfehlung: Keine evidenzbasierte Empfehlung möglich.<br>SIGN-Zusammenfassung: Es gibt keine systematischen Reviews, die Evidenz für Trainingsinterventionen mit Formen von nicht gewichtstragendem Training mit geringer Krafteinwirkung bei Frauen vor der Menopause berichten.
Non–Weight-Bearing Exercise (High Force)<br>*SIGN recommendation*: Progressive-resistance strength training (such as open kinetic chain weight training) should be considered to slow the decline of lumbar spine BMD.<br>*SIGN summary*: High-intensity progressive resistance training was shown to be effective in increasing absolute BMD at the lumbar spine (WMD: 0.014 g/cm^2; 95% CI, 0.009-0.019; $P < .00001$) but not the femoral neck (WMD: 0.001 g/cm^2; 95% CI, −0.006 to 0.008, $P = .78$) in premenopausal women.	Nicht gewichtstragendes Training (hohe Krafteinwirkung)<br>SIGN-Empfehlung: Progressives Krafttraining (wie z. B. in offener kinetischer Kette) sollte in Betracht gezogen werden, um den Rückgang der Knochendichte der Lendenwirbelsäule zu verlangsamen.<br>SIGN-Zusammenfassung: Hochintensives progressives Krafttraining erwies sich als wirksam bei der Erhöhung der absoluten Knochendichte der Lendenwirbelsäule (gewichteter mittlerer Unterschied: 0,014 g/cm2; 95% CI, 0,009-0,019; P < 0,00001), nicht aber am Oberschenkelhals (gewichteter mittlerer Unterschied: 0,001 g/cm2; 95% CI, -0,006 bis 0,008, P = 0,78) bei Frauen vor der Menopause [49].

Original English Version	German Version
Combination of Exercise Types<br>*SIGN recommendation*: High-impact exercise (such as jogging and stair climbing) and combining impact exercise with progressive-resistance strength training should be considered to slow the decline of femoral neck and lumbar BMD.<br>*SIGN summary*: Exercise programs that combine odd- or high-impact activity with high-magnitude resistance training appear effective in increasing BMD in premenopausal women at the femoral neck (WMD: 0.007 g/cm^2; 95% CI, 0.001-0.013; *P* = .02) and the spine (WMD: 0.009 g/cm^2; 95% CI, 0.002-0.015; *P* = .01).	Kombination von Trainingsformen<br>SIGN-Empfehlung: Um den Rückgang der Knochendichte im Oberschenkelhals und in der Lendenwirbelsäule zu verlangsamen, sollte ein Trainingsprogramm mit hohem Impact (z. B. Joggen und Treppensteigen) und eine Kombination aus Impact-Training und progressivem Krafttraining in Betracht gezogen werden.<br>SIGN-Zusammenfassung: Trainingsprogramme, die „odd-impact" oder „high-impact" Belastungen mit einem Krafttraining mit hohem Bewegungsausmaß kombinieren, scheinen die Knochendichte bei Frauen vor der Menopause am Oberschenkelhals (gewichteter mittlerer Unterschied: 0,007 g/cm2; 95% CI, 0,001-0,013; P = 0,02) und an der Wirbelsäule (gewichteter mittlerer Unterschied: 0,009 g/cm2; 95% CI, 0,002-0,015; P = 0,01) zu erhöhen [48].
**ADAPTED EXERCISE RECOMMENDATIONS FOR PREMENOPAUSAL WOMEN**	**ADAPTIERTE TRAININGSEMPFEHLUNGEN FÜR FRAUEN VOR DER MENOPAUSE**
Exercise Recommendations for Premenopausal Women to Slow Decline of BMD of the Femoral Neck<br>Physical therapists should consider designing and advising premenopausal women to participate in long-duration exercise programs consisting of high-impact exercise (such as jogging) and combining impact exercises (such as stair climbing) with progressive-resistance strength training (such as weight training) to slow the decline of femoral neck BMD. (Grade B, recommendation based on moderate evidence)	Trainingsempfehlungen für Frauen vor der Menopause zur Verlangsamung des Rückgangs der Knochendichte am Oberschenkelhals<br>Physiotherapeut:innen sollten in Erwägung ziehen, für Frauen vor der Menopause Langzeit-Trainingsprogramme zusammenzustellen und anzuleiten, die aus "high-impact" Training (z. B. Joggen) und einer Kombination von Impact-Training (z. B. Treppensteigen) mit progressivem Krafttraining (z. B. Krafttraining mit Gewichten) bestehen, um den Rückgang der Schenkelhals-Knochendichte zu verlangsamen. (Grad B, Empfehlung basierend auf moderater Evidenz)
Exercise Recommendations for Premenopausal Women to Slow Decline of BMD of the Lumbar Spine<br>Physical therapists should consider designing and advising premenopausal women to participate in long-duration exercise programs consisting of progressive-resistance strength training (such as weight training) alone or in combination with impact exercises (such as stair climbing or jogging) to slow the decline of lumbar spine BMD. (Grade B, recommendation based on moderate evidence)	Trainingsempfehlungen für Frauen vor der Menopause zur Verlangsamung des Rückgangs der Knochendichte der Lendenwirbelsäule<br>Physiotherapeut:innen sollten in Erwägung ziehen, für Frauen vor der Menopause Langzeit-Trainingsprogramme zusammenzustellen und anzuleiten, die aus progressivem Krafttraining (z. B. Krafttraining mit Gewichten) allein oder in Kombination mit Impact-Training (z. B. Treppensteigen oder Joggen) bestehen, um den Rückgang der Lendenwirbelsäulen-Knochendichte zu verlangsamen. (Grad B, Empfehlung basierend auf moderater Evidenz)
Premenopausal Women: Adapted Summary Statement<br>There is insufficient or no evidence on which to make recommendations about the use of SWB, DWBLF, and NWBLF exercises in premenopausal women. Evidence supported the positive impact of DWBHF such as jumping, jogging, and running in reducing BMD decline at the femoral neck. Participants in these studies were healthy, premenopausal women aged 20 to 48 years with program durations ranging from 6 months to 2 years.	Frauen vor der Menopause: Adaptierte Zusammenfassung<br>Es gibt unzureichende oder keine Evidenz, um Empfehlungen zur Verwendung von statischem, gewichtstragendem Training, dynamischem, gewichtstragendem Training mit geringer Krafteinwirkung und nicht gewichtstragendem Training mit geringer Krafteinwirkung bei Frauen vor der Menopause auszusprechen [47]. Die Evidenz stützte die positive Auswirkung von dynamischem gewichtstragendem Training mit hoher Krafteinwirkung wie Springen, Joggen und Laufen auf die Reduzierung des Knochendichte-Rückgangs am Oberschenkelhals [48]. Die

Original English Version	German Version
	Teilnehmerinnen an diesen Studien mit einer Programmdauer von 6 Monaten bis 2 Jahren waren gesunde Frauen vor der Menopause im Alter von 20 bis 48 Jahren.
Evidence also supported NWBHF exercises such as seated progressive resistive and high-load strength training for increasing BMD at the lumbar spine. Participants in these studies were healthy, premenopausal women aged 20 to 50 years with program durations ranging from 5 to 18 months. The COMB exercise programs that included combinations such as circuit strength training and high-impact aerobics or skipping and strength training increased BMD at both the femoral neck and lumbar spine in healthy participants with ages ranging from 24 to 41 years with program durations of 6 to 24 months.	Die Evidenz stützte auch nicht gewichtstragendes Training mit hoher Krafteinwirkung wie sitzendes, progressives Krafttraining und Krafttraining mit hohem Widerstand zur Steigerung der Knochendichte an der Lendenwirbelsäule [49]. Die Teilnehmenden an diesen Studien mit einer Programmdauer von 5 bis 18 Monaten waren gesunde Frauen vor der Menopause im Alter von 20 bis 50 Jahren. Trainingsprogramme mit einer Kombination verschiedener Trainingsformen, wie Zirkel-Krafttraining und „high-impact"-Aerobic oder Hüpfen und Krafttraining, erhöhten die Knochendichte sowohl am Oberschenkelhals als auch an der Lendenwirbelsäule bei gesunden Teilnehmenden im Alter von 24 bis 41 Jahren mit einer Programmdauer von 6 bis 24 Monaten [48].
These findings suggest that long-duration appropriately dosed high-force exercises are required to improve bone health in healthy premenopausal women. These programs must be viewed as preventative in that none of the participants were diagnosed with osteoporosis (see Table 5).	Diese Ergebnisse deuten darauf hin, dass lange andauerndes, angemessen dosiertes Training mit hoher Krafteinwirkung erforderlich ist, um die Knochengesundheit bei gesunden Frauen vor der Menopause zu verbessern. Diese Programme müssen als präventiv angesehen werden, da bei keiner der Teilnehmerinnen Osteoporose diagnostiziert wurde (siehe Tabelle 5).
**SIGN EXERCISE RECOMMENDATIONS FOR MEN**	**SIGN TRAININGSEMPFEHLUNGEN FÜR MÄNNER**
SIGN summary: One meta-analysis identified 3 studies that investigated the effects of exercise on BMD in men. These studies included diverse populations, with varied exercise types and used different measures of BMD. Study quality was unclear overall, and 2 studies were unpublished dissertations.<br>The primary outcome measures of change in lumbar spine or femoral neck BMD were calculated as standardized effect sizes (*g*). The *g* statistic for each group from each study was calculated as the change score difference (absolute or relative) in the exercise group minus the change score difference in the control group, divided by the pooled standard deviation of the exercise and control groups. The relative magnitude of *g* may be described as trivial (<0.20), small (≥0.20 to <0.50), medium (≥0.50 to <0.80), or large (≥0.80).	SIGN-Zusammenfassung: In einer Meta-Analyse wurden 3 Studien identifiziert, die die Auswirkungen von Training auf die Knochendichte bei Männern untersuchten. Diese Studien umfassten verschiedene Populationen mit unterschiedlichen Trainingsformen und verwendeten unterschiedliche Knochendichte -Messungen. Die Qualität der Studien war insgesamt unklar, und bei 2 Studien handelte es sich um unveröffentlichte Dissertationen. Die primären Ergebnisgrößen der Veränderung der Knochendichte der Lendenwirbelsäule oder des Schenkelhalses wurden als standardisierte Effektgrößen (g) berechnet. Die g-Statistik für jede Gruppe aus jeder Studie wurde berechnet als die Differenz der Veränderungswerte (absolut oder relativ) in der Trainingsgruppe minus der Differenz der Veränderungswerte in der Kontrollgruppe, geteilt durch die gepoolte Standardabweichung der Trainings- und Kontrollgruppen. Die relative Größe von g kann als trivial (<0,20), klein (≥0,20 bis <0,50), mittel (≥0,50 bis <0,80) oder groß (≥0,80) beschrieben werden.

Original English Version	German Version
Overall, a moderate and statistically significant benefit of exercise on the femoral neck BMD was observed (g = 0.583; 95% CI, 0.031-1.135). No significant effect was seen with exercise on lumbar spine BMD (g = 0.190; 95% CI, −0.036 to 0.416).	Insgesamt wurde ein moderater und statistisch signifikanter Nutzen von Training auf die Oberschenkelhals-Knochendichte beobachtet (g = 0,583; 95% KI, 0,031-1,135). Es wurde kein signifikanter Effekt von Training auf die Lendenwirbelsäulen-Knochendichte festgestellt (g = 0,190; 95% KI, -0,036 bis 0,416) [50].
Another systematic review considered the effect of resistance training (eg, weight training) only or in combination with impact-loading (weight bearing) activities. This small review considered heterogeneous study designs of varying and limited quality that used different sites to measure BMD. The authors concluded that exercise may be a safe and effective means to reduce BMD loss in middle- and older-aged men. Exercise protocols included a range of DWBLF, DWBHF, NWBLF, and NWBHF activities.	Ein weiteres systematisches Review befasste sich mit der Wirkung von Krafttraining (z. B. mit Gewichten) allein oder in Kombination mit stoßbelastenden (gewichtstragenden) Aktivitäten. Dieses kleine Review berücksichtigte heterogene Studiendesigns von unterschiedlicher und begrenzter Qualität, die an verschiedenen Körperstellen zur Messung der Knochendichte durchgeführt wurden. Die Autor:innen kamen zu dem Schluss, dass Training ein sicheres und wirksames Mittel zur Verringerung des Knochendichte-Verlusts bei Männern mittleren und höheren Alters sein kann [51]. Die Trainingsprotokolle umfassten eine Reihe von Aktivitäten des dynamischen, gewichtstragenden Trainings mit geringer Krafteinwirkung, dynamischem, gewichtstragendem Training mit hoher Krafteinwirkung, nicht gewichtstragendem Training mit geringer Krafteinwirkung und nicht gewichtstragendem Training mit hoher Krafteinwirkung.
SIGN recommendation: There is currently limited evidence on the role of exercise in mitigating BMD loss in men. Further well-designed research studies in men are needed before any recommendations can be made.	SIGN-Empfehlung: Derzeit gibt es nur begrenzte Evidenz über die Rolle von Training, um den Knochendichte-Verlust bei Männern zu verringern. Bevor Empfehlungen ausgesprochen werden können, sind weitere hochwertige Studien mit Männern erforderlich.
**ADAPTED EXERCISE RECOMMENDATIONS FOR MEN**	**ADAPTIERTE TRAININGSEMPFEHLUNGEN FÜR MÄNNER**
Men: Adapted Summary Statement<br>There is insufficient evidence to support exercise as a factor in improving BMD in men. However, exercise is a safe and effective means of maintaining and improving health, function, and QOL supported by national physical activity guidelines.<br>Because of a lack of research evidence on the role of exercise in improving BMD in men, we were unable to provide any adapted exercise recommendations for men.	Männer: Adaptierte Zusammenfassung<br>Es gibt unzureichende Evidenz, dass Training bei Männern zur Verbesserung der Knochendichte beiträgt. Training ist jedoch ein sicheres und wirksames Mittel zur Erhaltung und Verbesserung der Gesundheit, der Funktionsfähigkeit und der Lebensqualität, das durch nationale Leitlinien für Bewegung unterstützt wird [52].<br>Aufgrund fehlender Forschungsergebnisse zur Rolle von Bewegung bei der Verbesserung der Knochendichte bei Männern konnten keine adaptierten Trainingsempfehlungen für Männer gegeben werden.
**DISCUSSION**	**DISKUSSION**
**SIGN Summary of Knowledge Gaps and Future Research**	**SIGN-Zusammenfassung der Wissenslücken und Forschungsbedarf**
Overall, there was a small, positive effect of exercise on BMD in postmenopausal women that is exercise-type and site-specific. High-force exercise can reduce BMD decline at the femoral neck, progressive-resistance exercise can reduce BMD decline at the lumbar spine, and impact protocols combined with resistance can reduce BMD decline at both the femoral neck	Insgesamt gab es bei Frauen nach der Menopause einen geringen positiven Effekt von Training auf die Knochendichte, der spezifisch für die Trainingsform- und Körperregion ist. Krafttraining kann den Rückgang der Knochendichte am Oberschenkelhals verringern, progressives Krafttraining kann den Rückgang der Knochendichte an der Lendenwirbelsäule verringern und

Original English Version	German Version
and the lumbar spine. When comparing premenopausal with postmenopausal recommendations, there is a change in loading recommendations.	Trainingsprotokolle mit einer Kombination aus Impact- und Krafttraining können den Rückgang der Knochendichte sowohl am Oberschenkelhals als auch an der Lendenwirbelsäule verringern.
Premenopausal women should perform high-force exercises in both weight-bearing and non–weight bearing positions to improve hip and spine BMD. This exercise prescription is aligned with current recommendations to improve bone size and strength, which may translate to a reduction in fractures. When transitioning to postmenopausal women, high forces are recommended only for non–weight-bearing (ie, seated, supported) positions, while low forces should be used with static (unilateral stance) or dynamic (walking, dancing) exercises.	Beim Vergleich der Empfehlungen für Frauen vor und nach der Menopause gibt es eine Änderung bei den Empfehlungen bzgl. der Krafteinwirkung. Frauen vor der Menopause sollten Training mit hoher Krafteinwirkung sowohl in gewichtstragenden als auch in nicht gewichtstragenden Positionen durchführen, um die Knochendichte der Hüfte und der Wirbelsäule zu verbessern. Diese Trainingsempfehlung entspricht den aktuellen Empfehlungen zur Verbesserung der Knochengröße und -stärke, was zu einer Verringerung von Frakturen führen kann. Bei Frauen nach der Menopause werden hohe Krafteinwirkungen nur für nicht gewichtstragende (d. h. sitzende, unterstützte) Positionen empfohlen, während bei statischem (Einbeinstand) oder dynamischem Training (Gehen, Tanzen) niedrige Krafteinwirkungen verwendet werden sollten.
Although there was no specific recommendation for men, the lack of adverse effects supported our recommendation of encouraging and implementing lifelong fitness and bone health strategies such as those recommended for women of corresponding ages. Research studies commonly focus on the hip, spine, and radius because these sites make up the large majority of fracture sites. While we cannot speak to BMD improvements at additional sites, we cannot exclude the possibility that these recommendations can have universal skeletal effects. Conclusions must be interpreted with some caution as the original studies suffered from diverse methodological and reporting discrepancies that resulted in low study quality.	Obwohl es keine spezifischen Empfehlungen für Männer gab, unterstützte das Fehlen von Nebenwirkungen diese Empfehlung, Strategien für lebenslange Fitness und Knochengesundheit zu fördern und umzusetzen, in Anlehnung an die Empfehlungen für Frauen. Die Studien konzentrieren sich in der Regel auf Hüfte, Wirbelsäule und Radius, da diese Stellen die große Mehrheit der Frakturstellen ausmachen. Obwohl keine Aussagen zu Verbesserungen der Knochendichte an anderen Stellen getroffen werden können, kann nicht ausgeschlossen werden, dass diese Empfehlungen universelle Auswirkungen auf das Skelett haben können. Die Schlussfolgerungen müssen mit einer gewissen Vorsicht interpretiert werden, da die ursprünglichen Studien unter verschiedenen methodischen und berichtstechnischen Limitationen litten, die zu einer geringen Studienqualität führten [21].
Osteoporosis is difficult to capture in a silo of simply BMD. While BMD is important, fracture risk and falling are as equally important to the patient for QOL and mobility. The difficulty lies in how to identify change and utilize palatable evidence-based documents for clinicians and patients. We viewed osteoporotic fractures as a combination of low BMD and fall risk. Given that a falls evidence-based document specific to physical therapy currently exists, our focus for this document was solely on BMD.	Osteoporose lässt sich nur schwer allein durch die Messung die Knochendichte erfassen. Die Knochendichte ist zwar wichtig, aber das Frakturrisiko und Stürze sind für die Lebensqualität und Mobilität der Patient:innen ebenso wichtig. Die Schwierigkeit für Kliniker:innen und Patient:innen besteht darin, Veränderungen zu erkennen und verständliche, evidenzbasierte Dokumente zu nutzen. Das APTA-Leitlinien-Team betrachtet osteoporotische Frakturen als eine Kombination aus niedriger Knochendichte und Sturzrisiko. Da es bereits ein evidenzbasiertes Dokument speziell für die Physiotherapie bei Stürzen gibt, lag der Schwerpunkt bei diesem Dokument ausschließlich auf der Knochendichte.
The challenge specific to BMD is the long duration needed to improve bone health parameters, paired with the clinical need to document BMD changes that we as physical therapists do not measure. The lack of head-to-head comparisons of interventions and	Die Herausforderung bei der Behandlung von Osteoporose besteht in der langen Zeit, die benötigt wird, um die Knochengesundheit zu verbessern, und in der Schwierigkeit, Veränderungen der Knochendichte zu dokumentieren, die von Physiotherapeut:innen gar nicht

Original English Version	German Version
outcomes increase the complexity in decision making for clinicians and for patients to realize progress. Regardless of expected outcomes, properly dosed exercise is assumed to be a safe intervention, with appropriate considerations for patients who present to the physical therapist with previous fracture, injuries, or comorbidities in combination with osteoporosis.	gemessen werden. Das Fehlen direkter Vergleiche von Interventionen und Outcomes erschwert die Entscheidungsfindung für Kliniker:innen und Patient:innen. Unabhängig von den erwarteten Ergebnissen wird richtig dosiertes Training als sichere Intervention angesehen, wobei Anpassungen in der physiotherapeutischen Behandlung für Patient:innen mit früheren Frakturen, Verletzungen oder Komorbiditäten in Kombination mit Osteoporose getroffen werden müssen.
**Adapted Summary of Knowledge Gaps and Future Research**	**Adaptierte Zusammenfassung der Wissenslücken und zukünftigen Forschung**
Evidence concerning the safety of exercise interventions was inferred from reports of very few adverse events in trials of exercise intervention. Older individuals have both real and perceived concerns about participating in long-duration exercise programs. Unfortunately, none of the research studies supporting this CPG specifically examined exercise safety or included participants who would be at high risk for adverse exercise outcomes. The lack of evidence should not infer to the reader that exercise is unsafe for older individuals attempting to improve BMD but rather, further research is warranted particularly in high-risk populations.	Die Evidenz für die Sicherheit von Trainingsinterventionen wurde aus Berichten über sehr wenige unerwünschte Ereignisse in Studien über Trainingsinterventionen abgeleitet. Ältere Menschen haben sowohl objektiv feststellbare als auch subjektiv wahrgenommene Bedenken bezüglich der Teilnahme an langfristigen Trainingsprogrammen. Keine der Studien, die diese Leitlinie unterstützen, hat die Sicherheit von Training spezifisch untersucht oder Teilnehmende eingeschlossen, die ein hohes Risiko für unerwünschte Trainingsergebnisse aufweisen. Das Fehlen von Evidenz sollte die Lesenden nicht zu der Annahme verleiten, dass Training für ältere Menschen, die versuchen, die Knochendichte zu verbessern, unsicher ist, sondern vielmehr, dass weitere Forschung, insbesondere in Hochrisikopopulationen, erforderlich ist.
Both clinicians and patients want to identify the best exercise program to improve BMD. Unfortunately, none of the studies directly compared 2 potentially effective exercise interventions. Such studies would be very costly and difficult to conduct. One of the challenges of bone research is that in adults, bone does not demonstrate appreciable and measurable short-term (ie, <3 months) adaptations in response to loading, rather bone adapts over the long term (ie, 6-48+ months).	Sowohl Kliniker:innen als auch Patient:innen möchten das beste Trainingsprogramm zur Verbesserung der Knochendichte ermitteln. In keiner der Studien wurden zwei potenziell wirksame Trainingsinterventionen direkt miteinander verglichen. Solche Studien würden mit hohen Kosten und Schwierigkeiten in der Durchführung einhergehen. Eine der Herausforderungen der Knochenforschung besteht darin, dass bei Erwachsenen der Knochen keine nennenswerten und messbaren kurzfristigen (d. h. <3 Monate) Anpassungen als Reaktion auf Belastung zeigt, sondern sich der Knochen langfristig anpasst (d. h. 6-48+ Monate) [45].
Studies comparing 2 exercise interventions for their impact on hip and spine BMD would need to be of long duration. The long duration combined with the large sample size required to compare the effectiveness of 2 interventions would present several challenges including the high cost of such a study, difficulty recruiting participants, and maintaining exercise adherence over a long period. Exercise intervention studies to improve BMD of nonosteoporotic premenopausal women would have the added problem of focusing on preventing rather than treating osteoporosis.	Studien, die zwei Trainingsinterventionen hinsichtlich ihrer Auswirkungen auf die Knochendichte der Hüfte und Wirbelsäule vergleichen, müssten über einen langen Zeitraum durchgeführt werden. Die lange Dauer in Kombination mit der großen Stichprobengröße, die für den Vergleich der Wirksamkeit zweier Interventionen erforderlich ist, würde zahlreiche Herausforderungen mit sich bringen, darunter die hohen Kosten einer solchen Studie, die Schwierigkeit, Teilnehmende zu rekrutieren, und die Aufrechterhaltung der Trainingsadhärenz über einen langen Zeitraum. Studien zu Trainingsinterventionen zur Verbesserung der Knochendichte bei Frauen ohne Osteoporose vor der Menopause hätten das zusätzliche Problem, dass der Schwerpunkt eher auf der Vorbeugung als auf der Behandlung von Osteoporose liegt.
In addition, these studies lacked racial diversity and therefore it is difficult to extrapolate findings to the general US population. Given all these obstacles, it is not surprising that we	Außerdem waren diese Studien nicht nach ethnischer Zugehörigkeit aufgeschlüsselt, sodass es schwierig ist, die Ergebnisse auf die allgemeine US-Bevölkerung zu übertragen. Angesichts all

Original English Version	German Version
continue to lack evidence to guide physical therapist management of individuals with osteoporosis. Future studies should explore response to exercise interventions targeted to individuals across broader racial groups. Physical therapist management of osteoporosis from examination and evaluation to intervention will be addressed in a companion document that utilized a Delphi process to produce recommendations based on expert opinion.	dieser Hindernisse ist es nicht überraschend, dass weiterhin Evidenz fehlt, die Physiotherapeut:innen bei der Behandlung von Menschen mit Osteoporose unterstützt. Zukünftige Studien sollten die Reaktion auf Trainingsinterventionen untersuchen, die auf Personen aus breiteren ethnischen Gruppen abzielen. Das Behandlungsmanagement von Osteoporose durch Physiotherapeut:innen von der Untersuchung und Befundung bis zur Intervention wird in einem Begleitdokument behandelt, das einen Delphi-Prozess zur Erstellung von Empfehlungen auf der Grundlage von Expert:innenmeinungen verwendet.
Although fracture and falls were not a focus of this document, we retained the full spirit of the SIGN recommendation. There was evidence that exercise influences fracture risk when the exercise is multimodal and part of a fall prevention program. No systematic reviews have reported on fracture/falls risk, adverse effects, or QOL outcomes of exercise interventions for premenopausal women with osteoporosis. Fractures are an important outcome measure in individuals with osteoporosis and are closely associated with fall risk.	Obwohl Frakturen und Stürze nicht im Mittelpunkt dieses Dokuments standen, wurde der Sinn und Zweck der SIGN-Empfehlung beibehalten. Es gab Belege dafür, dass Training das Frakturrisiko beeinflusst, wenn das Training multimodal und Teil eines Sturzpräventionsprogramms ist. Es gibt keine systematischen Reviews, die über das Fraktur-/Sturzrisiko, unerwünschte Wirkungen oder die Auswirkungen auf die Lebensqualität von Trainingsinterventionen bei Frauen mit Osteoporose vor der Menopause berichten. Frakturen sind ein wichtiger Endpunkt bei Personen mit Osteoporose und stehen in engem Zusammenhang mit dem Sturzrisiko.
Exercise interventions may decrease the risk of fracture by both increasing BMD and by decreasing risk of falling. It is important for physical therapists to assess the risk of falling in individuals with osteoporosis, which is not addressed in this document. We recommend that clinicians managing patients with osteoporosis consult the Academy of Geriatric Physical Therapy/APTA clinical guidance statement directed at fall risk reduction.	Trainingsinterventionen können das Frakturrisiko senken, indem sie sowohl die Knochendichte erhöhen als auch das Sturzrisiko verringern. Physiotherapeut:innen müssen unbedingt das Sturzrisiko von Personen mit Osteoporose beurteilen, was in diesem Dokument nicht behandelt wird. Die APTA-Leitlinien-Gruppe empfiehlt Kliniker:innen, die Patient:innen mit Osteoporose betreuen, die Leitlinie der „Academy of Geriatric Physical Therapy/APTA“ zur Reduzierung des Sturzrisikos zu konsultieren [32].
---	Für die Versorgung im deutschsprachigen Raum stehen die Empfehlungen für das körperliche Training zur Sturzprävention für ältere, zuhause lebende Menschen der Bundesinitiative Sturzprävention [53, 54] zur Verfügung.
**Implementation**	**Implementierung**
---	Kommentar zur deutschen Version der Leitlinie: In der englischen Originalversion der Leitlinie sind Hinweise zur Implementierung der Leitlinie in den USA formuliert. Diese Hinweise sind teilweise nicht auf den Kontext in Deutschland, Österreich bzw. der Schweiz übertragbar. Daher wurde von einer Übersetzung dieses Kapitels der Leitlinie abgesehen.
The recommendations included in this document are supported by evidence indicating specific types of exercise that may improve or slow the decline in BMD at the hip and the lumbar spine in both pre- and postmenopausal women. However, it is important to recognize that this evidence comes from long-duration exercise programs ranging in length from many	---

Original English Version	German Version
months to a year or more. In the United States, the duration of physical therapy episodes of physical therapist care is not commonly sustained for a year or more.	
However, patients could be managed for much longer episodes of care throughout the life span by participating in long-duration exercise programs and routine/annual physical therapy checkups. For patients with access to Medicare, physical therapists' services are a covered benefit, with no limitations on care (related to time, visits, or cost) if the services provided are medically necessary and skilled. This includes maintenance care and care to prevent a decline in function.	---
A new model for physical therapy management of patients with osteoporosis could involve concluding and then resuming multiple short episodes of care over an extended time period. In addition, patients who are able to safely participate in lifelong physical activities to improve bone health, and health in general, should be encouraged to do so.	---
The ability of individuals to follow physical therapy recommendations requiring them to participate in long-duration exercise programs will be influenced by many of the social determinants of health. *Healthy People 2020* organizes the social determinants of health around 5 key domains: (1) economic stability, (2) education, (3) health and health care, (4) neighborhood and built environment, and (5) social and community context.	---
Physical therapists should evaluate each patient's social determinants of health when designing long-duration exercise programs to improve bone health in premenopausal women and maintain bone mass in women with osteoporosis. We recommend that clinicians consider the following 5 factors:	---
**Economic stability**	**---**
Patients in economic distress may be food or housing insecure. Such patients would be unable to join a health club or purchase exercise equipment. Fortunately, many exercises do not require equipment. Static weight-bearing exercises such as single-leg stance may slow the decline of hip BMD if performed at least 1 minute per leg.	---
When individuals cannot join a health club or purchase exercise equipment, NWBHF (seated progressive resistive, high-load strength training) programs can use easily accessible items such as elastic tubing or plastic jugs filled with water to provide resistance. The DWBLF walking programs to slow decline in spine BMD in postmenopausal women with osteoporosis or DWBHF programs to prevent osteoporosis (ie, premenopausal women) such as running, jogging, jumping, or stair climbing do not require equipment or access to a health club.	---

Original English Version	German Version
**Education**	**---**
Patients with marginal literacy may have difficulty understanding written instructions for home exercise programs. Patients with poor literacy may not understand the instructions to progress exercise programs, exercise precautions, or appreciate the benefits of long-duration exercise. This type of patient may require closer supervision, video instruction, or illustrated handouts provided by a physical therapist.	---
**Health and health care**	**---**
Patients without health insurance may not be able to afford even a small number of physical therapy sessions to develop and progress an exercise program. Patients covered by Medicare advantage plans may have limitations on the number of therapy sessions allowed for an episode of care. Patients with traditional Medicare Part B may have more flexibility in the number and duration of an episode of care for licensed therapists to prescribe and monitor an exercise program to maintain BMD. Patients with poor health literacy may not understand the relationship between exercise and BMD and may require closer supervision by a physical therapist.	---
**Neighborhood and built environment**	**---**
Exercise programs involving walking or jogging may slow the decline of BMD of the femoral neck and lumbar spine. However, patients who live in dangerous neighborhoods due to crime or in neighborhoods where sidewalks are lacking or in disrepair may have difficulty participating in these types of exercise programs. Physical therapists should consider the patient's physical environment when designing exercise programs and may recommend possible alternative environments such as parks, malls, or school grounds when available.	---
**Social and community**	**---**
Exercise programs to improve or maintain BMD must be of long duration to be effective. The duration can range from 6 to 48 months, while the most commonly employed time frame is 12 months. Social support and encouragement from family or friends are often critical in initiating and sustaining participation. Individuals who live alone or who are socially isolated may benefit from joining health clubs or community exercise groups.	---
**External Review**	**Externe Begutachtung**
**Expert reviewers**	**Experten-Gutachtende**
A panel of 17 experts in geriatric physical therapy and/or osteoporosis was invited to review an initial draft of the document. Invited experts included 3 physical therapists with	Eine Gruppe von 17 Expert:innen für geriatrische Physiotherapie und/oder Osteoporose wurde eingeladen, einen ersten Entwurf der original APTA-Leitlinie zu überprüfen. Zu den eingeladenen

Original English Version	German Version
demonstrable research expertise in physical therapist management of patient/clients with osteoporosis, 8 physicians (endocrinology, geriatrics, and/or orthopedics), 3 exercise physiologists with research expertise in exercise and bone health, and 2 lay people who would be impacted by the work. The panelists were provided with a draft of the work and then asked to complete a 22-item survey asking about its methodology and feasibility of implementation.	Expert:innen gehörten 3 Physiotherapeut:innen mit nachweislicher Forschungserfahrung in der physiotherapeutischen Behandlung von Patient:innen/Klient:innen mit Osteoporose, 8 Ärztinnen und Ärzte (Endokrinologie, Geriatrie und/oder Orthopädie), 3 Sportphysiolog:innen mit Forschungserfahrung in den Bereichen Training und Knochengesundheit sowie 2 Laien. Die Expert:innen erhielten einen Entwurf der Leitlinie und wurden gebeten, einen Fragebogen mit 22 Fragen zur Methodik und Umsetzbarkeit auszufüllen.
Four of the invited experts commented (2 physical therapists and 2 exercise physiologists). Panelists were given up to 30 days to provide input. Detailed results of the survey are presented in Supplemental Digital Content Table 1, available at: https://links.lww.com/JGPT/A104, which summarizes invited expert and APTA Geriatrics Bone Health Special Interest Group members' levels of agreement. All comments, suggestions, and feedback from the expert reviewers were provided to the authors for consideration and revisions. Revisions to the draft were made in response to relevant comments.	Vier der eingeladenen Expert:innen (zwei Physiotherapeut:innen und zwei Sportphysiolog:innen) gaben Kommentare ab. Die Expert:innen hatten 30 Tage Zeit, um ein Feedback zu geben. Die detaillierten Ergebnisse der Befragung sind in Tabelle 1 des digitalen Zusatzmaterials aufgeführt, die unter https://links.lww.com/JGPT/A104 verfügbar ist und die den Grad der Zustimmung der eingeladenen Expert:innen und der Mitglieder der „APTA Geriatrics Bone Health Special Interest Group" zusammenfasst. Alle Kommentare, Vorschläge und Rückmeldungen der Expert:innen wurden den Autor:innen zur Prüfung und Überarbeitung vorgelegt. Der Entwurf der Leitlinie wurde auf der Grundlage der relevanten Kommentare überarbeitet.
**Target user reviewers**	**Begutachtung durch die Zielgruppe der Leitlinie**
Members of APTA Geriatrics were invited to review and comment on the draft via a blast email. Additional targeted emails were sent to members of APTA Geriatrics' Bone Health Special Interest Group. Nine physical therapists initiated the survey and 6 completed it. Users had up to 17 days to complete the survey and comment. The results of the survey responses are presented in Supplemental Digital Content Table 1, available at: https://links.lww.com/JGPT/A104, which summarizes invited expert and APTA Geriatrics Bone Health Special Interest Group members' levels of agreement. There were no additional comments provided by the user group.	Mitglieder der „APTA Geriatrics" wurden per E-Mail dazu eingeladen, den Entwurf zu prüfen und zu kommentieren. Weitere gezielte E-Mails wurden an Mitglieder der APTA Geriatrics-Interessengruppe für Knochengesundheit gesendet. Neun Physiotherapeut:innen begannen die Umfrage und 6 schlossen sie ab. Die Nutzer:innen hatten 17 Tage Zeit, um die Umfrage auszufüllen und zu kommentieren. Die Ergebnisse der Umfrageantworten sind in der Tabelle 1 des digitalen Zusatzmaterials aufgeführt, die unter https://links.lww.com/JGPT/A104 verfügbar ist und die Zustimmung der eingeladenen Expert:innen und Mitglieder der „APTA Geriatrics Bone Health Special Interest Group" zusammenfasst. Es wurden keine weiteren Kommentare abgegeben.
**Source guideline developer review**	**Begutachtung durch die Entwickler:innen der Originalleitlinie**
The guideline development group sent a copy of the adapted guideline to the source developer (SIGN) for feedback. They had no recommendations for changes aside from clarifying that the original SIGN CPG addressed falls and/or fracture risk/rate, but in this adapted document, those outcomes were not addressed. While SIGN does not endorse guidelines from other organizations, they did grant full consent to the SIGN guideline being used for the adaptation. (A. Stein, email communication, August 12, 2021.)	Die Leitlinienentwicklergruppe sandte eine Kopie der adaptierten Leitlinie an die Entwickler:innen der ursprünglichen Leitlinie (SIGN) zur Stellungnahme. Diese hatte keine Änderungsempfehlungen, außer der Klarstellung, dass die ursprüngliche SIGN-Leitlinie Stürze und/oder das Frakturrisiko/-rate behandelte, das adaptierte Dokument jedoch nicht. Obwohl SIGN keine Leitlinien anderer Organisationen befürwortet, erteilte sie die uneingeschränkte Zustimmung zur Verwendung der SIGN-Leitlinie für die Adaptation (A. Stein, E-Mail-Kommunikation, 12. August 2021).
**Endorsement**	**Befürwortung**

Original English Version	German Version
This CPG has been reviewed and endorsed by the medical and scientific advisory boards/councils of the following organizations:<br>American Bone Health (Raleigh, North Carolina)<br>National Osteoporosis Foundation (Arlington, Virginia)	Diese Leitlinie wurde von den medizinischen und wissenschaftlichen Beiräten/Gremien der folgenden Organisationen geprüft und befürwortet:<br>American Bone Health (Raleigh, North Carolina, USA)<br>National Osteoporosis Foundation (Arlington, Virginia, USA)
**Dissemination Plans**	**Verbreitungspläne**
The primary purpose of this CPG is to provide interested readers full documentation of the best available evidence for mitigating the loss of BMD for the management of patients with suspected or known osteoporosis. Publication of this guideline will be announced by press release and published in a high-impact, peer-reviewed journal. Education and awareness about this CPG will be disseminated via online resources, such as webinars and continuing education courses, at professional annual meetings and via social media. Pocket guides will be developed by APTA Geriatrics as implementation tools to aid in the dissemination of the CPG.	Der Hauptzweck dieser Leitlinie besteht darin, interessierten Leser:innen eine vollständige Dokumentation der besten verfügbaren Evidenz zur Minderung des Knochendichteverlusts bei der Behandlung von Patient:innen mit vermuteter oder bestätigter Osteoporose zur Verfügung zu stellen. Die Veröffentlichung dieser Leitlinie wird in einer Pressemitteilung bekannt gegeben und in einem renommierten, von Expert:innen begutachteten Fachjournal veröffentlicht. Die Informationen und das Bewusstsein für diese Leitlinie werden über Online-Ressourcen wie Webinare und Weiterbildungskurse, auf jährlichen Fachtagungen und über soziale Medien verbreitet. „APTA Geriatrics" wird als Hilfsmittel zur Umsetzung Leitlinien-Kitteltaschenversionen entwickeln, um die Verbreitung der Leitlinie zu fördern.
**Revision and Reaffirmation Plans**	**Pläne zur Überarbeitung und Qualitätssicherung**
This CPG represents a cross-sectional view of current treatment and may become outdated as new evidence becomes available. It will be reviewed in 5 years and will be updated in accordance with new evidence, changing practice, rapidly emerging treatment options, and new technology, reaffirmed, or withdrawn.	Diese Leitlinie stellt eine Querschnittsansicht der aktuellen Behandlung dar und kann veralten, wenn neue Evidenz verfügbar wird. Sie wird in 5 Jahren überprüft und entsprechend neuer Evidenz, sich ändernder Praxis, neu aufkommender Behandlungsoptionen und neuer Technologie aktualisiert, bestätigt oder zurückgezogen.
SUMMARY OF RECOMMENDATIONS	ZUSAMMENFASSUNG DER EMPFEHLUNGEN
Exercise Recommendations for Postmenopausal Women to Slow Decline of Bone Mineral Density of the Hip and the Femoral Neck: Physical therapists should design and advise post-menopausal women to participate in long-duration exercise programs consisting of static weight-bearing exercises such as single-leg standing to slow the decline of bone mineral density (BMD) at the hip. Physical therapists should design and advise postmenopausal women to participate in long-duration, adequately dosed progressive-resistance strength training exercises such as weight training either alone or in combina- tion with impact exercise training such as jogging, walking, or aerobics to slow the decline of BMD of the femoral neck. (Grade B, recommendation based on moderate evidence)	Trainingsempfehlungen für Frauen nach der Menopause zur Verlangsamung des Rückgangs der Knochendichte im proximalen Oberschenkelknochen und des Oberschenkelhalses: Physiotherapeut:innen sollten langfristige Trainingsprogramme für Frauen nach der Menopause zusammenstellen und anleiten, die aus statischem, gewichtstragendem Training bestehen, wie z.B. dem Einbeinstand, um den Rückgang der Knochendichte an der Hüfte zu verlangsamen. Physiotherapeut:innen sollten für Frauen nach der Menopause ein langfristiges, angemessen dosiertes, progressives Krafttraining, zum Beispiel mit Gewichten, zusammenstellen und anleiten. Diese Trainingsprogramme sollten alleine oder in Kombination mit einem Impact-Training, wie z.B. Joggen, Walken oder Aerobic durchgeführt werden, um den Rückgang der Knochendichte des Oberschenkelhalses zu verlangsamen. (Empfehlungsgrad B, moderate Qualität der Evidenz)

Original English Version	German Version
Exercise Recommendations for Postmenopausal Women to Slow Decline of Bone Mineral Density of the Lumbar Spine: Physical therapists should consider designing and advising postmeno- pausal women to participate in long-duration exercise programs consisting of walking, tai chi, progressive-resistance strength training (such as weight training), and different combinations of exercise types to slow the decline of lumbar spine BMD. (Grade B, recommendation based on moderate evidence)	Trainingsempfehlungen für Frauen nach der Menopause zur Verlangsamung des Rückgangs der Knochendichte der Lendenwirbelsäule: Physiotherapeut:innen sollten in Erwägung ziehen, für Frauen nach der Menopause langfristige Trainingsprogramme, bestehend aus Gehen, Tai-Chi, progressivem Krafttraining (zum Beispiel mit Gewichten) und verschiedenen Kombinationen von Trainingsformen zusammenzustellen und anzuleiten, um den Rückgang der Knochendichte der Lendenwirbelsäule zu verlangsamen. (Empfehlungsgrad B, moderate Qualität der Evidenz)
Exercise Recommendations for Premenopausal Women to Slow Decline of Bone Mineral Density of the Femoral Neck: Physical therapists should consider designing and advis- ing premenopausal women to participate in long-duration exercise programs consisting of high-impact exercise (such as jogging) and combining impact exercise (such as stair climbing) with progressive-resistance strength training (such as weight training) to slow the decline of femoral neck BMD. (Grade B, recommendation based on moderate evidence)	Trainingsempfehlungen für Frauen vor der Menopause zur Verlangsamung des Rückgangs der Knochendichte des Oberschenkelhalses: Physiotherapeut:innen sollten in Erwägung ziehen, für Frauen vor der Menopause langfristige Trainingsprogramme, bestehend aus Trainingsformen mit „high-impact“ Belastung (wie Joggen) und der Kombination von Impact-Training (wie Treppensteigen) mit progressivem Krafttraining (zum Beispiel mit Gewichten) zusammenzustellen und anzuleiten, um den Rückgang der Knochendichte am Oberschenkelhals zu verlangsamen. (Empfehlungsgrad B, moderate Qualität der Evidenz)
Exercise Recommendations for Premenopausal Women to Slow Decline of Bone Mineral Density of the Lumbar Spine: Physical therapists should consider designing and advising premenopausal women to participate in long-duration exer- cise programs consisting of progressive-resistance strength training (such as weight training) alone or in combination with impact exercises (such as stair climbing or jogging) to slow the decline of lumbar spine BMD. (Grade B, recom- mendation based on moderate evidence)<br>Exercise Recommendations for Men: There is insufficient evidence to make a recommendation for exercise for improving BMD in men.	Trainingsempfehlungen für Frauen vor der Menopause zur Verlangsamung des Rückgangs der Knochendichte der Lendenwirbelsäule: Physiotherapeut:innen sollten in Erwägung ziehen, für Frauen vor der Menopause langfristige Trainingsprogramme, bestehend aus progressivem Krafttraining (zum Beispiel mit Gewichten) allein oder in Kombination mit Impact-Training (wie Treppensteigen oder Joggen) zusammenzustellen und anzuleiten, um den Rückgang der Knochendichte der Lendenwirbelsäule zu verlangsamen. (Empfehlungsgrad B, moderate Qualität der Evidenz)
The author contributions and acknowledgments are as follows: concept/idea/research design: Avin, Roach, and Hartley; writing: Hartley, Roach, Avin, Nithman, and Fuchs; data collection: Avin, Roach, Hartley, Betz, Lindsey, and Nithman; data analysis: Hartley, Avin, Roach, Betz, Lindsey, and Nithman; quality reviews of the literature: Avin, Hartley, Roach, Nithman, Betz, and Lindsey; project management: Avin; fund procurement: Avin; providing institutional liaisons: N/A; and consultation (including review of manuscript before submitting): Hartley, Avin, Roach, Betz, Nithman, and Lindsey.	Die Beiträge und Danksagungen der Autor:innen lauten wie folgt: Konzept/Idee/Forschungsdesign: Avin, Roach und Hartley; Schreiben: Hartley, Roach, Avin, Nithman und Fuchs; Datenerhebung: Avin, Roach, Hartley, Betz, Lindsey und Nithman; Datenanalyse: Hartley, Avin, Roach, Betz, Lindsey und Nithman; Qualitätsprüfungen der Literatur: Avin, Hartley, Roach, Nithman, Betz und Lindsey; Projektmanagement: Avin; Mittelbeschaffung: Avin; Bereitstellung von institutionellen Ansprechpartnern: N/A; und Beratung (einschließlich Überprüfung des Manuskripts vor der Einreichung): Hartley, Avin, Roach, Betz, Nithman und Lindsey.
This clinical practice guideline (CPG) was supported by a grant from the American Physical Therapy Association and by the American Physical Therapy Association Academy of Geriatric Physical Therapy (APTA Geriatrics), which received no funding from outside commercial sources to support the guideline’s development. The views of the funding body have not influenced the content of the guideline.	Diese Leitlinie wurde durch eine Förderung der „American Physical Therapy Association“ und der „American Physical Therapy Association Academy of Geriatric Physical Therapy“ (APTA Geriatrics) unterstützt, die keine Mittel von externen kommerziellen Quellen zur Unterstützung der Entwicklung der Leitlinie erhalten haben. Die Ansichten der finanzierenden Stelle haben den Inhalt der Leitlinie nicht beeinflusst.

Original English Version	German Version
All individuals whose names appear as authors of or contributors to this CPG filed a disclosure statement as part of the grant submission process. In addition, prior to and during the development of this CPG, development group members routinely disclosed conflicts of interest in writing to APTA Geriatrics using the International Committee of Medical Journal Editors (ICMJE) Form for Disclosure of Potential Conflicts of Interest. Each of the guideline development group (GDG) members was asked to disclose any existing or potential conflicts of interest—including financial relationships with pharmaceutical, medical device, or biotechnology companies—prior to being included in the group and routinely thereafter.	Alle Personen, deren Namen als Autor:innen oder Mitwirkende dieser Leitlinie erscheinen, reichten im Rahmen des Antragsverfahrens eine Offenlegungserklärung ein. Darüber hinaus legten die Mitglieder der Entwicklungsgruppe vor und während der Entwicklung dieser Leitlinie routinemäßig Interessenkonflikte schriftlich gegenüber „APTA Geriatrics“ unter Verwendung des Formulars des „International Committee of Medical Journal Editors“ (ICMJE) offen. Jedes Mitglied der Leitlinienentwicklungsgruppe wurde vor der Aufnahme in die Gruppe und danach routinemäßig gebeten, bestehende oder potenzielle Interessenkonflikte offenzulegen – einschließlich finanzieller Beziehungen zu Pharma-, Medizinprodukte- oder Biotechnologieunternehmen.
All guideline development group members report grants from APTA Geriatrics and APTA during the conduct of the work. Dr Hartley served as a member of the board of directors of APTA Geriatrics from during the conduct of this work. He received no compensation for this position; however, the Academy funded travel to annual meeting(s). Dr Betz served as chair of the Bone Health Special Interest Group of APTA Geriatrics from 2009 to 2020. She received no compensation for this position; however, the Academy funded some expenses related to travel to an annual meeting.	Alle Mitglieder der Leitlinienentwicklungsgruppe berichten über Zuschüsse von „APTA Geriatrics“ und „APTA“ während der Durchführung der Arbeit. Dr. Hartley war während der Durchführung dieser Arbeit Mitglied des Vorstands von „APTA Geriatrics“. Er erhielt keine Vergütung für diese Position; die Akademie finanzierte jedoch die Reise zu Jahrestagung(en). Dr. Betz war von 2009 bis 2020 Vorsitzende der „Bone Health Special Interest Group“ von „APTA Geriatrics“. Sie erhielt keine Vergütung für diese Position; die Akademie finanzierte jedoch einige Ausgaben im Zusammenhang mit der Reise zu einer Jahrestagung.
Supplemental digital content is available for this article. Direct URL citations appear in the printed text and are provided in the HTML and PDF versions of this article on the journal's Web site (www.jgptonline.com).	Zum Artikel der Original-Leitlinie (https://www.doi.org/10.1519/JPT.0000000000000346) sind ergänzende digitale Inhalte verfügbar. Direkte URL-Zitate erscheinen in der gedruckten Version und sind in den HTML- und PDF-Versionen dieses Artikels auf der Website der Zeitschrift „Journal of Geriatric Physical Therapy“ (www.jgptonline.com) verfügbar.
This is an open-access article distributed under the terms of the Creative Commons Attribution-Non Commercial-No Derivatives License 4.0 (CCBY-NC-ND), where it is permissible to download and share the work provided it is properly cited. The work cannot be changed in any way or used commercially without permission from the journal.	Die Original-Leitlinie (https://www.doi.org/10.1519/JPT.0000000000000346) ist ein Open-Access-Artikel, der unter den Bedingungen der Creative Commons Attribution-Non Commercial-No Derivatives License 4.0 (CCBY-NC-ND) verbreitet wird, wonach es erlaubt ist, die Arbeit herunterzuladen und zu teilen, sofern sie ordnungsgemäß zitiert wird. Die Arbeit darf ohne Erlaubnis der Zeitschrift in keiner Weise verändert oder kommerziell genutzt werden.
Address correspondence to: Keith G. Avin, PT, DPT, PhD, Department of Physical Therapy, School of Health & Human Sciences, Indiana University, CF-326, 1140 W Michigan St, Indianapolis, IN 46202 (keigavin@iu.edu).	Korrespondenzadresse: Keith G. Avin, PT, DPT, PhD, Department of Physical Therapy, School of Health & Human Sciences, Indiana University, CF-326, 1140 W Michigan St, Indianapolis, IN 46202 (keigavin@iu.edu).
Copyright © 2022 The Authors. Published by Wolters Kluwer Health, Inc. on behalf of Academy of Geriatric Physical Therapy, APTA.	Copyright © 2022 Die Autor:innen. Veröffentlicht von Wolters Kluwer Health, Inc. im Auftrag der Academy of Geriatric Physical Therapy, APTA.

Original English Version	German Version
Table 1. AGREE II Rigor and Quality Scores for Osteoporosis Clinical Practice Guidelines	Tabelle 1. AGREE II Bewertungen der wissenschaftliche Strenge und Qualität von Leitlinien für Osteoporose
Guideline Title	Titel der Leitlinie
AGREE II Rigor Score	AGREE II wissenschaftliche Strenge-Score
AGREE II Quality Score	AGREE II Qualitäts-Score
Management of osteoporosis and the prevention of fragility fractures. Scottish Intercollegiate Guidelines Network (SIGN). (Scotland, 2015, updated 2020 and 2021)	Management of osteoporosis and the prevention of fragility fractures. Scottish Intercollegiate Guidelines Network (SIGN). (Schottland, 2015, aktualisiert 2020 und 2021) [21]
Too Fit to Fracture: Exercise recommendations for individuals with osteoporosis or osteoporotic vertebral fracture. International Osteoporosis Foundation and National Osteoporosis Foundation. (Canada, 2013)	Too Fit to Fracture: Exercise recommendations for individuals with osteoporosis or osteoporotic vertebral fracture. International Osteoporosis Foundation and National Osteoporosis Foundation. (Kanada, 2013) [27]
National Osteoporosis Guideline Group (NOGG) 2017: Clinical guideline for the prevention and treatment of osteoporosis. National Institute for Health and Care Excellence (NICE) accredited. (Britain/United Kingdom, 2017)	National Osteoporosis Guideline Group (NOGG) 2017: Clinical guideline for the prevention and treatment of osteoporosis. National Institute for Health and Care Excellence (NICE) accredited. (Großbritannien/Vereinigtes Königreich, 2017) [26]
2015 Guidelines for Osteoporosis in Saudi Arabia: Recommendations from the Saudi Osteoporosis Society. (Saudi Arabia, 2015)	2015 Guidelines for Osteoporosis in Saudi Arabia: Recommendations from the Saudi Osteoporosis Society. (Saudi-Arabien, 2015) [25]
Clinical guidelines for the prevention and treatment of osteoporosis: Summary statements and recommendations from the Italian Society of Orthopedics and Traumatology. (Italy, 2017)	Clinical guidelines for the prevention and treatment of osteoporosis: Summary statements and recommendations from the Italian Society of Orthopedics and Traumatology. (Italien, 2017) [23]
Taiwanese Guidelines for the Prevention and Treatment of Osteoporosis. (Taiwan, 2014)	Taiwanese Guidelines for the Prevention and Treatment of Osteoporosis. (Taiwan, 2014) [24]
Abbreviation: AGREE, Appraisal of Guidelines for Research & Evaluation.	Abkürzung: AGREE, Appraisal of Guidelines for Research & Evaluation

Original English Version	German Version
Table 2. Exercise Interventions Definitions	Tabelle 2. Trainingsformen
Exercise	Trainingsform [33]
Definition	Beispiele
SWB Static weight-bearing exercise including single-leg standing.	Statisches gewichtstragendes Training | Einbeinstand
DWBLF Dynamic weight-bearing exercise with low force including walking and tai chi.	Dynamisches gewichtstragendes Training mit geringer Krafteinwirkung | Gehen und Tai-Chi
DWBHF Dynamic weight-bearing exercise with high force including jogging, jumping, running, dancing, and vibration platform.	Dynamisches gewichtstragendes Training mit hoher Krafteinwirkung | Joggen, Springen, Laufen, Tanzen und Vibrationstraining
NWBLF Non–weight-bearing exercise with low force, eg, low-load high-repetition strength training.	Nicht gewichtstragendes Training mit geringer Krafteinwirkung | Krafttraining mit hoher Wiederholungszahl und niedrigem Widerstand
NWBHF Non–weight-bearing exercise high force, eg, progressive resisted strength training.	Nicht gewichtstragendes Training mit hoher Krafteinwirkung | progressives Krafttraining
COMB Combination of exercise types with more than one of the above exercise interventions.	Kombination verschiedener Trainingsformen | Kombination mit mehr als einer der oben genannten Trainingsformen

Original English Version	German Version
Table 3. Grades/Strength of Recommendation	Tabelle 3. Grade/Stärke der Empfehlung
Letter Grade	Grad der Empfehlung
Strength of Recommendation	Stärke der Empfehlung
Definition	Definition
A<br>Strong<br>A high level of certainty of moderate to substantial benefit, harm, or cost, or a moderate level of certainty for substantial benefit, harm, or cost (based on a preponderance of level 1 or 2 evidence with at least 1 level 1 study).	A<br>Stark<br>Ein hohes Maß an Vertrauen in einen mäßigen bis erheblichen Nutzen, Schaden oder für Kosten, oder ein mittleres Maß an Vertrauen in einen erheblichen Nutzen, Schaden oder Kosten (basierend auf ein Überwiegen von Evidenz der Stufe 1 oder 2 mit mindestens einer Studie der Stufe 1).
B<br>Moderate<br>A high level of certainty of slight to moderate benefit, harm, or cost, or a moderate level of certainty for a moderate level of benefit, harm, or cost (based on a preponderance of level 2 evidence, or a single high-quality RCT).	B<br>Moderat<br>Ein hohes Maß an Vertrauen in einen geringen bis mäßigen Nutzen, Schaden oder Kosten, oder ein mittleres Maß an Vertrauen in einen mäßigen Nutzen, Schaden oder Kosten (basierend auf ein Überwiegen von Evidenz der Stufe 2 oder einer einzelnen hochwertigen RCT).
C<br>Weak<br>A moderate level of certainty of slight benefit, harm, or cost, or a weak level of certainty for moderate to substantial benefit, harm, or cost (based on level 2 through 5 evidence).	C<br>Schwach<br>Ein moderater Grad an Vertrauen in einen geringen Nutzen, Schaden oder Kosten oder ein schwacher Grad an Vertrauen in einen mäßigen bis erheblichen Nutzen, Schaden oder Kosten (basierend auf Evidenz der Stufen 2 bis 5).
D<br>Theoretical/foundational<br>A preponderance of evidence from animal or cadaver studies, from conceptual/theoretical models/principles, or from basic science/bench research, or published expert opinion in peer-reviewed journals that supports the recommendation.	D<br>Theoretisch/grundlegend<br>Evidenz überwiegend aus Tier- oder Kadaverstudien, aus konzeptuellen/theoretischen Modellen/Prinzipien oder aus Grundlagenforschung/Laborforschung oder veröffentlichten Expertenmeinungen in Fachzeitschriften mit Peer-Review, die die Empfehlung unterstützen.
P<br>Best practice<br>Recommended practice based on current clinical practice norms, exceptional situations in which validating studies have not or cannot be performed; yet, there is a clear benefit, harm, or cost, expert opinion.	P<br>Best Practice<br>Empfohlene Praxis basierend auf aktuell bewährten klinischen Vorgehensweisen, außergewöhnlichen Situationen, in denen Validierungsstudien nicht durchgeführt wurden oder nicht durchgeführt werden können; dennoch gibt es einen klaren Nutzen, Schaden oder Kosten, Expertenmeinung.
R	R

Original English Version	German Version
Research<br>An absence of research on the topic or disagreement among conclusions from high-quality studies on the topic.	Forschung<br>Fehlen von Forschungsarbeiten zum Thema oder Uneinigkeit über die Schlussfolgerungen hochwertiger Studien zu diesem Thema.
Abbreviation: RCT, randomized clinical trial.	Abkürzung: RCT, randomisierte klinische Studie

Original English Version	German Version
Table 4. Detailed Information on Exercise Interventions for Postmenopausal Women	Tabelle 4. Detaillierte Informationen zu Trainingsinterventionen für Frauen nach der Menopause
Exercise Type	Trainingsform
Exercise	Training
Exercise Dose	Trainingsumfang
Participants	Teilnehmende
Adverse Events	Unerwünschte Ereignisse
Evidence Summary	Zusammenfassung der Evidenz
Static weight bearing	Statisches gewichtstragendes Training [33]
Single-leg standing	Einbeinstand
1 min per limb 3 times per day 24 wk	▪ 1 Minute pro Bein<br>▪ 3-mal täglich<br>▪ 24 Wochen
94 Japanese women, mean age: 68 y (age range: 61-85 y)	▪ 94 Frauen aus Japan<br>▪ Durchschnittsalter: 68 Jahre (Altersspanne: 61-85 Jahre)
None reported	Keine berichtet
Evidence supported a modest positive impact of static weight-bearing exercises on hip BMD	Es gibt Evidenz für eine geringe positive Auswirkung von statischem Training mit Gewichtsbelastung auf die Knochendichte der Hüfte.
Dynamic weight bearing: Low force	Dynamisches gewichtstragendes Training: geringe Krafteinwirkung [33, 36, 37]
Walking, tai chi	Gehen, Tai-Chi
50-60 min 3 times per week 40-54 wk	▪ 50-60 Minuten<br>▪ 3-mal pro Woche<br>▪ 40-54 Wochen
Healthy postmenopausal women, women with UE fracture, women with osteopenia, Age: 46-92 y, North America, Europe, Asia, and Australia	▪ Gesunde Frauen nach der Menopause, Frauen mit Fraktur der oberen Extremität, Frauen mit Osteopenie<br>▪ Alter: 46-92 Jahre<br>▪ Nordamerika, Europa, Asien und Australien
Exercise and control groups reported injuries. One exercise-specific injury reported	Berichte über Verletzungen sowohl in der Trainings- als auch der Kontrollgruppe. Eine trainingsbezogene Verletzung berichtet.
Evidence supported weight-bearing low-force exercises to slow spine BMD loss. Evidence did not support weight-bearing low-force exercises to increase spine BMD.	Es gibt Evidenz dafür, dass gewichtstragendes Training mit geringer Krafteinwirkung den Knochendichte-Verlust der Wirbelsäule verlangsamen.<br>Es gibt keine Evidenz dafür, dass gewichtstragendes Training mit geringer Krafteinwirkung die Knochendichte der Wirbelsäule erhöht.

Original English Version	German Version
Dynamic weight bearing: High force	Dynamisches gewichtstragendes Training: hohe Krafteinwirkung [33, 38]
Standing weight lifting, Walking/jogging or hopping, Stair climbing with weighted vests, jumping, running, dancing, vibration platforms	Gewichte heben im Stehen<br>Gehen/Joggen oder Hüpfen<br>Treppensteigen mit Gewichtswesten, Springen, Laufen, Tanzen, Vibrationsplattformen
50-55 min per session 2-3 d/wk 52 wk	▪ 50-55 Minuten pro Einheit<br>▪ 2-3 Tage/Woche<br>▪ 52 Wochen
Healthy postmenopausal women, women with osteopenia or osteoporosis, Age: 40-65 y, United States	▪ Gesunde Frauen nach der Menopause, Frauen mit Osteopenie oder Osteoporose<br>▪ Alter: 40-65 Jahre<br>▪ USA
One injury reported in an intervention group	Eine Verletzung in einer Interventionsgruppe wurde berichtet.
Evidence did not support dynamic weight bearing: high force exercises to improve or maintain BMD at the spine or the hip	Es gibt keine Evidenz dafür, dass dynamisches, gewichtstragendes Training mit hoher Krafteinwirkung zur Verbesserung oder zum Erhalt der Knochendichte an der Wirbelsäule oder der Hüfte führt.
Non–weight bearing: Low force	Nicht gewichtstragendes Training: geringe Krafteinwirkung [33]
Seated low-load, high-repetition strength training	Krafttraining im Sitzen mit geringem Widerstand und hoher Wiederholungszahl
~40% 1RM 1-3 sets of 10-20 repetitions 3-7 times per week 24-104 wk	▪ ~40% 1RM<br>▪ 1-3 Sätze mit 10-20 Wiederholungen<br>▪ 3–7-mal pro Woche<br>▪ 24-104 Wochen
Healthy postmenopausal women, Age: 41-82 y	▪ Gesunde Frauen nach der Menopause<br>▪ Alter: 41-82 Jahre
None reported	Keine berichtet.
Evidence did not support non–weight-bearing low-force exercises in nonosteoporotic postmenopausal women	Es gibt keine Evidenz für den Nutzen eines nicht gewichtstragenden Trainings mit geringer Krafteinwirkung bei Frauen nach der Menopause, die keine Osteoporose haben.
Non–weight bearing: High force	Nicht gewichtstragendes Training: hohe Krafteinwirkung [33, 39]
Seated progressive resistive, high-load strength training<br>Some programs included both upper and lower extremity exercise	Progressives Krafttraining im Sitzen mit hohem Widerstand<br>Einige Programme umfassten sowohl Übungen für die obere als auch für die untere Extremität
1-3 sets ~70%-85% 1RM 2-3 times per week 24-104 wk	▪ 1-3 Sätze<br>▪ ~70%-85% 1RM<br>▪ 2–3-mal pro Woche<br>▪ 24-104 Wochen

Original English Version	German Version
Healthy postmenopausal women, Age: 41-75 y, North and South America and Australia, 2 studies excluded women with osteoporosis	▪ Gesunde Frauen nach der Menopause<br>▪ Alter: 41-75 Jahre<br>▪ Nord- und Südamerika und Australien<br>▪ 2 Studien schlossen Frauen mit Osteoporose aus
None reported	Keine berichtet.
Evidence supported modest positive impact of non–weight-bearing high-force exercises on BMD at both the spine and the femoral neck	Es gibt Evidenz für eine geringe positive Auswirkung von nicht gewichtstragendem Training mit hoher Krafteinwirkung auf die Knochendichte sowohl an der Wirbelsäule als auch am Oberschenkelhals.
Combination of exercise types	Kombination von Trainingsformen [33, 37, 38, 40-42]
Combinations of any 2 of the exercise types described previously	Kombinationen von 2 beliebigen der zuvor beschriebenen Trainingsformen
2-7 d/wk 52 wk Intensity not reported	▪ 2-7 Tage/Woche<br>▪ 52 Wochen<br>▪ Intensität nicht angegeben
Postmenopausal women, Age: 53-87 y, Europe and Asia, 2 studies included women with osteoporosis	▪ Frauen nach der Menopause<br>▪ Alter: 53-87 Jahre<br>▪ Europa und Asien<br>▪ 2 Studien schlossen Frauen mit Osteoporose ein
None reported	Keine berichtet.
Evidence supported a modest positive impact of combination exercises on BMD at both the spine and the femoral neck<br>Evidence also suggested that combination exercise programs substantially reduced the risk of fractures	Es gibt Evidenz für eine geringe positive Auswirkung von kombiniertem Training auf die Knochendichte sowohl an der Wirbelsäule als auch am Oberschenkelhals.<br>Es gibt auch Hinweise darauf, dass kombinierte Trainingsprogramme das Frakturrisiko erheblich senken.
Abbreviations: BMD, bone mineral density; RM, repetition maximum; UE, upper extremity.	Abkürzung: 1RM, 1-Wiederholungsmaximum

Original English Version	German Version
Table 5. - Detailed Information on Exercise Interventions for Premenopausal Women	Tabelle 5. Detaillierte Informationen zu Trainingsinterventionen für Frauen vor der Menopause
Exercise Type	Trainingsform
Exercise	Training
Exercise Dose	Trainingsumfang
Participants	Teilnehmende
Adverse Events	Unerwünschte Ereignisse
Evidence Summary	Zusammenfassung der Evidenz
Static weight bearing	Statisches gewichtstragendes Training
Single-leg standing	Einbeinstand
No systematic reviews available	Keine systematischen Reviews verfügbar
Not applicable	Nicht anwendbar
Not applicable	Nicht anwendbar
There is insufficient evidence to make any conclusion about this type of exercise specifically for BMD	Es gibt nicht genügend Evidenz, um eine Aussage über diese Trainingsform speziell für die Knochendichte zu treffen.
Dynamic weight bearing: low force	Dynamisches gewichtstragendes Training: geringe Krafteinwirkung [47]
Walking, tai chi	Gehen, Tai-Chi
No systematic reviews available	Keine systematischen Reviews verfügbar
Not applicable	Nicht anwendbar
Not applicable	Nicht anwendbar
There is insufficient evidence to make any conclusion about this type of exercise specifically for BMD	Es gibt nicht genügend Evidenz, um eine Aussage über diese Trainingsform speziell für die Knochendichte zu treffen.
Dynamic weight bearing: high force	Dynamisches gewichtstragendes Training: hohe Krafteinwirkung [48]
Jumping, jogging, skipping, running, dancing, vibration platforms	Springen, Joggen, Hüpfen, Laufen, Tanzen, Vibrationsplattformen
Jumping 5 sets of 10 repetitions 100 jumps per day 40-min skipping<br>Jumping with weighted vest 2-6 d/wk 6-18 mo High-impact aerobics<br>60 min/session 3 times per week 1-2 y	*Springen*<br>▪ 5 Sätze mit 10 Wiederholungen<br>▪ 100 Sprünge pro Tag<br>▪ 40 min Springen<br>▪ Springen mit Gewichtsweste 2-6 Tage/Woche<br>▪ 6-18 Monate<br>*High-Impact-Aerobics*<br>▪ 60 min/Sitzung

Original English Version	German Version
	▪ 3-mal pro Woche<br>▪ 1-2 Jahre
Healthy, premenopausal women Age: 20-48 y, North America, Asia, and Europe	▪ Gesunde, Frauen vor der Menopause<br>▪ Alter: 20-48 Jahre<br>▪ Nordamerika, Asien und Europa
None reported	Keine berichtet
Evidence supported the positive impact of dynamic weight-bearing high-force exercises in reducing BMD decline only at the femoral neck	Es gibt Evidenz für die positive Auswirkung von dynamischem, gewichtstragendem Training mit hoher Krafteinwirkung auf die Verringerung des Knochendichte-Rückgangs ausschließlich am Oberschenkelhals.
Non–weight bearing: Low force	Nicht gewichtstragendes Training: geringe Krafteinwirkung
Progressive resistive, low-load strength training	Progressives Krafttraining mit geringem Widerstand
No systematic reviews available	Keine systematischen Reviews verfügbar
Not applicable	Nicht anwendbar
Not applicable	Nicht anwendbar
There is insufficient evidence to make any conclusion about this type of exercise specifically for BMD	Es gibt nicht genügend Evidenz, um eine Aussage über diese Trainingsform speziell für die Knochendichte zu treffen.
Non–weight bearing: High force	Nicht gewichtstragendes Training: hohe Krafteinwirkung [49]
Seated progressive resistive, high-load strength training	Progressives Krafttraining im Sitzen mit hohem Widerstand<br>Einige Programme umfassten sowohl Übungen für die obere als auch für die untere Extremität
60% 1RM 5 sets of 20 repetitions 2-3 d/wk 5-18 mo	▪ 60% 1RM<br>▪ 5 Sätze à 20 Wiederholungen<br>▪ 2-3 Tage/Woche<br>▪ 5-18 Monate
Healthy, premenopausal women, Age: 20-50 y, North America, Some studies excluded women on oral contraceptives	▪ Gesunde Frauen vor der Menopause<br>▪ Alter: 20-50 Jahre<br>▪ Nordamerika<br>▪ Einige Studien schlossen Frauen aus, die orale Kontrazeptiva einnahmen
None reported	Keine berichtet
Evidence supported modest positive impact of non–weight-bearing high-force exercises on BMD at the lumbar spine but not the femoral neck	Es gibt Evidenz für eine geringe positive Auswirkung von nicht gewichtstragendem Training mit hoher Krafteinwirkung auf die Knochendichte an der Lendenwirbelsäule, nicht aber am Oberschenkelhals.
Combination of exercise types	Kombination von Trainingsformen [48]

Original English Version	German Version
Circuit strength and high-impact aerobics, Skipping and strength training, Resistance training and jumping	Zirkeltraining und „high-impact“-Aerobics<br>Springen und Krafttraining
2-9 sets of 8-12 repetitions 3 d/wk 12 mo	▪ 2-9 Sätze mit 8-12 Wiederholungen<br>▪ 3 Tage/Woche<br>▪ 12 Monate
Healthy premenopausal women., Age: 24-41 y, North America	▪ Gesunde Frauen vor der Menopause<br>▪ Alter: 24-41 Jahre<br>▪ Nordamerika
None reported	Keine berichtet
Evidence supported that combination exercises were effective in increasing BMD at the femoral neck and the lumbar spine	Die Evidenz belegt die Wirksamkeit von Kombinationstraining, um die Knochendichte am Oberschenkelhals und an der Lendenwirbelsäule zu erhöhen.
Abbreviations: BMD, bone mineral density; RM, repetition maximum.	Abkürzung: 1RM, 1-Wiederholungsmaximum

Original English Version	German Version
Figure: Phases, modules, and steps in the adaptation process. From the ADAPTE Collaboration, Version 2.0.29 This figure is available in color online (www jgeript.org).	Abbildung 1. Phasen, Module und Schritte im Adaptionsprozess. Von der ADAPTE Collaboration, Version 2.0.29 [29]
Phase 1: Set-Up	Phase 1: Vorbereitung
Preparation Module	Modul Vorbereitung
Step 1. Establish an organizing committee	Schritt 1. Gründung eines Organisationskomitees
Step 2. Select a guideline topic	Schritt 2. Auswahl eines Leitlinienthemas
Step 3. Check whether adaptation is feasible	Schritt 3. Prüfung der Durchführbarkeit der Adaption
Step 4. Identify necessary resources and skills	Schritt 4. Identifizierung notwendiger Ressourcen und Kompetenzen
Step 5. Complete tasks for the Set-Up phase	Schritt 5. Abschluss der Aufgaben für die Vorbereitungsphase
Step 6. Write an adaptation plan	Schritt 6. Erstellung eines Adaptionsplans
Phase 2: Adaptation	Phase 2: Adaption
Scope and Purpose Module	Modul Umfang- und Zweck
Step 7. Determine the health/clinical/policy questions	Schritt 7. Festlegung der gesundheitlichen/klinischen/politischen Fragestellungen
Search and Screen Module	Modul Literatursuche und -auswahl
Step 8. Search for guidelines and other relevant documents	Schritt 8. Suche nach Leitlinien und anderen relevanten Dokumenten
Step 9. Screen retrieved guidelines	Schritt 9. Screening der gefundenen Leitlinien
Step 10. Reduce a large number of retrieved guidelines	Schritt 10. Reduzierung einer großen Anzahl gefundener Leitlinien
Assessment Module	Modul Bewertung
Step11. Assess guideline quality	Schritt 11. Bewertung der Leitlinienqualität
Step 12. Assess guideline currency	Schritt 12. Bewertung der Aktualität der Leitlinien
Step 13. Assess guideline content	Schritt 13. Bewertung des Leitlinieninhalts
Step 14. Assess guideline consistency	Schritt 14. Bewertung der Leitlinienkonsistenz
Step 15. Assess acceptability/applicability of the recommendations	Schritt 15. Bewertung der Akzeptanz/Anwendbarkeit der Empfehlungen
Decision and Selection Module	Modul Entscheidung und Auswahl
Step 16. Review assessments	Schritt 16. Überprüfung der Bewertungen
Step 17. Select among guidelines and recommendations to create an adapted guideline	Schritt 17. Auswahl aus Leitlinien und Empfehlungen zur Erstellung einer adaptierten Leitlinie
Customization Module	Modul Anpassung
Step 18. Prepare draft adapted guideline	Schritt 18. Erstellung eines Entwurfs der adaptierten Leitlinie
Phase 3: Finalization	Phase 3: Fertigstellung
External review and Achknowledgment Module	Modul Externes Review und Danksagung
Step 19. External review by target users	Schritt 19. Externes Review durch Zielnutzer:innen
Step 20. Consult with relevant endorsement bodies	Schritt 20. Konsultation relevanter Gremien zur Befürwortung

Original English Version	German Version
Step 21. Consult with developers of source guidelines	Schritt 21. Konsultation der Entwickler:innen der Quellleitlinien
Step 22. Acknowledge source documents	Schritt 22. Anerkennung der Quelldokumente
Aftercare Planning Module	Modul Nachsorge
Step 23. Plan scheduled reviewand update of adapted guideline	Schritt 23. Planung regelmäßiger Überprüfungen und Aktualisierungen der adaptierten Leitlinie
Final Production Module	Modul Endproduktion
Step 24. Produce final guidance document	Schritt 24. Erstellung des endgültigen Leitliniendokuments
